# Translating DREAMS into practice: Early lessons from implementation in six settings

**DOI:** 10.1371/journal.pone.0208243

**Published:** 2018-12-13

**Authors:** Natsayi Chimbindi, Isolde Birdthistle, Maryam Shahmanesh, Jane Osindo, Phillis Mushati, Kenneth Ondeng’e, Thembelihle Zuma, Tarisai Chiyaka, Nambusi Kyegombe, James Hargreaves, Joanna Busza, Sian Floyd, Janet Seeley

**Affiliations:** 1 Africa Health Research Institute, Durban, South Africa; 2 Faculty of Epidemiology and Population Health, London School of Hygiene & Tropical Medicine, London, United Kingdom; 3 Institute of Global Health, University College London, London, United Kingdom; 4 African Population and Health Research Center, Nairobi, Kenya; 5 Centre for Sexual Health and HIV/AIDS Research, Harare, Zimbabwe; 6 Centre for Global Health Research, Kenya Medical Research Institute, Kisumu, Kenya; 7 Faculty of Public Health & Policy, London School of Hygiene & Tropical Medicine, London, United Kingdom; UNAIDS, UNITED STATES

## Abstract

**Background:**

The ‘DREAMS Partnership’ promotes a multi-sectoral approach to reduce adolescent girls and young women’s (AGYW) vulnerability through a core package of interventions targeting multiple sources of HIV risk–to promote Determined, Resilient, Empowered, AIDS-free, Mentored and Safe (DREAMS) lives. Implementation of such multi-sectoral programmes is complex and requires adaptation to national and local contexts. We describe the early implementation of DREAMS in diverse settings, to identify lessons for the scale-up and replication of combination programmes for young people.

**Methods:**

As part of evaluations underway in six DREAMS sites in three countries (Kenya, South Africa and Zimbabwe), we draw on process evaluation data collected from focus group discussions, key informant interviews, and in-depth interviews with beneficiaries, parents/caregivers, programme managers and opinion leaders. Additionally, structured observations were conducted and Gantt charts completed upon consultation with implementers. We concurrently reviewed documentation available on DREAMS and held cross-site discussions to interpret findings.

**Findings:**

All sites sought to implement all components of the DREAMS core package, but how and when they were implemented varied by context. Models of delivery differed, with either multiple or single partners responsible for some or all interventions. Key challenges included the urgent and ambitious expectations of DREAMS; ‘layering’ multiple interventions across different sectors (health, education, social welfare); supporting individuals’ journeys between services to improve uptake and retention; engaging communities beyond direct beneficiaries; avoiding perceived/actual exclusivity; and ensuring continuity of commitment and funding for DREAMS. Despite significant challenges, DREAMS was well-received in the communities and perceived by both beneficiaries and implementers to empower AGYW to remain HIV negative. Structures, protocols and tools were introduced to strengthen referrals and deliver services targeted to the age and circumstances of young people.

**Conclusions:**

The benefits of combinations or integrated ‘packages’ of interventions are increasingly recognised. Early implementation of DREAMS provides useful lessons for improving coordination across multiple partners using a phased, systematic approach, regular adaptions to each unique context, and ensuring community ownership.

## Introduction

As of 2017, of the 36.9 million people living with HIV globally, 19.6 million were living in east and southern Africa [1]. Despite notable successes in prevention of mother-to-child HIV transmission (PMTCT) and early hope that widespread antiretroviral therapy (ART) would reverse epidemic trends, new HIV infections continue at an unacceptable rate. Almost 800,000 new infections occurred in east and southern Africa in 2017, close to half the global total [1]. In almost all high-prevalence countries, a group of particular concern is adolescent girls and young women (AGYW) aged 15–24 years. Incidence quickly accelerates during these ages, far faster than incidence among male counterparts [2, 3].

Women bear a disproportionate burden of infection due to underlying mechanisms spanning biological, behavioural and social factors [4,5]. These include socially-constructed gender differences between men and women, the ability to negotiate safer sex, access to resources, and gender-based violence [4–7]. Several prevention interventions have been shown to reduce risk of acquiring HIV infection by addressing single or limited determinants of acquisition [6–9]. Until ‘DREAMS’ [10, 11], there has not been a systematic investment in multiple interventions implemented synergistically at scale to reduce vulnerability to HIV.

Led by the United States (US) Office of the Global AIDS Coordinator (OGAC), and funded by the US President’s Emergency Plan for AIDS Relief (PEPFAR) and private sector partners, the DREAMS Partnership is an ambitious programme aiming to halt the persistent pattern of HIV infection among AGYW by creating opportunities for them to live Determined, Resilient, Empowered, AIDS-Free, Mentored and Safe lives (DREAMS). DREAMS provides a combination of HIV prevention packages designed to target multiple sources of risk for AGYW, e.g., the economic, social, cultural, behavioural, and biomedical factors that increase AGYW’s vulnerability to HIV infection. As described by Saul and colleagues, the core package includes interventions that aim to reduce AGYW vulnerability to HIV and enhance individual agency, with additional funding to strengthen HIV testing and treatment programmes for male sexual partners of young women [11, 12]. Crucial to the DREAMS strategy is multi-sectoral approach that creates ‘layering’ of services, for example, through referrals between or within DREAMS implementing partners [13]. Layering in DREAMS means providing multiple interventions or services from the DREAMS core package to each AGYW. The combination of interventions that should be layered depends on several factors: 1) which interventions and services are included in the country’s DREAMS programme; 2) age of the AGYW (10–14, 15–19, 20–24 years); and 3) specific circumstances of individual AGYW (e.g., experiences of sexual violence). In addition, layering includes contextual level interventions (i.e., community-based activities that are not delivered directly to an AGYW but from which she may benefit) [13]. To build on existing infrastructures, DREAMS activities are intended to be integrated within government-supported systems [12].

Evidence points to the need for combinations of interventions to tackle complex health problems such as HIV, particularly for adolescent health promotion, given that the positive development of young people spans multiple domains [6, 8–9]. Through the ‘AA-HA!’ initiative, for example, the World Health Organisation and partners promote inter-sectoral approaches for ‘global accelerated action for the health of adolescents’ [14]. The most recent edition of *Disease Control Priorities* promotes two essential and cost-efficient ‘packages’–one to be delivered during childhood and the other in adolescence–each through a mixed approach involving the community, media and health systems [15]. However, there are well-described challenges to such complex endeavours and few examples to date of success in sub-Saharan countries. Questions remain about whether and how such multi-sectoral packages can work in practice.

To answer such questions, we draw upon process evaluation activities nested within an independent impact evaluation of DREAMS in six districts in three countries (See Panel Box 1 for details of each setting):

- Kenya: one rural district in western Kenya; and two informal settlement areas of Nairobi
- South Africa: a rural district in KwaZulu-Natal
- Zimbabwe: two urban districts, focusing on young women who sell sex

The process and criteria for selecting these DREAMS sites for independent evaluation are described in the protocol for the broader impact evaluation [16].

In this paper we describe early lessons from the initial stages of DREAMS implementation, as it is being rolled out in each setting. Scaling up numerous interventions in the core package to the target population through multiple partners–unaccustomed to working together, in many cases–was expected to be challenging. In particular, the co-ordination, layering and targeting of interventions to those who need them most would be complex. Here we document how PEPFAR guidance for DREAMS is translated into practice in different social, political and epidemiological contexts. Specifically, we set out the process and timing of early implementation of DREAMS (after approximately one year of roll-out), summarise the key similarities and differences in DREAMS models, and identify challenges and successes that offer lessons for the scale-up or replication of DREAMS-like multi-sectoral approaches for HIV prevention among young women.

**Panel Box 1. Description of settings where DREAMS impact evaluation is underway**

**South Africa**

One district of KwaZulu-Natal province, which is predominantly rural with a single urban township and pockets of peri-urban settings with a high HIV prevalence. As a sparsely populated rural area, there were few targeted HIV prevention interventions for adolescents and youth prior to DREAMS. However, there were several initiatives focusing on care and implemented by community-based organisations (CBOs) that emerged from home-based palliative care in the pre-ART era to work with orphans and vulnerable children (OVC). This included social asset building interventions such as child support and financial literacy work [17], prior to DREAMS. There was also widely available school-based life orientation skills, with some peer support, freely available HIV testing and ART since 2004 leading to a decline in mortality, and, more recently, increased promotion and uptake of voluntary medical male circumcision (VMMC) [17].

**Kenya (urban)**

The two informal settlements under evaluation are situated in Makadara and Ruaraka sub-Counties of Nairobi. They are both characterised by high levels of poverty coupled with inadequate access to social and medical amenities. With previous studies [18–20] suggesting that the prevalence of HIV is higher among individuals living in urban informal settlements (‘slums’) compared to non-slum urban and rural areas, efforts from government and non-governmental sectors were directed to these areas to address HIV risk before the inception of the DREAMS Partnership. In particular, the availability of free HIV testing services provided through a number of programmes has promoted access to HIV care. In both areas, there have been ‘on and off’ HIV-related programmes targeting young people, including condom education and promotion, clinical services for HIV testing and linkage for HIV treatment services.

**Kenya (rural)**

Evaluation is underway in a rural impoverished sub-county within Siaya County–one of the four counties in Kenya carrying nearly two thirds of all new HIV new infections and the second highest HIV prevalence in Kenya [20–21]. The area under evaluation has a history of PEPFAR-funded interventions including VMMC, Fisherfolk HIV care and treatment, OVC programming, key population programmes, and HIV testing services including home-based testing, and anti-retroviral treatment roll-out [21]. Other previous prevention efforts in the area have included curriculum-based programmes, both school- and community-based. The Government of Kenya’s Ministry of Education started implementing free primary education in 2003 extending free education into secondary schools in 2008. Prior to DREAMS, the Millennium Villages project was piloted across 11 villages within this locality, seeking to improve access to healthcare, education, water resources and agricultural yields. The on-going demographic and health surveillance platform included some interventional studies targeting adolescent girls.

**Zimbabwe**

Two districts in Zimbabwe (labelled ‘Districts A and B’ for this paper), which are predominantly urban with pockets of peri-urban settings with high HIV prevalence. Both districts have few HIV prevention intervention targeting adolescent girls and young women. However, there are interventions focusing on anti-retroviral treatment and care of orphans and vulnerable children within the communities. These interventions have preceded the DREAMS programme, and include community psychosocial support programmes and in-school programmes focusing on guidance and counselling and gender norms curricula. Both districts are existing sites in the national network of ‘Sisters with a Voice’, providing clinical, social and legal support to reduce HIV acquisition among sex workers and their clients.

## Methods

### Ethics

Ethics approval was received by LSHTM (Ref 11835) and ethics committees in each host country, including the Biomedical Research Ethics Committee of the University of KwaZulu-Natal, South Africa; the Medical Research Council of Zimbabwe; AMREF and KEMRI for the research in Nairobi and Siaya, Kenya, respectively. Written informed consent was obtained from all participants.

### Data collection methods and sources

We used a rapid qualitative assessment approach [22–23] to collect data on the timing, processes, events, and experiences of beneficiaries, stakeholders involved directly or indirectly with DREAMS implementation, and evaluators of the DREAMS interventions after the first year of DREAMS roll-out. Through rapid compilation and analysis of relevant information from a range of sources and activities, we sought answers to the following questions in each DREAMS site:

- What was the process and timeline of DREAMS introduction and roll-out, *i*.*e*., what happened and when did it happen?
- How and by whom was the DREAMS core package adapted and implemented in each setting?
- How were interventions in the core package layered?
- What were the key challenges and successes in the early phases of DREAMS implementation?

These questions were informed by the UK Medical Research Council (MRC) guidance for process evaluation of complex interventions [24]. The MRC framework recognises that impact of a new intervention will be affected by three main themes—implementation, mechanisms, and context–and a clear intervention description is needed to investigate these components via process evaluation. Here, we sought to describe the intervention and its implementation, so that in-depth analyses can follow after full implementation of DREAMS and as part of the wider impact evaluation [16].

Table 1 summarises the range of methods used to gather data over a period of five months between April and August 2017, including focus group discussions, formal and informal interviews (including key informant and in-depth interviews with DREAMS implementers and clients, respectively), community mapping and structured observations. (Instruments for each activity are available in S1–S15 Files.) In addition, to construct implementation timelines in each district, we prospectively tracked roll-out of each DREAMS intervention with Gantt charts monthly from September 2016 (capturing any earlier activity retrospectively). (See S1 Table for a sample Gantt chart.) Monthly updates to the Gantt charts were completed during meetings with implementing partners. We also reviewed documentation available on DREAMS including the *Guidance for PEPFAR Country Teams on the DREAMS Partnership (2015)* [12] and country-specific guidance including tools for mapping and district prioritisation, project coordination structures, screening and referral protocols, and others specified in Table 1. Details of the specific approach to data collection in each site follow.

**Table 1:** Data sources to track DREAMS implementation in each setting.

	Process evaluation activities	Monitoring activities
**South Africa** - KwaZulu-Natal	• Semi structured in-depth interviews (n = 10 AGYW beneficiaries; n = 9 DREAMS implementing partners [IPs]) • Community discussions with mixed-gender, female- and male-only groups (n = 11) • Group discussions with learners/students (n = 2) • Stakeholder interviews with local and district municipality, government departments including health and social development (n = 9) • Community mapping in 5 communities: participant observation, e.g., ‘Let’s Talk’ and ‘Stepping Stones’, and short interviews to understand the social context for adolescents and young people and the reach of AGYW services including DREAMS interventions	• Gantt charts developed by evaluators to track the status of implementation on a monthly basis (each service in the core package) • Review of DREAMS documents: DREAMS monitoring and evaluation (M&E) Framework for South Africa • Review of DREAMS tools: mapping and district prioritisation; centralised M&E database for IP reporting (‘DIMES’)
**Kenya (urban)** - 2 informal slum settlements in Nairobi [labelled Settlements A and B]	• Key informant interviews (n = 10 with DREAMS IPs, village chiefs, youth leaders, and service providers) • In-depth interviews with AGYW (n = 20) and ABYM (n = 20) • Group discussions with parents (n = 2), DREAMS mentors and facilitators (n = 4) • Structured observations (n = 16), including DREAMS ‘safe spaces’ and health facilities	• Gantt charts (as above) • Review of DREAMS documents: Service uptake forms for all Kenya IPs (made available July 2017); DREAMS minimum package and situation-based service packages for Kenya (available Aug 2017) • Review of DREAMS tools: centralised M&E database for IP reporting of DREAMS enrolments and services provided in Kenya
**Kenya (rural)** - a sub-county of Siaya county	*N/A Not started at time of writing*	• Gantt charts (as above) • Review of DREAMS documents and tools for Kenya (as above) • Reflection sessions with programme managers of DREAMS IPs
**Zimbabwe** - 2 districts [labelled Districts A and B]	• Mapping exercise to identify hotspots where young women sell sex • Focus group discussions with AGYW (n = 2) • Informal phone interviews with key informants (n = 8 DREAMS IPs) • Key informant interviews with IPs (n = 16) • In-depth interviews with ‘seeds’ (young women who sell sex [YWSS]; n = 10)	• Gantt charts (as above) • Review of DREAMS documents: DREAMS Screening and Referral Guidelines (Aug 2017); Referral protocol (Oct 2016); national, provincial and district multi-partner meeting minutes • Review of DREAMS tools:Screening/assessment tools; DREAMS Service Passport; centralised M&E database in DHIS2 for IP reporting of DREAMS services & referrals; Ministry of Health and Community Care referral book (updated for DREAMS 2017)

#### South Africa

A mapping exercise was conducted in each of 5 communities, prior to any recruitment and interviewing of participants. This involved orienting the research team to the layout of the community and conducting short, informal interviews with community members met during the mapping exercise to identify potential participants and areas for further observations. All interviews and group discussions were conducted in the local language isiZulu by a team of nine members, five females and four males, who speak isiZulu as a first language and had worked and resided in the study area for 5–10 years. Venues for group discussions were prearranged with appropriate gatekeepers and included school and community halls. Interviews were conducted in participants’ homes or offices (for stakeholder interviews). All interview and group discussion data were audio recorded, transcribed verbatim and translated into English by the same team.

#### Kenya (urban)

Participants for in-depth interviews were purposefully selected as implementers of DREAMS or key decision-makers; AGYW were selected from the quantitative interview sample (from the impact evaluation [16]) as having received one or more DREAMS interventions, and adolescent boys and young men (ABYM) had either participated or were aware of the DREAMS programme. Four field interviewers, 2 males and 2 females, were recruited based on their academic qualifications, previous experience in qualitative data collection in this setting, and fluency in English and Kiswahili. The evaluation team in Nairobi also participated in the collection and transcription of recorded interviews. Interviews were conducted in conveniently located areas within the slums so participants could easily attend. These included ‘safe spaces’ where DREAMS activities take place, such as church and community halls for the qualitative cohort in-depth interviews and focus group discussions. Interviews with the DREAMS IPs, village chiefs, youth leaders and service providers took place privately in their offices.

#### Zimbabwe

The data collection team comprised 8 female social scientists, each with more than 5 years research experience. Interviews were conducted in the local languages, Shona and Ndebele, and English. Community mapping was done to gather information on different kinds of sex work available, how the social networks of sex workers are organised, and to assess the feasibility of impact evaluation methods for the target population of the Zimbabwe impact evaluation: young women who sell sex (YWSS) [25]. Informal focus group discussions were conducted with a group of 8–12 peer educators and with different types of sex workers (street based, venue based, and social/demographic stratifications) selected during the community mapping. Observers’ notes were written during the discussions. Informal phone interviews were done with 8 implementing partners and notes recorded by hand. In-depth interviews were guided by qualitative topic guides, and all were audio recorded, translated and transcribed by the social scientists.

### Data summary and thematic analysis

Guided by the main research questions above, analyses were led by researchers at each site and discussed in regular debriefings with data collection teams. Data familiarisation was followed by manual coding and summarising in a data extraction sheet (S2 Table) and then synthesis by emerging themes. Insights from team debriefings and interviewers’ field notes were used to refine and add codes and themes, as new transcripts became available, to promote participatory analysis by site teams.

A cross-site working group, including representatives from each evaluation setting, held monthly teleconference meetings to share findings across the settings and organise data into templates. Through ongoing, participatory analysis by the working group, themes emerged about the timeline, differences and similarities in DREAMS roll-out, and lessons about challenges and opportunities related to the scale-up of DREAMS and other multi-sectoral programmes. Further, a writing workshop was held with researchers from all sites to discuss and distil the emerging findings within and across contexts.

### Findings

In all six sites, efforts were made to provide all elements of the DREAMS core package, but how and when DREAMS was implemented differed by context. In general, we observed five phases (not necessarily planned) in the roll-out of DREAMS, with each site moving through an extended period of **preparation** and planning, before an early, **staggered roll-out** of services that preceded **scale-up** of services to reach pre-defined targets. In all sites, **‘programme adjustment’** and planning for **continuation** of DREAMS began in the second year (2017). We describe below how the timing and nature of these five phases differed in each site, and what was learned at each stage. Table 2 summarises key events in the introduction and roll-out of DREAMS in each setting and S3 Table compiles key characteristics of programme implementation across the sites.

**Table 2:** Key events in the introduction and roll-out of DREAMS.

Key events / milestones in the roll-out of DREAMS	South Africa (KZN)	Kenya urban		Kenya rural	Zimbabwe	
		Settlement A	Settlement B		District A	District B
Announcement of the DREAMS Partnership	1 Dec 2014 (World AIDS Day)					
Selection of 10 DREAMS countries	1 Dec 2014					
Country proposals approved by US Govt	July 2015					
Proposed timeline for DREAMS delivery	Oct 2015 –Sept 2017					
Recruitment of first DREAMS clients / beneficiaries	From April 2016 (via mapping / geographical prioritisation of vulnerable areas)	Jan–July 2016 (via Girl Roster plus other methods)	Feb–Oct 2016 (via Girl Roster plus other methods)	From Feb 2016 (via Girl Roster plus other methods)	From end of 2015	From Jan 2016
First DREAMS services provided to DREAMS clients	May 2016	Jan 2016	Feb 2016	April 2016	Feb 2016	
All interventions in core package available (by month & year, excluding PrEP)	Nov 2016	Jan 2016	Feb 2016	Jan 2017	Feb 2017	
Specific guidance / tools for referrals introduced	Layering Guidance, July 2017	Pre-existing: MOH referral protocol & tools End of 2016: standardised format for DREAMS IP reporting			Nov 2016: Referral protocol Aug 2017: Screening & referral guidelines	
‘Primary’ package specified	July 2017	July 2017			May 2017	
First targets met	April 2016	Oct 2016: Yr 1 overall target met July 2017: Yr 2 target surpassed	Sept 2016: Yr 1 overall target met July 2017: Yr 2 target surpassed	Sept 2016	March 2017	March 2017

### 1. Preparation phase

DREAMS was planned to begin in October 2015 and continue over two years through September 2017. The start date was not feasible in any of the six settings, as preparation for DREAMS proved time-consuming and often challenging.

Partnerships were established between the United States (US) and host country governments at various levels. In South Africa, for example, there were multiple government departments involved, including the Department of Health (DoH), Social Development (DSD), and Basic Education (DBE). Municipalities as well the AIDS councils were involved at district, local municipality and ward levels—District AIDS Council (DAC), Local AIDS Council (LACs) and Ward AIDS Council (WACs); Cooperative Governance and Traditional Affairs (COGTA); civil society and DREAMS Implementing Partners (IPs) and PEPFAR liaisons. These formed the district project implementation team responsible for implementing DREAMS and ensuring the alignment of DREAMS activities with existing programmes. Similarly, in Zimbabwe partnerships were established between the US and Zimbabwe’s government, including national and district levels. Ministry of Health and Child Care, Ministry of Primary and Secondary Education, Ministry of labour and Social Services and National AIDS Council were involved in implementation at both national and district levels.

In all countries, identification of sub-national units (SNUs)—or geographic areas of priority for DREAMS investment–was typically based on HIV prevalence and incidence criteria, but other factors influenced the selection, like the number of adolescents and young adults, teenage pregnancies and saturation of ART and VMMC via previous US Government programmes. Within Kenya, four counties (three rural and one urban) with a high burden of HIV were selected by the Ministry of Health as priority areas for implementing DREAMS. In South Africa, once provinces were agreed, site selection was based on a detailed mapping exercise conducted with numerous stakeholders to identify geographic priorities to the ward level. This was time- and resource-consuming, requiring many meetings, but considered, by stakeholder informants, to be beneficial: it strengthened participation and buy-in at provincial and district levels from early stages; introduced stakeholders; and mapped the area in which partners were to work.

In terms of who would deliver the Core Package of interventions, three distinct models were observed across the six sites:

1. Multiple implementing partners (IPs) were contracted by a US Government agency (e.g., the Centers for Disease Control (CDC), US Agency for International Development (USAID), Department of Defense, or Peace Corps), with each IP delivering different interventions of the package in the same area based on their expertise. This model was adopted in South Africa and Zimbabwe, with some IPs further sub-contracting to community-based organisations (CBOs).
2. Two IPs working in the same area, each with distinct remits. This model was used in the rural setting in Kenya, where one IP was contracted to deliver all interventions intended for adolescent girls (10–14 years) and their families; and another IP responsible for young women (15–24 years), their partners and families.
3. One IP per area. In the urban informal settlements, one IP was contracted to coordinate the delivery of all interventions to all target groups in the designated area.

The selection of Implementing Partners (IP) was usually competitive, utilising a bidding process, and the time needed for contracts and disbursement of funds varied considerably by site. Time was also needed to agree the respective roles of multiple IPs, particularly organisations with less prior history of working in the DREAMS site and how they would work together with organisations with a long-standing presence. Further site-specific details about the delivery models are provided in Panel Box 2.

**Panel Box 2. The models used to deliver the DREAMS core package in each setting**

**South Africa**

In the evaluation site in KZN, South Africa, five main implementing partners (who subsequently sub-contracted five CBOs to deliver components of the core package) were contracted to work in the same DREAMS site. Each IP delivers interventions that match their expertise, and makes referrals for services they are not contracted to deliver. Three of the five implementers were not new to the district, and most continued providing activities they were delivering before DREAMS, adding teams to deliver new interventions through their DREAMS contract. A District Support Partner from one of the IPs was appointed to coordinate activities across the multiple IPs.

**Kenya**

In rural Kenya, implementation of DREAMS was rolled out by two implementing partners that were solely responsible for the implementation of all DREAMS interventions but with different target groups: one focused on 10–14 year olds and another on 15–24 year olds within the same geographic area.

In the two urban informal settlement areas, one sole IP was contracted in each area to coordinate delivery of all interventions in the DREAMS core package. The selected IPs were organizations that had experience in running various programmes offering HIV related services and are well-known within their respective communities, but required training in some components of the core package for which they had no previous experience.

**Zimbabwe**

In Zimbabwe, six partners implemented DREAMS supported by sub-partners in each district. The IPs subcontracted at least one or more community-based organisations. Most IPs had been operating in the districts for many years, delivering the same services that they were contracted for DREAMS, so they were well known by beneficiaries although not accustomed to working together. One IP was given responsibility for coordination in each area; in one area the ‘lead IP’ was particularly proactive, and considered a key ingredient for successful implementation.

Another variation was in the approach used to identify target groups for DREAMS interventions. In both Kenyan sites, the Girl Roster–a census method to identify the universe of girls in the ‘walkable community’–took several months to conduct [26]. In each case, the Girl Roster identified more potential beneficiaries than resources/quotas would allow, and was supplemented with other methods to identify the highest-risk young women (e.g., consultations with community- and faith-based organisations with experience in the community). In Zimbabwe, the Government’s cash transfer registry was used as the initial framework (and denominator) for identification of DREAMS beneficiaries. Again, a secondary screening process was needed to recruit AGYW because the registry did not exist in urban settings and the vulnerability criteria for cash transfers did not completely overlap with those for DREAMS. In the two Zimbabwean districts in this evaluation, a network-based recruitment approach (respondent driven sampling) was used to identify young women who sell sex and link them into DREAMS programmes (described in greater detail elsewhere) [25]. In South Africa, the geographic mapping exercise, described above, identified priority areas within which all AGYW were potentially eligible and the IPs and in particular the community-based organisations with a presence in the area identified potential beneficiaries within their targets/quotas.

### 2. Initial roll-out

Table 2 shows that, across the sites, the first DREAMS-funded services began between January and May 2016. Roll-out of services in the Core Package was staggered and *ad hoc*, with services that had a pre-existing infrastructure being the first to come online through DREAMS, e.g., existing HIV testing services were expanded to reach more AGYW and male partners. New interventions took longer to introduce, especially social asset building and social norms programmes, with most IPs needing time for training (e.g., in *Safe Spaces* and *SASA*! programming) and adapting the new interventions to their setting (e.g., how, where, when, by whom they would be delivered). Some interventions were integrated with government services, e.g., the cash transfer and educational subsidy programmes in Kenya and Zimbabwe, and took considerable time to align, to avoid duplication of beneficiaries. Most sites were delivering most interventions in the Core Package by the end of Year 1. In each site, oral PrEP was one of the last services to be offered via DREAMS.

The reception of DREAMS by communities was generally positive, and particularly welcomed by the beneficiaries. The tangible benefits of some interventions were cited, in group and individual interviews, as an advantage of DREAMS over other, previous programmes, and helped to overcome some initial skepticism. Further, there was a popular belief that DREAMS would indeed protect AGYW and reduce HIV risk, through the combination of support services to help AGYW remain HIV-negative. In South Africa, girls who had tested for HIV and/or been part of other DREAMS interventions were happy to be involved and, among those who tested negative, there was often a positive attitude about wanting to remain negative. Similarly, in Kenya and Zimbabwe, communities felt that continuity of DREAMS interventions would ensure that AGYW would be at less risk of contracting HIV because of the behaviour change resulting from their participation. DREAMS mentors in the Kenyan urban sites expressed optimism about the programme’s ability to empower young women to avoid HIV, citing the emphasis on self-reliance and personal development.

However, in all sites, there were concerns about those perceived to be excluded by the programme, which led to hostility and tensions in two settings. A common question voiced in interviews and group discussions was ‘What about the boys?’ Adolescent boys and young men were perceived to be at high risk (particularly for social risks like alcohol use, unemployment, poverty), and numerous interview participants expressed a concern that DREAMS’ prioritization of girls would be at the expense of young men. This led one Implementing Partner to encourage sharing of tangible benefits, like solar lamps for homework, with boys in the same household. In South Africa and Zimbabwe, PrEP was also offered to men in an effort to include them both generally and with a view to capturing potential male sex partners. Other IPs in South Africa included boys in their parenting programmes using their own resources in addition to the DREAMS programmes that already included boys (*ASPIRES* and *Stepping Stones*) as a way to include more boys in DREAMS interventions.

In some cases–particularly in high-density urban areas in Kenya and Zimbabwe where poverty and unemployment are high–some IPs faced harassment and interruptions from individuals or groups that were not receiving DREAMS interventions, particularly the cash benefits. Some IP staff members were the victims of petty crime, and local ‘bodyguards’ were hired to facilitate their work.

### 3. Scale-up

Across all sites, for all implementing partners, there were set targets and indicators for the number of beneficiaries to be reached over the two-year DREAMS implementation period. The targets were used as a guide to track delivery of services during the implementation process, and ensure adequate reach. However, targets led some IPs to feel pressured to meet quotas even at the expense of the quality or suitability of interventions delivered. In some cases, targets were also perceived to impede the layering and referral of services as IPs ‘chased’ their own targets, rather than refer to other IPs. The first targets for DREAMS core package were met between April 2016 and March 2017 (Table 2) showing the different rates at which ‘scale-up’ occurred across sites and for different interventions. IPs felt that some targets were more challenging to attain. For example, targets for HIV testing were often exceeded, unlike coverage by curriculum-based programmes. AGYW clients were expected to attend at least 80 percent (%) of sessions of curriculum-based interventions for IPs to report the outcome as ‘achieved’–this was difficult for programmes that relied on sustained engagement, e.g., participation in 10–12 sessions (e.g., *Stepping Stones* or *ASPIRES*). Further, some programs were less able to engage older age groups–young women aged 20–24 years–(e.g., *Safe Spaces*) yet IPs were still expected to achieve and report on these. Problems with commitment and availability of women in these age groups were highlighted as the main challenge. The older AGYW– 20–24 year olds–had competing demands on their time by children, partners and seeking/employment, as well as some hostility from male partners that in at least one case put a young woman at risk of harm. In some cases, IPs altered the times at which programmes were made available, e.g., to evening / weekend time-slots, or reduced the frequency of the sessions (from weekly to biweekly), to help retain young women.

The coordination of layered interventions has been an iterative process with some site-specific solutions emerging along the way. Scale-up of DREAMS occurred quickly and there were tensions between approaches that are embedded in communities and thus adaptable, and needing to layer through a multi-sectoral approach. Layering was quickly recognised as a key challenge and several meetings were held with IPs and steering teams in South Africa to improve the mapping tool and ensure coordination of services. In South Africa and Zimbabwe, having multiple implementing partners in the same district posed difficulties with the tracking of referrals to facilitate layering. However, some IPs sub-contracted CBOs who knew the area and communities well and were able to facilitate better referral and layering of activities.

### 4. Programme adjustment in the second year

Early indications from United States Government-funded monitoring and evaluation (M&E) data in Kenya and Zimbabwe identified two shortcomings of DREAMS implementation in its first year: 1) layering was not being achieved as hoped for, and few AGYW were receiving multiple services through DREAMS (and/or reporting of layering needed improvement); and 2) DREAMS was not always reaching the highest risk AGYW. Efforts were made to strengthen these two aspects in Year 2.

In the second year, OGAC issued DREAMS Layering Guidance [13] to all countries, to define layering–a ‘fundamental principle’ of DREAMS–and improve its delivery and tracking. The strategies employed by countries to improve layering depended on the model for delivering DREAMS, and existing systems for integration. For example, in Zimbabwe, where multiple Implementing Partners worked in the same areas, screening and referral protocols (based on Ministry of Health and Community Care tools) were strengthened in Year 2 to improve linkages between all DREAMS IPs, e.g., in making (issuing) and completing (receiving) referrals of DREAMS clients. Specifically, IPs were expected to complete at least 80% of their DREAMS referrals. In Kenya, where a single IP delivered DREAMS, efforts were made to sustain AGYW participation, e.g., through allocation of a mentor and a DREAMS badge for every girl enrolled. In all countries, a minimum or ‘primary package’ of DREAMS services was defined by age group and sub-population of AGYW, for clarity about which services need to be layered per age group and need. The primary packages specified combinations of services spanning biomedical, behavioural and structural (social asset) interventions. In South Africa, where layering was particularly challenged by having multiple IPs and the absence of a shared identification code, a district coordinator was introduced to support IPs with coordination, layering and other challenges with implementation.

To improve the measurement and tracking of layering, refinements were made to country-level ‘M&E’ databases used to consolidate routine/programme data reporting by Implementing Partners. By Year 2, Kenya and Zimbabwe had adopted a unique ID for each DREAMS client, so that M&E databases were able to avoid double counting and track service provision at the individual level. Each country developed a different database, e.g., an adaptation for DREAMS of the Demographic Health Information System (DHIS2) in Zimbabwe; a variation of DATIM in South Africa (called ‘DIMES’), and a bespoke database created by CDC/University of California San Francisco (UCSF) in Kenya. These databases are not easily compared across countries, e.g., they often define and count services differently. In South Africa, the database did not capture referrals and therefore could not be used to show degree of layering between partners or the number of services AGYW had received which was a missed opportunity to better understand risk and vulnerability. In Zimbabwe, plans were made to further modify DHIS2 in Year 3, to track the primary packages by age and circumstance of AGYW clients.

Some of the above efforts also helped to address the concern that DREAMS was not reaching the most vulnerable AGYW. For example, screening protocols and tools helped to identify potential DREAMS clients, for referral into relevant services. Also, the primary packages helped by defining services for sub-populations, including key populations (young women who sell sex in Zimbabwe) and clients who experienced sexual violence.

### 5. Continuation of DREAMS

To continue beyond the initial timeframe of 2 years (post Sept 2017), the DREAMS programme was absorbed into the US Government’s Country Operational Plans in each country. Prior to this, IPs were uncertain of their own funding beyond Year 2, and sometimes reluctant to raise concerns and hinder their chances of further funding (e.g., when they felt the quotas were inflexible). This may have led to missed opportunities to improve the programme, in response to IP insights. Certain that services could not continue without funding, e.g., through community volunteering, IPs also expressed worry that DREAMS would grind to a halt very soon after starting in earnest. Continuation can help to ensure that the DREAMS core package is implemented, and evaluated, at the scale and duration intended. The funding levels for DREAMS in years 1–3, as presented by the Office of the Global AIDS Coordinator, are presented in S1 Fig. They represent total investments for all DREAMS districts within a country, and not just the sites included in this evaluation.

## Lessons learned: Conclusions and the way forward

As the value of combinations or ‘packages’ of services is increasingly recognised–for HIV prevention and health promotion more broadly–DREAMS provides lesson for co-ordinated and targeted layering of interventions across health, education and social welfare sectors. In Table 3, we have summarised the challenges and opportunities that DREAMS’ early implementation created across the sites as lessons for the continued scale-up of DREAMS and of other multi-sectoral programmes.

**Table 3:** Summary of challenges and opportunities for multi-sectoral programming.

Challenges	Opportunities
DREAMS was ‘a big lift’–requiring a huge effort to get it off the ground	This has mobilised multiple sectors, ministries, and organisations to work together. DREAMS was generally well received and highlighted AGYW as a priority group (although there were concerns about those perceived to be excluded, especially boys and young men)
Expectations are ambitious and bold to implement and achieve impact in a quick timeframe	This created a momentum and urgency to find solutions to challenges and make DREAMS happen. The shared commitment fostered collaboration.
Coordinating multiple components of the DREAMS Core Package—at institutional level was challenging	New structures and strategies were used to coordinate multiple implementers and interventions; these can be strengthened and sustained for multi-sectoral collaboration and better communications going forward
A ‘new way of working’ was difficult given lack of existing systems, structures or incentives for organisations to link their services for AGYW	
Delivering all interventions in the Core Package in one geographic area was untenable in the time allocated	DREAMS led to the expansion of existing HIV services and strengthened health system delivery
	Creation of new programmes, including the introduction or expansion of PrEP availability, and improved human resource capacity for interventions promoting social norms, social assets and structural drivers. In some cases, this created new HIV prevention services where few existed before.
	Creative solutions emerged to adapt the PEPFAR guidance to each context. Further analysis can explore whether this strengthens or hinders the impact of DREAMS.
Layering services in the DREAMS Core Package–at individual AGYW level	Better integration of services–with tested models that can be applied to other population groups (beyond AGYW) and services (beyond HIV prevention).
	Strengthened screening and referral protocols; formalised linkages between organisations; use of passports, and badges were innovations that emerged from the opportunities DREAMS presented
	Recognition of high-risk populations (the highest risk), and appreciation for the unique and comprehensive needs of AGYW.
Tracking the layering of services	The use of a unique ID has strengthened information systems to monitor DREAMS services, but could be improved to track layering and primary packages, and services by individual risk profiles, e.g., to gauge whether higher risk AGYW and male partners are reached, and ‘elite capture’ can be avoided.

Across all sites, DREAMS was generally well received by beneficiaries, communities and various stakeholders, although, in some cases, concerns about the exclusion of boys and young men created tension and resentment. The DREAMS goals were ambitious in a short timeframe but the commitment and buy-in at all levels was high, including by national governments. Given the complexity of rapidly scaling up this innovative multi-sectoral package, a phased roll-out may have allowed a co-ordination mechanism to evolve. Some sectors had no history of working together yet were expected to co-ordinate the delivery of targeted and layered interventions, effectively and rapidly.

Implementation of DREAMS was not intended to be phased or staggered but to roll-out at the same time across the sites. In practice, however, some ‘unplanned’ phasing of implementation of DREAMS activities occurred in all sites. With the rapid timeline planned for DREAMS, each site worked with existing services, partners, and systems wherever possible–while adding and adapting new interventions–and testing new models and ways of working. Implementing partners felt that given more time and planning, and clear coordination strategies, layering could have been possible sooner. This could also have broadened the focus from attaining targets to delivering integrated services across partners.

The ambition and complexity of DREAMS created many challenges for all involved in its roll-out. However, the urgency, expectation and financial investment generated momentum and commitment to making DREAMS happen. Challenges had to be overcome and in the process, they created opportunities for continued, strengthened multi-sectoral programming–particularly for the benefit of adolescent girls and young women.

Some elements of DREAMS were easier and faster to scale up, particularly those that could rely on existing infrastructure and delivery models, for example HIV testing services. Others took time to take off, especially the interventions addressing social norms and violence prevention among the broader community, and curriculum-based programmes that required training of IP teams and sustained commitment of clients. DREAMS has brought in new programmes or expanded the availability of services that were not initially available in communities, including PreP. This has led to the creation of sexual health programmes for key populations in communities where they were not available, and the general population can benefit from the broader community-based programmes.

Given the scale of problems facing AGYW, there is an urgent need to consider the context in which interventions are delivered to inform scale-up of evidence-based combination HIV prevention [24, 27–28]. By documenting early implementation in six diverse evaluation sites, we saw that various models of delivering the DREAMS core package emerged across sites—each with its own advantages and disadvantages based on the contexts. There is need in the future to allow time to foster stakeholder and community engagements at local levels to ensure early ownership of programmes and contextual adaptation by implementers both established and new.

## Supporting information

S1 File DREAMS Impact Evaluation, Process Evaluation Tools, Kenya (English). (DOCX)

S2 File DREAMS Impact Evaluation, Process Evaluation Tools, Kenya (Swahili). (DOCX)

S3 File DREAMS Impact Evaluation, Focus Group Discussion Guide, South Africa (English). (DOC)

S4 File DREAMS Impact Evaluation, Focus Group Discussion Guide, South Africa (Zulu). (DOC)

S5 File DREAMS Impact Evaluation, Key Informant Interview Guide, South Africa. (DOCX)

S6 File DREAMS Impact Evaluation, In-depth Interview guide for qualitative cohort, South Africa (English). (DOC)

S7 File DREAMS Impact Evaluation, In-depth Interview guide for qualitative cohort, South Africa (Zulu). (DOC)

S8 File DREAMS Impact Evaluation, Semi-structured Key In-depth Interview Guide, South Africa (English). (DOCX)

S9 File DREAMS Impact Evaluation, Semi-structured Key In-depth Interview Guide, South Africa (Zulu). (DOC)

S10 File DREAMS Impact Evaluation, Structured Observation Guide, South Africa (English). (DOCX)

S11 File DREAMS Impact Evaluation, Structured Observation Guide, South Africa (Zulu). (DOC)

S12 File DREAMS Impact Evaluation, Guide for Transect Spiral Walk, South Africa. (DOC)

S13 File Community Mapping Guide for Formative Research in Zimbabwe. (DOC)

S14 File DREAMS Mapping Topic Guide, Zimbabwe. (DOCX)

S15 File Cross sectional qualitative interview guide (6-monthly), Zimbabwe. (DOC)

S1 Table Timeline of implementation of DREAMS in one impact evaluation setting (Site X). (XLSX)

S2 Table Data extraction sheet. (XLSX)

S3 Table Characteristics of the implementation process across DREAMS evaluation sites. (DOCX)

S1 Fig PEPFAR DREAMS: “The commitment remains both in funding and in focus”. (PDF)
