# Supplementary material for: Translating DREAMS into practice: Early lessons from implementation in six settings
Source: PLoS One. 2018 Dec 13;13(12):e0208243. doi: 10.1371/journal.pone.0208243 (PMC6292585; doi:10.1371/journal.pone.0208243)
Supplement: S1 File — (DOCX) [file pone.0208243.s001.docx]

**S1 File. DREAMS Impact Evaluation, Process Evaluation Tools, Kenya (English)**

1. [IDI Guide – Qualitative Cohort](#_Qualitative_longitudinal_study)
2. [IDI Guide – Key Informant](#_Key_informant_interviews)
3. [FGD Guide – Parents](#_Focus_Group_Discussion)
4. [FGD – Mentors/facilitators](#_Focus_Group_Discussions)
5. [Observation Form](#_Structured_Observation_Guide_1)

**DREAMS IMPACT EVALUATION STUDY**

**Kenya**

# **Qualitative longitudinal study with young people - in-depth interview guide for young men and women**

This is an in-depth interview and below is the topic guide with some open-ended questions that you can use. But the key is to probe and gain depth of information around the young person’s experiences and expectations about DREAMS.

Complete the coversheet provided in Annex 1

**Questions/Themes**

**Personal**

1. I would like to begin by knowing more about you. What is important to you, how and where do you spend your time, what do you enjoy or don’t enjoy, who do you socialise with etc? [*As a way to establish rapport and get to know the participant Let the participant tell you anything about themselves, about their family and any other general information]*
2. How long have you lived in this community?

***The next three questions may be personal, emotive questions (parents death, and reasons for dropping out of school), assure the respondent as needed***

1. What is your level of education? Probe - How far did you reach in completing your studies if you are no longer in school? What are the reasons for not being in school if you are supposed to be in school? If in school, ask what grade they are in.
2. If below 18 years, ask if their parents are alive? Probe who they live with and who is taking care of them. Also ask how many children are in their household and who is providing for the household financially?
3. In the last week, have there been times when you were hungry but could not eat as there wasn’t any food? Was this the same for all people in your household or were some people more affected than others? Why? What do you usually do when this situation arises?

**Experiences with the wider DREAMS and DREAMS type interventions**

1. Have you heard of a program called DREAMS? (probe using the names of the organisations that are working in the area LVCT Health in Korogocho and Hope Worldwide in Viwandani) How did you hear of the program called ‘DREAMS’?
2. How would you describe DREAMS to someone who has never heard of it before?
3. How did you come to be involved with DREAMS? Please tell me about the first time you met anyone from DREAMS or participated in a DREAMS activity.
4. What were your expectations when you first heard about DREAMS?
5. I would like to know more about your experience of DREAMS from the beginning of your involvement until now. As I shared earlier, this interview is totally confidential, and will not be shared with the people you know at DREAMS. So please feel free in telling me about how you feel about DREAMS activities. Most people like some things more than others – Which DREAMS activities have you been involved with? What have you enjoyed most and why? Which parts do you like less and why? What would you change about DREAMS if you could? Is DREAMS better/worse or as you expected? What makes you say this?
6. Please tell me about your DREAMS mentor. What was your first meeting like? How does she help you? What do you like about your relationship with her? What do you not like about your relationship with her? Is there something that you wish your mentor could do to help and support you that she is currently not doing?
7. Are you participating in [name of DREAMS type intervention]?
   1. Which organization is providing the intervention?
   2. When did you start participating in the activity?
   3. How often did you participate?
   4. Are you still participating in the activity?
   5. What do you like about the activity?
   6. What things could be done better?
   7. What things would you like but are not available?
   8. Have you told anyone that you are involved with DREAMS? Whom did you tell? What did they think of your participation?
   9. Have you ever hidden your participation in DREAMS from anyone? Who? Why? What do you think would happen if they found out?
   10. Are your parents/guardians/caregiver involved in these activities? Probe for reasons why/why are they not involved– is it because of the sensitivity eg condom use or contraception? Explain if this is affecting/will affect continued access/utilisation?

8 . Were you ever referred from one place where you received a service that is part of DREAMS to another one e.g. from HIV testing centre to a centre that offers services for preventing mother to child transmission of HIV? How did it feel to be referred? Were you pleased by this? Did you go to the place that they referred you? When? Did you go by yourself or did you go with someone?

| ***DREAMS project interventions:***   1. Social Assets Building: Structured small groups led by mentors to build the knowledge, skills and networks. 2. HIV Testing Service: Combination of services that include counseling, HIV testing, condom distribution, risk assessment and risk reduction counselling 3. Evidence based behavioral interventions: Healthy Choices for a Better Future – targeting 10-14 years old, My Health My Choice targeting 13-17 years old and Families Matter! Program targeting caregivers of 9-12 years old 4. Education support: Targeting mainly transitional AGYW and those who dropped out of school and wish to continue. Transition from primary to secondary 5. Long term contraceptive mixed methods provided mainly through partnerships and referral. This also includes family planning education. 6. STI screening and treatment. Mainly integrated with other interventions like breast cancer screening, HTS, TB screening and family planning 7. Condom distribution and education 8. Male Sexual Partner Characterization: Profiling the men who are likely sexual partners to the AGYW so as to target them with appropriate intervention (s). It’s done by the mentors in the safe spaces 9. Vocational training and apprenticeship skills: AGYW are supported in building skills that will help them earn money 10. Entrepreneurship training to equip the girls with business skills and encourage savings through table banking 11. Post violence care. Services provided on site include psychosocial support and services through referral include but not limited to legal support, medical attention 12. Community mobilization and norm change interventions that aim to change the societal norms around women. Key area of focus is on gender based violence |
| --- |

1. Since DREAMS started, have you noticed any changes in your local community, among your friends and peers, or for yourself? ? What kinds of changes have you seen, if any what positive changes have you seen? What negative changes have you seen?

**Experience and expectation of reproductive and sexual health information and services**

1. Have you used any healthcare facility over the past year? Which one did you use? What has been your experience? What were the good things? What were the bad things? What could be better?
2. Where/from whom do young people your age in this community receive information about sexual relationships and reproductive health?
3. Have you ever received information about sexual relationships and reproductive health? What do you feel about the way in which you were taught about these topics? What did you like? What did you not like? [probe about the adequacy of the information received]. Do you wish you had more access to information about sexual relationships and reproductive health or is the information you have access to enough?
4. **[for females aged 15 and older]** Where do women in this community go for contraceptive or family planning services? Have you ever used these services?
   1. [IF YES]What was your experience using the services?
   2. [IF NO] Do you know where to go for these services? Would you feel comfortable asking for contraception? Why? Why not?
5. **[for males aged 15 and older]** Have you ever heard of voluntary male medical circumcision (VMMC)? Where did you hear about it? Do you personally know of anyone who has had VMMC? Would you consider it? Why/ why not? Have you undergone VMMC? What was your experience?

**Experience and expectation of HIV testing**

1. Have you ever tested for HIV?
   1. **[IF YES]** Where did you get tested? How long ago did you get tested? Did you get your results? What was your experience? Were you referred by someone or did you just go? Did you go by yourself or did someone else go with you? Did they also test? What could be done better? Would you recommend that setting? Why/Why not?

**Do not ask for the participant’s HIV test results,** but if they volunteer that they are positive, probe their experience of HIV care

- 1. **[IF NO]** Is there anything that has prevented you from being tested? What would make it easier for you to test? Do you know where to get a test? Would you feel comfortable going there for the test? Why/ why not?

**General observations on DREAMS related activities/issues**

1. **What does the word ‘empowered’ mean to you?**

**a.** Do you feel empowered? Why or why not? (If yes, in what way /how do you feel empowered?) What would be necessary for you to feel empowered in this community?

b. Do you feel hopeful about your future? If yes, what makes you hopeful about your future? If not, why not? What are your main concerns about your future/what factors do you think might prevent you from meeting your hopes for your future? What do you think would be necessary for you to feel more hopefully about your future?

1. **(If DREAM girl )** What kind of knowledge have you acquired since becoming a DREAMS girl? Can you describe your experience before and after being on DREAMS? How/Do you feel you are now more capable/or not of making decisions about your health (do not probe unless its not clear) eg engaging in safer sexual practises, condom use, getting an HIV test, delaying getting pregnant; education eg finishing school, doing well in school, proceeding to a higher level grade etc and socio-economic planning eg saving money or just knowing how to save money, etc (whether or not because of DREAMS)? Have you put what you have learnt from DREAMS in action – can you tell me about it? If not – what has stopped you?
2. **(If ‘DREAM boy’/ABYM involved with DREAMS)** Is DREAMS engaging ABYM? **[If yes]** How is DREAMS engaging ABYM? How has DREAMS impacted on ABYM? Do you feel it is a success? If yes, how and why?

**[If no]** Is it important for DREAMS to engage ABYM? Why? How should they be engaged?

1. What challenges do AGYWs face in this community in their daily lives? How do they overcome these challenges?
2. When going about your normal activities, do you feel safe in your community? Why/why not? What risks do you face in your community – please think about things like social risks, physical risks, health risks and risks related to your community.
   1. What factors lead to this risk (probe eg gender, age, poverty, class, ethnicity?)
   2. What factors help you to reduce these risks (probe eg family, friends, formal community structures eg police, chief etc, various organisations, DREAMS, faith/religion?
3. Has DREAMS’ changed the social behaviour (e.g attitudes towards/interaction with friends, peers, relationships, other community members e.t.c) of AGYW? If yes, what are they doing differently?
4. Is it okay for some programs to focus on AGYW to better their lives? Why? Why not?

**[For males and females 15 years and above]**

1. Thinking about ways to prevent HIV infection, what types of strategies/interventions/services are mostly used by AGYW in this community? Why? What about ABYM? Why? Where do they access/ who provides these services? How effective do you think have the strategies been in prevention of HIV among AGYW/ABYM? Why?
2. Which are the preferred health related service providers/facilities among AGYW? Why? What about ABYM? Why? Do the preferences affect their health seeking behaviour for HIV related services? Does it affect their health seeking behaviour for any other health related issues?

**Closing**

We have come to the end of the interview. Before we finish, is there anything else that you would like to add about what we have discussed?

Do you have any other feedback about the program? What other information would you like to know about the program?

**Annex 1: Interview Cover Sheet**

Recorder Number: ___________________________

File Number:__________________________

**Participant details:**

Name of Interviewer(s): ____________________________

Date: ______________

Location [informal settlement]: ­­­­­­­­­­______________________

Interviewee’s Name: _____________________

Sex [circle one]: FEMALE MALE

Marital status [circle one]: NEVER MARRIED MARRIED WIDOWED SEPARATED/DIVORCED

Schooling status: IN SCHOOL OUT-OF-SCHOOL

Highest level of education: NO SCHOOLING PRIMARY SECONDARY HIGHER

Age: ____________

**DREAMS IMPACT EVALUATION STUDY**

**Kenya**

# **Key informant interviews**

The questions below are a guide. Use the interview as an opportunity to explore any particular issues or events that may affect the implementation of the DREAMS intervention, that may be barriers and facilitators to implementation, or that capture staff members’ and beneficiaries’ perceptions and value of the intervention.

Complete the cover sheet before beginning the interview (Annex 1)

**Questions/Themes**

**DREAMS service implementers**

1. How would you describe DREAMS to someone who had never heard about it?
2. What DREAMS-related activities is your organisation involved in? What is your specific role on the project?
3. What activities did your organisation conduct before DREAMS? Who were your beneficiaries? How were you funded?
4. What were you expectations of DREAMS before you started?
5. When did the roll-out of the DREAMS activities start?
6. What preparation work was done before the roll-out?
7. Please tell me about any new or additional work that you/your organisation does since becoming involved with DREAMS.
8. What proportion of your time do you spend on DREAMS-related and non DREAMS-related work?
9. Did you experience any challenges as you were rolling out the program? If Yes, please tell me about them
10. I understand your organisation is working in several villages. Please tell me about your experience of working in these villages. Do you feel that it has been easier to implement your work in some of these areas compared to others? Why do you think this may be?
11. The DREAMS initiative comprises different interventions. Can you tell me about your experiences implementing these components? Do you feel that it has been easier to implement your work in some of these areas compared to others? Why?
12. In your view, how is DREAMS experienced by those who deliver it and wider community members?
13. How do beneficiaries perceive and value the various DREAMS interventions? What do they think of the quality of the interventions delivered?
14. What does success in implementing DREAMS look like? What kinds of things are helping you to move in that direction, and what kinds of things get in the way?
15. How does the reality of DREAMS compare to your expectations when you were first starting out?
16. Have you made any adaptations or changes to the DREAMS interventions so that it better suits your organisation or beneficiaries? Please tell me about any innovations or changes that you have made to the intervention.
17. In your view, have there been any changes in the communities you work in since you started implementing the DREAMS initiative? In what ways have they changed and why? What positive changes have there been? What negative changes have there been?
18. How do staff perceive and value the various DREAMS interventions? What do they think of the quality of the interventions delivered? How would you say DREAMS is affecting the workload of your staff?
19. What are the main opportunities that you/your organisation has experienced since the beginning of DREAMS?
20. What factors help/facilitate your DREAMS-related work?
21. What factors hinder/act as barriers to your DREAMS-related work
22. What recommendations do you have for improving the DREAMS intervention?
23. What do you think will happen to the work you currently do following the completion of the DREAMS intervention?

**Community and youth leaders**

1. Do you live in this community? How long have you lived in this community?
2. Can you tell me about your work here? [If applicable] How long have you been in this position?
3. Who are the most important HIV service providers here? What do they do and why are they important?
4. Have you heard about the DREAMS project? What do you know about the project/how would you describe it to someone who has never heard of it? What do you like about it? What don’t you like?
5. What were your expectations of DREAMS when you first started working on it?
6. Do you know of any DREAMS project implementing organisations in your community? Do you know of any activities they do?
7. Are there people in your village who have benefited from the DREAMS project? Please tell me about them.
8. Do you think the DREAMS project is having an impact in the community? If so, please tell me more about this impact?
9. Do you feel that the DREAMS intervention is affecting how people in this community think and behave (social norms, attitudes and behaviour)? e.g., in what ways is the DREAMS project influencing how families think and behave? Have you noticed any difference in how men think and behave? Have you noticed any changes in how AGYW think and behave? Please tell me more about these changes? *Ask what are the barriers/facilitators to this process*
10. Are there any aspects of DREAMS that you are uncomfortable with? Are there any aspects that you think don’t work well/are inappropriate for your community?
11. Have you made any adaptations or changes to the DREAMS interventions so that it better suits your organisation or beneficiaries? Please tell me about any innovations or changes that you have made to the intervention.
12. What suggestions do you have for improving the delivery and impact of the DREAMS project

**Local healthcare workers**

1. How long have you worked at this health centre? Please tell me a little about the work that you do on a day-to-day basis.
2. Who are the main beneficiaries that you serve?
3. Thinking about HIV prevention strategies, what types of services or interventions are you offering? Which of these strategies do you think are making an impact on HIV prevention in this community? Why? *Probe about which ones are popular amongst who and why? Probe about medical and traditional male circumcision (including preference, popularity), concept of ‘universal testing and treatment’, treatment of STIs and PMTCT*
4. Have you ever heard about DREAMS? Please describe DREAMS to someone who has never heard of it before?
5. What were your expectations of DREAMS when you first started working on it?
6. What DREAMS-related activities is your health facility involved in? When did the roll-out of the DREAMS activities start?
7. Do you think that DREAMS is reaching the right people in terms of those most at risk of HIV? Are there groups of people who are being left out? What kinds of people would benefit most from DREAMS and why etc.
8. In your view, how is the DREAMS-related service you are providing experienced by those it targets, and wider community members?
9. What do you think would help to improve your work and the services that you provide for AGYW?
10. What does success in implementing DREAMS look like? What kinds of things are helping you to move in that direction, and what kinds of things get in the way? What are the challenges you experience as you deliver DREAMS-related services?
11. Have you made any adaptations or changes to the DREAMS interventions so that it better suits your organisation or beneficiaries? Please tell me about any innovations or changes that you have made to the intervention.
12. In your view, in what ways have things changed in the communities you work in since you started implementing the DREAMS initiative? In what ways have they changed and why?
13. How do staff perceive and value the DREAMS-related services? What do they think of the quality of the interventions delivered?
14. Do you work with other DREAMS service providers? In what way do you work with them? Did you work with these organisations before the DREAMS intervention?
15. What suggestions do you have for improving the delivery and impact of the DREAMS project

**Closing**

We have come to the end of the interview. Before we finish, is there anything else that you would like to add about what we have discussed?

Do you have any other feedback about the program? What other information would you like to know about the program?

**Annex 1: Interview Cover Sheet**

Recorder Number: ___________________________

File Number:__________________________

**Participant details:**

Name of Interviewer(s): ____________________________

Date: ______________

Location [informal settlement]: ­­­­­­­­­­______________________

Interviewee’s Name: _____________________

Organization: ______________________________ (N/A for community and youth leaders)

Role: _____________

Sex [circle one]: FEMALE MALE

Age: ____________

**DREAMS IMPACT EVALUATION STUDY**

**Kenya**

# **Focus Group Discussion Guide for PARENT/GUARDIAN & OPINION LEADERS**

Good morning/afternoon. My name is_____________ and I will be leading this discussion. This is _____________, who will be helping me with the interview. We work for APHRC and are independent researchers. We are not responsible for the delivery or success of DREAMS, but wish to document what we can learn from DREAMS. The purpose of this group interview is to get your input on the experiences of girls in the society and explore your experiences and expectations of DREAMS interventions. This is not a test, but an activity to help us learn more on how best to secure the health of girls and young women who have a higher rate of HIV infection compared to boys and men, in order to improve or replicate programs like DREAMS. We will be taking notes, as well as recording this interview to make sure that we do not miss anything. We will use your feedback to better understand the role of girls in society and what might be done to reduce HIV infection in girls. A report will be prepared to share with other researchers and the Ministry of Health. Your name will not appear anywhere on this report. You can also refuse to answer any questions that make you feel uneasy.

Before we start, we would like to establish some ground rules:

- The questions we will ask you have no right or wrong answers. We are here to learn from you.
- It is alright to hold opinions that differ from others.
- We ask that only one person speak at a time and that no side conversations with your neighbours take place during the discussion.
- People tell things in a different way even though they may be talking about the same thing. So, even though you may think that someone else has already said something that is in-line with your own experiences or thoughts, we would still like to hear what you have to say.
- Please do not tell others who took part in the group discussion, especially if you know their names.
- Please do not share what was said in the group discussion with people outside of the group.

I would now like to turn on the digital recorder.

Icebreaker: Let us start by mentioning who our favourite musician or sports personality is?

Local Attitudes towards girls

1. How are adolescent girls of primary-school age perceived in your community? How are the girls treated as compared to boys? Why do you think this is? How about those who are out of school?
2. How are adolescent girls of secondary-school age perceived in your community? How are the girls treated compared to boys? Why do you think this is? How about those who are out of school?

Currently in your community, at what age do girls typically marry?

1. When do you think girls should marry? Whose decision is it for a girl to marry? Why is that the case? What are the alternatives to marriage for a girl?
2. Do you think it is more important to educate a boy or a girl? Is their education equally important? Why? Are you aware of any barriers to girls completing primary and Secondary School education? What expectation does your community have for the education of girls?
3. What are the opportunities in your community for girls to earn money? What are the barriers to girls earning money?

Experiences with the wider DREAMS and DREAMS type interventions

1. Please share with me what comes to your mind when you hear about HIV Prevention Programs? Have you heard of the HIV Prevention Program called ‘DREAMS? How did you first learn of the program called ‘DREAMS’? Have you participated in the program? What motivated you to become involved?
2. Apart from DREAMS, what services or programmes for HIV prevention are you aware of in your community?
3. How would you describe DREAMS to someone who has never heard about it before?
4. How are parents/guardians/caregivers and children involved in the DREAMS activities that you participate in?
5. How do you think HIV Prevention Programs are being received/accepted by the community? And by girls and young women in particular? What about their parents? What makes you say this?
6. Specifically about the DREAMS Program, how is it being received by the community? What about young women. What about parents? What makes you say this?
7. DREAMS is trying to offer a wide range of support to girls and young women (health, education, financial, counseling, etc). How well do you think DREAMS is linking/layering these different types of services?
8. Thinking about the girls and young women in your community what are your thoughts regarding the possible effects of DREAMS interventions? How do you think the DREAMS interventions will affect the lives of young women in your community? What specific DREAMS intervention (s) do you think is/are most beneficial?
9. What recommendations would you have for DREAMS to improve its impact/work? How could LVCT Health/ Hope Worldwide improve their DREAMS- related work?

Local understanding of HIV

1. How has HIV affected adolescents in your community?
2. What can be done to prevent young girls and women from contracting HIV?
3. What do you think about the communication between parents and their children regarding sexuality (HIV/STI’s, reproductive health, safe sex etc)? How can this communication be introduced/enhanced?
4. From what you have seen or heard, what can be done to help adolescents in your community to delay their sexual debut?
5. How do the community members react when they learn that someone is living with HIV? Is this reaction any different when the person is an adolescent girl or young woman? Is this reaction any different when the person is an adolescent boy?

Experience and expectation of reproductive health and sexual health care

1. Thinking about the young women in your community, what do they do to avoid or delay pregnancy? What influences what method a woman uses to avoid or delay pregnancy? Who usually makes the decision for a woman to begin to use a method that helps avoid or delay pregnancy?
2. Where do you think AGYW receive information around fertility and sex in your community?
3. From what you have seen or heard do you think AGYW and their sexual partners communicate about how to avoid or delay pregnancy? Are you aware of any services in your community that could help them to communicate about this?

Do you *have any questions? We now come to the end of the interview. Thank you so much for your time and thoughtful responses*

**Annex 1: Interview Cover Sheet**

Recorder Number: ___________________________

File Number:__________________________

**Participant details:**

Name of Interviewer(s): ____________________________

Date: ______________

Location [informal settlement]: ­­­­­­­­­­______________________

Interviewee’s Name: _____________________

Sex [circle one]: FEMALE MALE

Marital status [circle one]: NEVER MARRIED MARRIED WIDOWED SEPARATED/DIVORCED

Highest level of education: NO SCHOOLING PRIMARY SECONDARY HIGHER

Age: ____________

**DREAMS IMPACT EVALUATION STUDY**

**Kenya**

# **Focus Group Discussions with Social Asset Building Mentors/Facilitators**

The questions below are a guide. Use the interview as an opportunity to explore any particular issues or events that may affect the implementation of the DREAMS intervention, that may be barriers and facilitators to implementation, or that capture mentors’/facilitators’ and beneficiaries’ perceptions and value of the intervention.

Complete the cover sheet for each participant before beginning the interview (Annex 1)

**Questions/Themes**

1. I would like to begin by learning how you first heard about the DREAMS intervention?
2. How would you describe the DREAMS intervention to someone who has never heard about it?
3. Are you aware of any other organisations in your community that offer similar interventions as those offered under DREAMS?
4. What do you think is going well in the project? What is not going well? What suggestions do you have for improvement?
5. What effects do you think the DREAMS project has had on adolescent girls? How will it affect their lives in future? How will it affect the community in future?
6. What has your experience been as a mentor? What does your role involve? What do you like about being a mentor? What don’t you like about being a mentor? What challenges do you face as a mentor? How have you addressed the challenges so far?
7. Please tell me about any referrals you have made for AGYW in your group to other DREAMS interventions? How did you find the process of referring? Did you have sufficient tools etc to refer? Do you know if they attended? Do you know if they found it useful?
8. Do you ever interact with other mentors? Please tell me about this.
9. Do you ever interact with other community leaders or services providers in relation to DREAMS? Please tell me about this?
10. I would like to learn more about your safe spaces group. What does your safe spaces group involve? What is going well in your group? What is not going well? What has been the effect of safe spaces on the girls who participate? What challenges are you facing with the group? What can be done to address these challenges?
11. What do you think about the DREAMS curriculum (guide/activity book/manual)? What do you like about it? Why? What don’t you like about it? Why? Are there any specific sessions that are particularly challenging? Why? What changes would you recommend to the curriculum (guide/activity book/manual)? Why and why not?
12. Did you receive training to be a mentor? What did you like about the training? Do you think you received enough training to be able to do your role as a mentor well? What changes would you recommend to the training that you received to be a mentor?
13. Do you ever interact with girls in your group outside of the safe spaces meeting hours? Describe these meeting. What types of help/ problems do girls come to you with?
14. Overall, how is the attendance in your group? Why do girls miss meetings? Have you ever been concerned about attendance at the meetings? Have you ever taken any steps or sought support from other DREAMS implementers to try and improve attendance?
15. What do you expect the outcome will be for AGYW once they have completed the DREAMS programme?

**Closing**

We have come to the end of the interview. Before we finish, is there anything else that you would like to add about what we have discussed?

Do you have any other feedback about the program? What other information would you like to know about the program?

**Annex 1: Interview Cover Sheet**

Recorder Number: ___________________________

File Number:__________________________

**Participant details:**

Name of Interviewer(s): ____________________________

Date: ______________

Location [informal settlement]: ­­­­­­­­­­______________________

Interviewee’s Name: _____________________

Organization: ______________________________ (N/A for community and youth leaders)

Role: _____________

Sex [circle one]: FEMALE MALE

Age: ____________

**DREAMS IMPACT EVALUATION STUDY**

**Kenya**

# **Structured Observation Guide**

Observations will be made of 40 randomly selected DREAMS interventions in a range of settings, such as schools, safe spaces and health facilities. 20 will be done during early DREAMS implementation (late 2016/early 2017) and 20 will be done after DREAMS has been implemented for at least 18 months (late 2017/early 2018). We will focus on how participants interact with the service/material, and note what works well and what less well.

**Objectives**

- Observe a sample of the interventions delivered in context
- Observe the ways in which DREAMS is delivered and received, and with what quality and intensity, via structured observations (using observation check lists).

**A: Health facility/Organization/Safe spaces and schools observation form**

| Date |  |
| --- | --- |
| Day of the week |  |
| Time started |  |
| Time ended |  |
| Observer |  |
| Results of observation | 1 Completed  2 Partially completed  3 Refused |
| Name & address of health facility/Organization/School |  |
| Name of Venue |  |
| Services offered and activities conducted |  |
|  | |
| Targeted number of recipients /sessions for the intervention (to be obtained from implementing partner beforehand):  DREAMS Geographical coverage/ areas/ wards/locations covered:  Date intervention/activity was first implemented at this site: ___________________  **Resources available:**  Tools used:  Human resources:  Financial resources/budget:  Other resources:  Infrastructure for office and working space:  Computers and internet:  Telephones:  Administration support: | |

**B. Physical environment**

| **Where are the DREAMS activities delivered?**  At home  School  Community hall  Church  Clinic facility  Mobile unit  Other: Specify……………………………………………………………………. |  |
| --- | --- |
| **Which activities are being delivered? List them** |  |
| Is there a signboard that mentions the facilities operating hours? | Yes No N/A |
| Does it mention hours for DREAMS interventions/services? | Yes No N/A |
| **Does the waiting/receiving area:** Have adequate and comfortable seating? | Yes No |
| Describe the set-up of the room where the DREAMS activities are taking place?  Look out for:  Overall does it look friendly and welcoming? Clean?  Audio privacy?  What is the seating arrangement like?  How is the atmosphere– tense, exciting or boring?  How do people use the space? Do certain groups congregate in certain areas? |  |
| **Describe the interaction between the facilitators and the participants? What is the tone of verbal communication, what non-verbal communication/body language ques do you observe?** |  |
| Does the facilitator look confident and knowledgeable in his/her subject area? Do they focus their facilitation towards a certain group or do they involve all participants? |  |
| How is the intervention delivered?  Is it participatory?  One on one  Group sessions  Community  Road shows |  |
| How many participants are there? |  |
| What age groups are the participants? (tick all that apply) | 10-14 yr olds  15-19 yr olds  20-24 yr olds  25+ |
| What gender are the participants? (tick all that apply) | Females  Males |
| Please note any specific characteristic of the participants, if applicable (e.g., in / out of school youth; pregnant women; orphans; parents / caregivers; etc) |  |
| Additional notes on how the intervention is being delivered e.g. medium being used (radio, booklets, writing board?) |  |

**C. Privacy and confidentiality of venue/setting DREAMS interventions are delivered**

| **Check for visual and auditory privacy features** |  |
| --- | --- |
| Communication between reception staff and visitors is private and cannot be overheard, including from the waiting room. What is the reception staff’s manner? Friendly? Welcoming? Patient? Impatient? | Yes No NA |
| In the offices/examining rooms, there is a screen to separate the examination area from the consultation area. | Yes No NA |
| No one can see or hear the client from the outside during the consultation or counselling/mentorship – unless delivered in a group. | Yes No NA |
| If activity not clinical or in a clinic setting: Is the meeting place for activity suitable to facilitate smooth delivery of intervention and activities? | Yes No NA |
| Does the venue look conveniently situated for the recipients? | Yes No NA |
| **Check for confidentiality procedures and their application in practice.** |  |
| Information on the identity of the adolescent and young women and the presenting issue are gathered in confidence during registration. | Yes No NA |
| Adolescent and young women are offered anonymous registration if they wish. | Yes No NA |
| Records/information is kept in a secure place, accessible only to authorized personnel. |  |
| The registers are kept under lock and key outside operating hours. | Yes No NA |
| For electronically stored information, measures are applied to prevent unauthorized access. | Yes No NA |

**D. Registers, tools, records, guidelines, SOPs, training records etc.**

| **Check to see the following registers, tools and records.** |  |
| --- | --- |
|  |  |
| The register on the number of DREAMS beneficiaries by activities | Yes No NA |
| Register with M&E indicators | Yes No NA |
| The reporting forms have a format that allows the presentation of data disaggregated by age and sex. | Yes No NA |
| Referral register | Yes No NA |
| Check and record the data elements recorded in each register and list eg  Sex  Age  Date of Birth  ID |  |
| Check frequency with which the different activities/sessions/interventions are delivered |  |
| Check the materials that are being used for delivery of the DREAMS interventions and list the main topic areas/themes covered by each. Are the materials in good condition? Of sufficient quantity for the current activity/future activities |  |
| Check the Standard Operating Procedures available and list them here |  |
| Check the guidelines they are currently using and the dates and list here |  |
| Check for training register of facilitators and list the trainings offered and the ones planned for |  |

*Can you add question(s) to capture the linkages/layering of DREAMS, e.g., whether and how this activity is linked to other DREAMS interventions? Whether it receives clients from other activities, and refers participants onto other interventions?*

Observer notes (Please record any observations you have about the activity, which are not captured in the above checklist questions) ……………………………………………………………………………………………………………………………………………………………………………………………………………………………………………………………………………………………………………………………………………………………………………………………………………………………………………………………………………………………………………………………………………………………………………………………………………………………………………………………………………………………………………………………………………………………………………………………
