# Supplementary material for: Translating DREAMS into practice: Early lessons from implementation in six settings
Source: PLoS One. 2018 Dec 13;13(12):e0208243. doi: 10.1371/journal.pone.0208243 (PMC6292585; doi:10.1371/journal.pone.0208243)
Supplement: S2 File — (DOCX) [file pone.0208243.s002.docx]

**S2 File. DREAMS Impact Evaluation, Process Evaluation Tools, Kenya (Swahili)**

# **Qualitative longitudinal study with young people - in-depth interview guide for young men and women**

This is an in-depth interview and below is the topic guide with some open-ended questions that you can use. But the key is to probe and gain depth of information around the young persons experiences and expectations about DREAMS.

Complete the coversheet provided in Annex 1

**Questions/Themes**

**Personal**

1. I would like to begin by knowing more about you. What is important to you, how and where do you spend your time, what do you enjoy or don’t enjoy, who do you socialise with etc? [*As a way to establish rapport and get to know the participant Let the participant tell you anything about themselves, about their family and any other general information]*

***Ningependa kujua zaidi kukuhusu? Nini muhimu kwako? Huwa unatiumia muda wako kivipi na wapi? Huwa unafurahia nini na ni nini kisichokufurahisha? Kwa mara nyingi huwa unatangamana na kina nani?***

1. How long have you lived in this community?

***Umeishi katika hii jamii kwa muda gani****?*

1. What is your level of education? Probe - How far did you reach in completing your studies if you are no longer in school? What are the reasons for not being in school if you are supposed to be in school? If in school, ask what grade they are in.

***Kiwango chako cha elimu ni kipi? Ikiwa hauko shuleni kwa sasa, ulifika kiwango kipi? Ni sababu zipi zimekufanya usiwe shuleni? Ikiwa uko shuleni, uko kiwango kipi?***

1. If below 18 years, ask if their parents are alive? Probe who they live with and who is taking care of them. Also ask how many children are in their household and who is providing for the household financially?

***Je wazazi wako wako hai? (kama hawako hai) Hua unaishi na nani na mlezi wako ni nani? Kwenu mko watoto wangapi na ni nani mnayemtegemea kifedha?***

1. In the last week, have there been times when you were hungry but could not eat as there wasn’t any food? Was this the same for all people in your household or were some people more affected than others? Why? What do you usually do when this situation arises?

***Kwa wiki iliyopita ushawahi hisi njaa na ukakosa chakula? Hii ilikuwa sawa kwa watu wote ama wengine katika jamii yako waliadhirika kuliko wengine zaidi? Kwanini? Huwa unafanya nini wakati tukio kama hili linapotokea?***

**Experiences with the wider DREAMS and DREAMS type interventions**

1. Have you heard of a program called DREAMS? (probe using the names of the organisations that are working in the area)? How did you hear of the program called ‘DREAMS’?

***Umesikia kuhusu mradi unaitwa DREAMS? Ulisikia kuhusu mradi wa DREAMS kivipi?***

1. How would you describe DREAMS to someone who has never heard of it before?

***Unaweza elezea kivipi huu mradi wa DREAMS kwa mtu ambaye hajawahi sikia lolote kuuhusu?***

1. How did you come to be involved with DREAMS? Please tell me about the first time you met anyone from DREAMS or participated in a DREAMS activity.

***Uliweza kujihusisha na mradi wa DREAMS kivipi? Tafadhali nielezee kuhusu mara ya kwanza ulipopatana na mtu kutoka mradi wa DREAMS ama mara ya kwanza uliposhiriki katika mkakati wa mradi wa DREAMS?***

1. What were your expectations when you first heard about DREAMS?

***Matarajio yako yalikuwa yapi uliposikia kuhusu huu mradi wa DREAMS kwa mara ya kwanza?***

***Ask question 5, 6 and 8 only if involved in DREAMS intervention/s***

1. I would like to know more about your experience of DREAMS from the beginning of your involvement until now. As I shared earlier, this interview is totally confidential, and will not be shared with the people you know at DREAMS. So please feel free in telling me about how you feel about DREAMS activities. Most people like some things more than others – Which DREAMS activities have you been involved with? What have you enjoyed most and why? Which parts do you like less and why? What would you change about DREAMS if you could? Is DREAMS better/worse or as you expected? What makes you say this?

***Ningependa kujua kuhusu yale umepatana nayo katika mradi wa DREAMS tangu uanze kuhusika nao hadi sasa? Vile nilivyoeleza hapo awali haya mahojiano ni ya kisiri na hayatasambazwa kwa watu unaojua katika mradi wa DREAMS kwa hivyo jihisi huru kuniambia kuhusu hisia zako juu ya mradi wa DREAMS. Kwa kawaida watu hupenda vitu tofauti, kwako wewe umejihusisha na huduma ipi ya DREAMS? Ni gani ulipendelea Zaidi na kwanini? Na ni sehemu gani haukupendelea sana na kwanini? Ni nini ambacho ungebadilisha katika DREAMS ungelikuwa na uwezo? Mradi huu wa DREAMS uko sawa zaidi ama mbaya zaidi au uko kama ulivyotarajia? Nini chakufanya useme hivi?***

1. **(If AGYW)** Please tell me about your DREAMS mentor. What was your first meeting like? How does she help you? What do you like about your relationship with her? What do you not like about your relationship with her? Is there something that you wish your mentor could do to help and support you that she is currently not doing?

***Nieleze Zaidi kuhusu mshauri wako wakuiga katika mradi wa DREAMS? Mkutano wako wa kwanza ulikuaje? Anakusaidiaje? Ni nini linakupendeza kwa uhusiano wako nayeye? Ni nini kisichokupendeza kwa uhusiano wako nayeye? Kuna jambo lolote ungependa mshauri wako akusaidie nalo lakini halifanyi kwa sasa?***

*Ask all: probe through list of interventions for those that did not mention being involved in DREAMS interventions, if not participating ask question 9.*

1. Are you participating in [name of DREAMS type intervention]? **(If not participating in any, skip to question 9)**

***Je, unashiriki na [ Jina la huduma ya mradi wa DREAMS]***

- 1. Which organization is providing the intervention?

***Huduma hiyo ilipeanwa kutoka wapi/kupitia shirika gani?***

- 1. When did you start participating in the activity?

***Ulianza kushiriki huduma hii lini?***

- 1. How often did you participate?

***Huwa unashiriki katika hii huduma kwa mara ngapi?***

- 1. Are you still participating in the activity? *If they are no longer participating in the activity ask why they stopped*.

***Ungali unashiriki katika huduma hii? [Kama hashiriki, muulize mbona aliacha]***

- 1. What do you like about the activity?

***Ni nini kinachokupendeza katika huduma hii?***

- 1. What things could be done better?

***Ni mambo yapi yanayoweza boreshwa katika huduma hii?***

- 1. What things would you like but are not available?

***Ni mambo yapi ambayo ungependa lakini hayapatikani kwa sasa katika huu mradi?***

- 1. Have you told anyone that you are involved with DREAMS? Who did you tell? What did they think of your participation?

***Je, umeelezea yeyote kwamba unahusika katika huu mradi wa DREAMS? Ulimwelezea nani? Walifikiria nini kuhusu kujihusisha kwako katika huu mradi wa DREAMS?***

- 1. Have you ever hidden your participation in DREAMS from anyone? Who? Why? What do you think would happen if they found out?

***Ushawahi kana kuhusika kwako katika huu mradi wa DREAMS kwa yeyote? Nani? Kwanini? Unafikiria ni nini kinaweza fanyika wangalijua kuwa unajihusisha na huu mradi?***

- 1. Are your parents/guardians/caregiver involved in these activities? *Probe for reasons why/why are they not involved– is it because of the sensitivity eg condom use or contraception? Explain if this is affecting/will affect continued access/utilisation?*

***Wazazi/walezi wako huwa wanajihusisha na huduma hizi? Mbona hawajihusishi? Je, nikwasababu hili ni swala nyeti? Kwa mfano utumiaji wa kondomu au upangaji wa uzazi?***

| ***DREAMS project interventions:***   1. Social Assets Building: Structured small groups led by mentors to build the knowledge, skills and networks. 2. HIV Testing Service: Combination of services that include counseling, HIV testing, condom distribution, risk assessment and risk reduction counselling 3. Evidence based behavioral interventions: Healthy Choices for a Better Future – targeting 10-14 years old, My Health My Choice targeting 13-17 years old and Families Matter! Program targeting caregivers of 9-12 years old 4. Education support: Targeting mainly transitional AGYW and those who dropped out of school and which to continue. Transition from primary to secondary 5. Long term contraceptive mixed methods provide mainly through partnerships and referral. This also includes family planning education. 6. STI screening and treatment. Mainly integrated with other interventions like breast cancer screening, HTS, TB screening and family planning 7. Condom distribution and education 8. Male Sexual Partner Characterization: Profiling the men who are likely sexual partners to the AGYW so as to target them with appropriate intervention. It’s done by the mentors in the safe spaces 9. Vocational training and apprenticeship skills: AGYW are supported in building skill that will help them earn money 10. Entrepreneurship training to equip the girls with business skills and encourage savings through table banking 11. Post violence care. Services provided on site include psychosocial support and services through referral include but not limited to legal support, medical attention 12. Community mobilization and norm change interventions that aim to change the societal norms around women. Key area of focus is on gender based violence |
| --- |

1. Were you ever referred from one place where you received a service that is part of DREAMS to another one e.g. from HIV testing centre to a centre that offers services for preventing mother to child transmission of HIV? How did it feel to be referred? Were you pleased by this? Did you go to the place that they referred you? When? Did you go by yourself or did you go with someone?

***Ushawahi tumwa kutoka huduma moja ya mradi wa DREAMS hadi huduma nyingine? Kwa mfano eneo la huduma ya kupimwa virusi vya ukimwi hadi eneo lingine la kuzuuia uambukizaji wa virusi kutoka kwa mama hadi kwa mtoto? Ulihisi vipi ulipoelekezwa huko? Ulipendekezwa na kuelekezwa huku? Ulienda mahali uliokoelekezwa? Lini? Ulienda mwenyewe ama uliandamana na mtu?***

1. Since DREAMS started, have you noticed any changes in your local community, among your friends and peers, or for yourself? ? What kinds of changes have you seen, if any what positive changes have you seen? What negative changes have you seen?

***Tangu mradi wa DREAMS uanze, ushawahi ona mabadiliko yoyote katika jamii yako miongoni mwa marafiki wako ama watu wa rika lako, au kwako mwenyewe? Umeshuhudia mabadiliko yapi? kama kunayo nielezee ni mabadiliko yapi mema na yasiyo mema ambayo umeshuhudia?***

**Experience and expectation of reproductive and sexual health information and services**

1. Have you used any healthcare facility over the past year? Which one did you use? What has been your experience? What were the good things? What were the bad things? What could be better?

***Kwa mwaka mmoja uliopita, ushawahi tumia kituo cha afya chochote? Ulihudumiwa katika kituo gani? Ulikumbana na mambo yapi katika kituo hiki? Nielezee kilichokufurahisha na ambacho hakikukufurahisha? Ni nini kingekua bora zaidi?***

1. Where/from whom do young people your age in this community receive information about sexual relationships and reproductive health?

***Vijana wa rika lako katika jamii hii hupata ujumbe kuhusu uhusiano wa kimapenzi na afya ya uzazi wapi?***

1. Have you ever received information about sexual relationships and reproductive health? What do you feel about the way in which you were taught about these topics? What did you like? What did you not like? [probe about the adequacy of the information received]. Do you wish you had more access to information about sexual relationships and reproductive health or is the information you have access to enough?

***Ushawahi kupata ujumbe kuhusu uhusiano wa kimapenzi na afya ya uzazi? Unahisi vipi kuhusu jinsi ulivyofunzwa kuhusu mambo haya? Ni nini kilichokupendeza? Ni nini ambacho hakikukupendeza? Unahisi ingekuwa bora zaidi kama ungepata ujumbe zaidi kuhusu uhusiano wa kimapenzi na afya ya uzazi au ujumbe ambao unao unatosha?***

1. **[for females aged 15 and older]** Where do women in this community go for contraceptive or family planning services? Have you ever used these services?
   1. [IF YES]What was your experience using the services?
   2. [IF NO] Do you know where to go for these services? Would you feel comfortable asking for contraception? Why? Why not?

***Wanawake katika hii jamii huendea wapi huduma za kuzuia uja uzito au kupanga uzazi? Ushawahi tumia huduma hizi?***

1. ***Ulikumbana na mambo gani wakati ulipopata hizi huduma***
2. ***Wajua ni wapi unaweza enda kupokea hizi huduma? Unaweza kuwa na shida yeyote kuulizia huduma za kuzuia uja uzito? Kwanini?***
3. [for males aged 15 and older] Have you ever heard of voluntary male medical circumcision (VMMC)? Where did you hear about it? Do you personally know of anyone who has had VMMC? Would you consider it? Why/ why not? Have you undergone VMMC? What was your experience?

***Ushawahi kupata ujumbe kuhusu VMMC ? Ulipata ujumbe huu kutoka wapi? Wewe unajua mtu yeyote ambaye amepata hii huduma ya VMMC? Wewe binafsi unaweza zingatia huduma hii? Kwanini? Wewe mwenyewe umepata huduma hii ya VMMC? Ulikabiliana na mambo gani wakati ulipata hii huduma?***

**Experience and expectation of HIV testing**

1. Have you ever tested for HIV?

***Umewahi pimwa virusi vya ukimwi?***

- 1. [IF YES] Where did you get tested? How long ago did you get tested? Did you get your results? What was your experience? Were you referred by someone or did you just go? Did you go by yourself or did someone else go with you? Did they also test? What could be done better? Would you recommend that setting? Why/Why not?

**Do not ask for the participant’s HIV test results,** but if they volunteer that they are positive, probe their experience of HIV care

***Ulipimwa wapi? Ulipimwa lini? Ulipata matokeo? Ulikabiliana na mambo gani ulipoenda kupimwa? Ulielekezwa na mtu kuenda kupimwa ama ulijiamulia wewe mwenyewe? Je, ulienda peke yako ama uliandamana na mtu mwingine? Kama uliandamana na mtu, yeye alipimwa? Ni nini ambacho kingefanywa bora zaidi? Unaweza elekeza mtu mwingine kwenda kupata huduma hii huko ulikopata? Kwanini?***

- 1. [IF NO] Is there anything that has prevented you from being tested? What would make it easier for you to test? Do you know where to get a test? Would you feel comfortable going there for the test? Why/ why not?

***Kuna kitu kinachokuzuia kupimwa? Ni nini kinachoweza kurahisisha wewe kupimwa? Wajua mahali ambapo unaweza enda kupimwa? Unaweza kuwa na shida yoyote kwenda huko kupimwa? Kwanini?***

**General observations on DREAMS related activities/issues**

1. **What does the word ‘empowered’ mean to you?**

**a.** Do you feel empowered? Why or why not? (If yes, in what way /how do you feel empowered?) What do would be necessary for you to feel empowered in this community?

b. Do you feel hopeful about your future? If yes, what makes you hopeful about your future? If not, why not? What are your main concerns about your future/what factors do you think might prevent you from meeting your hopes for your future? What do you think would be necessary for you to feel more hopefully about your future?

***Je, neno kuwezeshwa au kupewa uwezo lamaanisha nini kwako?***

- 1. ***Je, wewe mwenyewe wajihisi kama umewezeshwa au umepewa uwezo? Mbona wahisi umewezeshwa au mbona wahisi hujawezeshwa? Kama umewezeshwa au umepewa uwezo, wahisi umewezeshwa au umepewa uwezo kivipi? Wahisi ni nini kingelikuwa muhimu kwako ili uhisi ya kwamba umewezeshwa au umepewa uwezo katika jamii hii?***
  2. ***Je, uko na matumaini kuhusu maisha yako ya usoni au ya baadaye? Kama uko na matumaini, ni nini ambacho chakupa matumaini kuhusu maisha yako ya baadaye? Kama hauna matumaini kuhusu maisha yako ya baadaye, mbona huna haya matumaini? Je, ni mambo yapi ambayo ni muhimu sana kwako kuhusu maisha yako ya baadaye? Ni mambo gani ambayo wewe wafikiria kuwa yanaweza kukuzuia kuafikia malengo na matumaini ya maisha yako ya baadaye? Je, wafikiria ni nini kingelikuwa muhimu kwako ili kukuwezesha kuwa na matumaini kuhusu maisha yako yako ya baadaye?***

1. **(If DREAM girl )** What kind of knowledge have you acquired since becoming a DREAMS girl? Can you describe your experience before and after being on DREAMS? How/Do you feel you are now more capable/or not of making decisions about your health (do not probe unless it’s not clear) eg engaging in safer sexual practises, condom use, getting an HIV test, delaying getting pregnant; education eg finishing school, doing well in school, proceeding to a higher level grade etc and socio-economic planning eg saving money or just knowing how to save money, etc (whether or not because of DREAMS)? Have you put what you have learnt from DREAMS in action – can you tell me about it? If not – what has stopped you?

***(Wasichana wa DREAMS) Je, ni ujuzi gani / elimu gani ama ni ufahamu upi ambao umepata tangu uanze kuhusishwa katika mradi huu wa DREAMS? Tafadhali nieleze kuhusu hali ya maisha yako / jinsi maisha yako ilivyokuwa kabla na baada ya kuhusishwa katika mradi wa DREAMS. Je, wahisi kwa sasa kwamba uko na uwezo kufanya maamuzi kuhusu afya yako? Kwa mfano, kujihusisha au kuhusika katika ngono salama isiyo na hatari, kutumia mipira ya kondomu wakati wa ngono, kupimwa virusi vya ukimwi, kuzuia au kuchelewesha ujauzito; katika masomo, kumalizia masomo yako, kufanya vyema au kufaulu katika shule, kuendelea na masomo had kiwango cha juu; na katika maswala ya kijamii na kifedha, kujiwekea akiba au kujua jinsi ya kuweka akiba; Je, umeweza kupata huu uwezo kutokana na mradi wa DREAMS au ni kutokana na nini hasa? Je, umeweza kuyatekeleza yale uliyojifunza katika mradi wa DREAMS? Tafadhali nieleze kuhusu hayo. Kama hujaweza kuyatekeleza yale uliyojifunza, ni nini amabcho chakuzuia kuyatekeleza?***

1. **(If ‘DREAM boy’/ABYM involved with DREAMS/heard about DREAMS)** Is DREAMS engaging ABYM? **[If yes]** How is DREAMS engaging ABYM? How has DREAMS impacted on ABYM? Do you feel it is a success? If yes, how and why?

**[If no]** Is it important for DREAMS to engage ABYM? Why? How should they be engaged?

***(Wavulana wa DREAMS) Je, mradi wa DREAMS unahusisha au unalenga wavulana waliobaleghe na vijana wanaume? Je, mradi wa DREAMS unawahusisha au unawashirikisha kivipi wavulana waliobaleghe na vijana wanaume? Je, mradi wa DREAMS umewabadilisha au umewaathiri kivipi wavulana waliobaleghe na vijana wanaume? Kunayo matokeo yoyote ambayo umeyashuhudia? Je, wahisi au wafikiri ya kwamba huu mradi wa DREAMS umefaulu? Kama umefaulu, umefaulu kivipi na kwa nini?***

***(Kama Wavulana waliobaleghe na vijana wanaume hawajahusishwa) Je, kuna umuhimu wowote kwa mradi wa DREAMS kuhusisha na kushirikisha wavulana waliobaleghe na vijana wanaume? Mbona wahusishwe? Wafikiri ni kwa njia gani mradi wa DREAMS waweza kuhusisha au kushirikisha wavulana waliobaleghe na vijana wanaume?***

1. What challenges do AGYWs face in this community in their daily lives? How do they overcome these challenges?

***Wasichana waliobaleghe and na vijana wanawake wanakubana na changamoto zipi katika jamii katika maisha yao ya kila siku? Je, wanakabilianaje na changmoto hizi?***

1. When going about your normal activities, do you feel safe in your community? Why/why not? What risks do you face in your community – please think about things like social risks, physical risks, health risks and risks related to your community.
   1. What factors lead to this risk (probe eg gender, age, poverty, class, ethnicity?)
   2. What factors help you to reduce these risks (probe eg family, friends, formal community structures eg police, chief etc, various organisations, DREAMS, faith/religion?

***Je, huwa unahisi ukiwa salama unapoendelea na hali ya maisha yako ya kila siku katika jamii? Mbona wahisi kuwa uko salama / Mbona wahisi kuwa huko salama? Je, ni hatari gani ambazo unakubana au unakabiliana nazo katika jamii – zingatia mambo kama hatari za kijamii, hatari za kimwili, hatari za kiafya na hatari zinazohusiana na jamii yako.***

***a. Je, ni sababu gani au ni mambo gani ambayo yanachangia kuleta hizi hatari (Zingatia au ulizia mambo au sababu kama jinsia, umri, umaskini, matabaka ya kijamii, kabila)***

***b. Je, ni sababu gani au ni mambo gani huwa yanasaidia kupunguza hizi hatari (Zingatia au ulizia mambo au sababu kama familia, marafiki, mifumo rasmi ya jamii kama polisi, chifu, mashirika katika jamii, mradi wa DREAMS, Imani na Dini)***

1. Has DREAMS’ changed the social behaviour (e.g attitudes towards/interaction with friends, peers, relationships, other community members e.t.c) of AGYW? If yes, what are they doing differently?

***Je, mradi wa DREAMS umebadilisha mienendo ya kijamii/uma [kwa mfano fikra kusiana/ mwingiliano na marafiki, riika, uhusiano na wana kijiji wengine nakadhalika] wa wasichana waliobalehe na wanawake wachanga[AGYW] Kama ndio, nini wanafnya kitofauti?***

1. Is it okay for some programs to focus on AGYW to better their lives? Why? Why not?

***Ni vyema kwa miradi mengine kuangazia wasichana waliobalehe na wanawake wachanga [AGYW] ili kuboresha hali yao ya maisha? Kwa nini? Kwa nini sivyo?***

**[For males and females 15 years and above]**

***[Kwa wanaume na wanawake wa miaka 15 na Zaidi]***

1. Thinking about ways to prevent HIV infection, what types of strategies/interventions/services are mostly used by AGYW in this community? Why? What about ABYM? Why? Where do they access/ who provides these services? How effective do you think the strategies have been in prevention of HIV among AGYW/ABYM? Why?

***Hebu kwa kufikiria njia za kuzuia maambukizi ya virusi vya ukimwi (HIV), ni aina gani za mbinu/kuingilia kati/huduma zinazotumika sana sana kwa wasichana waliobalehe na wanawake wachanga[AGYW] katika jamii hii? Kwa nini? Na niaje kuhusu wanaume waliobalehe na wanaume wachanga[ABYM]? kwa nini? Ni wapi wanazipata huduma/nani hutoa hizi? Unafikiria mbinu hizi zimefaulu kwa kiasi gani kwa kuzuia maabukizo za virusi vya ukimwi [HIV] kwa wasichana waliobalehe na wanawake wachanga[AGYW]/ wanaume waliobalehe na wanaume wachanga[ABYM]. Kwa nini?***

1. Which are the preferred health related service providers/facilities among AGYW? Why? What about ABYM? Why? Do the preferences affect their health seeking behaviour for HIV related services? Does it affect their health seeking behaviour for any other health related issues?

***Ni vituo vipi vya kutoa huduma zinazohusika na afya ambavyo vinapendwa na wasichana waliobalehe na wanawake wachanga[AGYW]? Na je wanaume waliobalehe na wanaume wachanga[ABYM]? Kwa nini? Je mapendekezo hayo huathiri mienendo yao ya kutafuta huduma za afya zinazohusiana na virusi vya ukimwi (HIV? Je huwa inathiri mienedndo yao ya kutafuta huduma zingine ambozo zinahusiana na afya?***

**Closing**

***Kufunga***

We have come to the end of the interview. Before we finish, is there anything else that you would like to add about what we have discussed?

***Tumefika tamati ya mahojiano yetu. Kabla tutamatishe, kunalo jambo lolote ambalo ungependa kuongezea kwa hayo tumeshaa jadili?***

Do you have any other feedback about the program? What other information would you like to know about the program?

***Kuna jambo lolote lingine unaweza taka kutuambia kuhusu huu mradi wa DREAMS?***

***Mambo mengine gani ungependa kujua kuhusu mradi wa DREAMS?***

**Annex 1: Interview Cover Sheet**

Recorder Number: ___________________________

File Number:__________________________

**Participant details:**

Name of Interviewer(s): ____________________________

Date: ______________

Location [informal settlement]: ­­­­­­­­­­______________________

Interviewee’s Name: _____________________

Sex [circle one]: FEMALE MALE

Marital status [circle one]: NEVER MARRIED MARRIED WIDOWED SEPARATED/DIVORCED

Schooling status: IN SCHOOL OUT-OF-SCHOOL

Highest level of education: NO SCHOOLING PRIMARY SECONDARY HIGHER

Age: ____________

**DREAMS IMPACT EVALUATION STUDY**

**Kenya**

# **Focus Group Discussion Guide for PARENT/GUARDIAN & OPINION LEADERS**

Good morning/afternoon. My name is_____________ and I will be leading this discussion. This is _____________, who will be helping me with the interview. We work for APHRC and are independent researchers. We are not responsible for the delivery or success of DREAMS, but wish to document what we can learn from DREAMS. The purpose of this group interview is to get your input on the experiences of girls in the society and explore your experiences and expectations of DREAMS interventions. This is not a test, but an activity to help us learn more on how best to secure the health of girls and young women who have a higher rate of HIV infection compared to boys and men, in order to improve or replicate programs like DREAMS. We will be taking notes, as well as recording this interview to make sure that we do not miss anything. We will use your feedback to better understand the role of girls in society and what might be done to reduce HIV infection in girls. A report will be prepared to share with other researchers and the Ministry of Health. Your name will not appear anywhere on this report. You can also refuse to answer any questions that make you feel uneasy.

Before we start, we would like to establish some ground rules:

- The questions we will ask you have no right or wrong answers. We are here to learn from you.
- It is alright to hold opinions that differ from others.
- We ask that only one person speak at a time and that no side conversations with your neighbours take place during the discussion.
- People tell things in a different way even though they may be talking about the same thing. So, even though you may think that someone else has already said something that is in-line with your own experiences or thoughts, we would still like to hear what you have to say.
- Please do not tell others who took part in the group discussion, especially if you know their names.
- Please do not share what was said in the group discussion with people outside of the group.

I would now like to turn on the digital recorder.

Icebreaker: Let us start by mentioning who our favourite musician or sports personality is?

Local Attitudes towards girls

1. How are adolescent girls of primary-school age perceived in your community? How are the girls treated as compared to boys? Why do you think this is? How about those who are out of school?

***Je, wasichana ambao wako shule ya msingi na walio balehe wanaonekana kivipi katika jamii yenu? Ukilinganisha na wavulana, wasichana wanachukuliwa kivipi? Je, unafikiria ni kwanini wanachukuliwa hivi? Na wale ambao hawako shuleni je?***

1. How are adolescent girls of secondary-school age perceived in your community? How the girls are treated compared to boys? Why do you think this is? How about those who are out of school?

***Je, wasichana ambao wako katika shule ya upili na walio balehe wanaonekana kivipi katika jamii yenu? Ukilinganisha na wavulana, wasichana wanachukuliwa kivipi? Je, unafikiria ni kwanini wanachukuliwa hivi? Na wale ambao hawako shuleni?***

Currently in your community, at what age do girls typically marry?

***Kwa sasa katika jamii yenu, wasichana huolewa wakiwa umri gani kwa kawaida?***

1. When do you think girls should marry? Whose decision is it for a girl to marry? Why is that the case? What are the alternatives to marriage for a girl?

***Je unafikiria wasichana wanafaa kuolewa wakati gani? Nani huamua msichana aolewe? Kwa nini?***

***Bali na kuolewa msichana anaweza jihusisha na yapi mengine?***

1. Do you think it is more important to educate a boy or a girl? Is their education equally important? Why? Are you aware of any barriers to girls completing primary and Secondary School education? What expectation does your community have for the education of girls?

***Je, wafikiri ni muhimu sana kuelimisha mvulana ama musicha? Je, masomo yao ni ya muhimu kwa usawa au la? Kwa nini? Kuna vizuizi gani zinazowazuia wasichana kukamilisha masomo yao ya msingi na ya upili? Watu katika jamii hii wana matarajio yapi kutokana na masomo ya wasichana?***

1. What are the opportunities in your community for girls to earn money? What are the barriers to girls earning money?

***Wasichana katika jamii yenu wana nafasi zipi za kupata fedha? Kuna vizuizi gani kwa wasichana kupata fedha?***

Experiences with the wider DREAMS and DREAMS type interventions

1. Please share with me what comes to your mind when you hear about HIV Prevention Programs? Have you heard of the HIV Prevention Program called ‘DREAMS? How did you first learn of the program called ‘DREAMS’? Have you participated in the program? What motivated you to become involved?

***Tafadhali nieleze nini kinacho kuja kwa akili unaposikia kuhusu miradi ya kuzuia virusi vya ukimwi? Je, ushawahi kusikia kuhusu mradi wa kuzuia virusi vya ukimwi ambao unaitwa DREAMS? Je, ulikuja kujua kuhusu mradi wa DREAMS vipi? Ushawahi kujihusisha na huu mradi? Nini kilicho kupatia motisha wa kujihusisha katika mradi huu?***

1. Apart from DREAMS, what services or programmes for HIV prevention are you aware of in your community?

***Kando na DREAMS, ni huduma gani ama miradi ya kuzuia virusi vya HIV ambazo unazifahamu katika jamii yenu?***

1. How would you describe DREAMS to someone who has never heard about it before?

***Unaeza kueleza mradi wa DREAMS vipi kwa mtu ambaye hajawahi kuusikia?***

1. How are parents/guardians/caregivers and children involved in the DREAMS activities that you participate in?

***Wazazi/walezi na wasichana wanajihusisha kivipi katika huduma za DREAMS ambazo unajihusisha nazo?***

1. How do you think HIV Prevention Programs are being received/accepted by the community? And by girls and young women in particular? What about their parents? What makes you say this?

***Je, miradi ya kuzuia virusi vya HIV vinapokewa kivipi katika jamii? Na haswa wanawake wadogo? Je, na wazazi wao? Nini chakufanya useme hivi?***

1. Specifically about the DREAMS Program, how is it being received by the community? What about young women? What about parents? What makes you say this?

***Mradi wa DREAMS unapokelewa kivipi katika jamii? Na kwa wanawake wadogo? Na wazazi? Nini inakufanya useme hivi?***

1. DREAMS is trying to offer a wide range of support to girls and young women (health, education, financial, counseling, etc). How well do you think DREAMS is linking/layering these different types of services?

***Mradi wa DREAMS unajaribu kupeana usaidizi kwa wasichana na wanawake wadogo (afya, masomo, fedha, ushauri). Je, unafikiria hizi huduma tofauti zina husishwa/kuchanganywa vizuri kivipi na huu mradi wa DREAMS?***

1. Thinking about the girls and young women in your community what are your thoughts regarding the possible effects of DREAMS interventions? How do you think the DREAMS interventions will affect the lives of young women in your community? What specific DREAMS intervention (s) do you think is/are most beneficial?

***Ukifikiria kuhusu wasichana na wanawake wadogo katika jamii yenu, ni athari gani zinaweza kutokea kwasababu ya mradi wa DREAMS? Je mradi huu wa DREAMS utaathiri maisha ya wanawake wadogo katika jamii yenu kivipi? Na unafikiri ni huduma ipi/zipi haswa zina faida sana?***

1. What recommendations would you have for DREAMS to improve its impact/work? How could LVCT Health/ Hope Worldwide improve their DREAMS- related work?

***Ni mapendekzo yapi unaeza kuwa nayo ili kuboresha athari/kazi ya mradi huu wa DREAMS? LVCT health/ Hopeworldwide wanaweza kuboresha vipi kazi yao ambayo inajihusisha na DREAMS?***

Local understanding of HIV

1. How has HIV affected adolescents in your community?

***Virusi vya ukimwi vimeathiri vijana waliobalehe kivipi?***

1. What can be done to prevent young girls and women from contracting HIV?

***Nini kinaweza kufanywa ili kuzuia wasichana wadogo kupata HIV?***

1. What do you think about the communication between parents and their children regarding sexuality (HIV/STI’s, reproductive health, safe sex etc)? How can this communication be introduced/enhanced?

***Una fikra zipi kuhusu mazungumzo kati ya wazazi na watoto wao kuhusu mambo ya ngono (Virusi vya ukimwi/magonjwa ya zinaa, afya ya uzazi, ngono salama na kadhalika)? Je mazungumzo haya yanaweza kuanzishwa ama kuendelezwa kivipi?***

1. From what you have seen or heard, what can be done to help adolescents in your community to delay their sexual debut?

***Kulingana na chenye umeona ama kuskia, nini ambacho kinaweza kufanywa ili kusaidia vijana waliobalehe katika jamii yenu kuepuka kuhusika na ngono mapema?***

1. How do the community members react when they learn that someone is living with HIV? Is this reaction any different when the person is an adolescent girl or young woman? Is this reaction any different when the person is an adolescent boy?

***Watu wa jamii hufanyaje wanapogundua ya kwamba mtu ana virusi vya ukimwi? Je, tendo hili ni tofauti ikiwa mtu huyu ni msichana aliye balehe ama mwanamke mdogo? Je, tendo hili ni tofauti ikiwa huyu mtu ni kijana aliyebalehe?***

Experience and expectation of reproductive health and sexual health care

1. Thinking about the young women in your community, what do they do to avoid or delay pregnancy? What influences what method a woman uses to avoid or delay pregnancy? Who usually makes the decision for a woman to begin to use a method that helps avoid or delay pregnancy?

***Ukifikiria kuhusu wanawake wadogo katika jamii yenu, ni mbinu gani wanatumia ili kuzuia ama kuchelewesha mimba? Nini hushawishi njia ambayo mwanake hutumia ili kuzuia ama kuchelewesh mimba? Nani huamulia mwanamke njia ambayo ataanza kutumia ili kuzuiya ama kuchelewesha mimba?***

1. Where do you think AGYW receive information around fertility and sex in your community?

***Wasichana waliobalehe na wanawake wadogo katika jamii yenu hupata wapi ujumbe kuhusu uzazi na ngono?***

1. From what you have seen or heard do you think AGYW and their sexual partners communicate about how to avoid or delay pregnancy? Are you aware of any services in your community that could help them to communicate about this?

***Kulingana na chenye umeona ama kusikia, je unafikiria ya kwamba wasichana waliobalehe na wanawake wadogo huzungumza na wapenzi wao kuhusu njia za kuzuia ama kuchelewesha mimba? Je, una ujuzi kuhusu huduma zozote katika jamii yenu ambazo zinaweza kuwasaidia kuzungumza kuhusu haya?***

Do you *have any questions? We now come to the end of the interview. Thank you so much for your time and thoughtful responses*

***Je, una maswali yeyote? Tumefika mwisho wa mahojiano. Asante sana kwa muda wako na majibu yako.***

**Annex 1: Interview Cover Sheet**

Recorder Number: ___________________________

File Number:__________________________

**Participant details:**

Name of Interviewer(s): ____________________________

Date: ______________

Location [informal settlement]: ­­­­­­­­­­______________________

Interviewee’s Name: _____________________

Sex [circle one]: FEMALE MALE

Marital status [circle one]: NEVER MARRIED MARRIED WIDOWED SEPARATED/DIVORCED

Highest level of education: NO SCHOOLING PRIMARY SECONDARY HIGHER

Age: ____________

**DREAMS IMPACT EVALUATION STUDY**

**Kenya**

# **Focus Group Discussions with Social Asset Building Mentors/Facilitators**

The questions below are a guide. Use the interview as an opportunity to explore any particular issues or events that may affect the implementation of the DREAMS intervention, that may be barriers and facilitators to implementation, or that capture mentors’/facilitators’ and beneficiaries’ perceptions and value of the intervention.

Complete the cover sheet for each participant before beginning the interview (Annex 1)

**Questions/Themes**

1. I would like to begin by learning how you first heard about the DREAMS intervention?

***Ningependa kwanza kujua vile ulipata kuskia kuhusu mradi wa DREAMS kwa mara ya kwanza?***

1. How would you describe the DREAMS intervention to someone who has never heard about it?

***Unaeza kueleza vipi kuhusu mradi wa DREAMS kwa mtu ambaye hajawahi kusikia kuihusu?***

1. Are you aware of any other organisations in your community that offer similar interventions as those offered under DREAMS?

***Je, unafahamu kuhusu shirika zingine katika jamii yenu ambazo zinapeana huduma sawa na zinazopeanwa kwa mradi wa DREAMS?***

1. What do you think is going well in the project? What is not going well? What suggestions do you have for improvement?

***Nini ambacho unafikiria kinaenda vizuri katika mradi? Nini ambacho hakiendi vizuri? Una mapendekezo yapi ili kuboresha mradi huu?***

1. What effects do you think the DREAMS project has had on adolescent girls? How will it affect their lives in future? How will it affect the community in future?

***Unafikiria ni athari gani ambazo zimetokana na DREAMS kwa wasichana waliobalehe? DREAMS itakua na athari gani katika maisha yao ya usoni? DREAMS itakuwa na athari gani kwa jamii siku za usoni?***

1. What has your experience been as a mentor? What does your role involve? What do you like about being a mentor? What don’t you like about being a mentor? What challenges do you face as a mentor? How have you addressed the challenges so far?

***Umekumbana na mambo yapi kama mshauri? Jukumu lako ni nini? Nini ambacho unapenda kwa kuwa mshauri? Nini ambacho hupendi kwa kuwa mshauri? Ni changamoto zipi hukumbana nazo kama mshauri? Umetatua vipi changamoto hizi?***

1. Please tell me about any referrals you have made for AGYW in your group to other DREAMS interventions? How did you find the process of referring? Did you have sufficient tools etc to refer? Do you know if they attended? Do you know if they found it useful?

***Tafadhali niambie kuhusu kuelekeza msichana yoyote katika huduma moja hadi nyingine/zingine za DREAMS ambayo umehusisha wasichana waliobalehe na wanawake wadogo kutoka kikundi chako .***

1. Do you ever interact with other mentors? Please tell me about this.

***Je, umewahi kushirikiana na washauri wngine? Tafadhali niambie kuhusu shirika hizi?***

1. Do you ever interact with other community leaders or services providers in relation to DREAMS? Please tell me about this?

***Je, umewahi kushirikiana na viongozi wa jamii ama watoa huduma kuhusiana na mradi wa DREAMS? Tafadhali niambie kuhusu shirika hizi?***

1. I would like to learn more about your safe spaces group. What does your safe spaces group involve? What is going well in your group? What is not going well? What has been the effect of safe spaces on the girls who participate? What challenges are you facing with the group? What can be done to address these challenges?

***Ningependa kujua kuhusu kikundi cha safe spaces. Je, kikundi cha safe spaces kinahusika na nini? Nini kinachoendelea vyema katik kikundi chako? Nini ambacho hakiendelei vyema? Kikundi cha saf spaces kimekua na athari gani kwas wasichana ambao wanashiriki? Ni changamoto zipi ambazo unakabiliana nazo katika kikundi hiki? Nini kinachoweza fanywa ili kutatua changamoto hizi?***

1. What do you think about the DREAMS curriculum (guide/activity book/manual)? What do you like about it? Why? What don’t you like about it? Why? Are there any specific sessions that are particularly challenging? Why? What changes would you recommend to the curriculum (guide/activity book/manual)? Why and why not?

***Unafikiria vipi kuhusu mtaala (mwongozo) wa DREAMS? Nini unapenda kuihusu? Kwa nini? Nini ambacho hupendi kuhusu huu mtaala? Kwanini? Kuna vipindi haswa ambavyo vina changamoto? Kwanini? Ni mabadiliko yapi ambayo unaweza pendekeza kwa mtaala huo?***

1. Did you receive training to be a mentor? What did you like about the training? Do you think you received enough training to be able to do your role as a mentor well? What changes would you recommend to the training that you received to be a mentor?

***Kuna mafunzo yoyote ulipata ili kuwa mshauri? Nini ambacho ulipenda kuhusu mafunzo hayo? Je wafikiri ya kwamba ulipata masomo ya kutosha ili kuweza kufanya kazi yako vizuri kama mshauri?***

1. Do you ever interact with girls in your group outside of the safe spaces meeting hours? Describe these meeting. What types of help/ problems do girls come to you with?

***Je, umewahi kushirikiana na wasichana wa kikundi chako nje ya masaa ya kukutana katika safe spaces? Tafadhali nieleze kuhusu shirika hizi? Ni shida gani ambazo wasichana wanakuja nazo?***

1. Overall, how is the attendance in your group? Why do girls miss meetings? Have you ever been concerned about attendance at the meetings? Have you ever taken any steps or sought support from other DREAMS implementers to try and improve attendance?

***Kwa ujumla mahudhurio ya kikundi chako iko vipi? Kwanini wasichana wanakosa kushiriki kwa mikutano? Ushawahi kuwa na wasiwasi kwasababu ya mahudhurio ya mikutano? Ushawahi chukua hatua yeyote ama kuulizia usaidizi kutoka watu wengine ambao wanapatiana hizi huduma za DREAMS ili kuweza kuboresha mahudhurio?***

1. What do you expect the outcome will be for AGYW once they have completed the DREAMS programme?

***Una matarajio yapi kuhusu matokeo ya wasichana waliobalehe na wanawake wadogo mradi wa DREAMS uatakapokamilika?***

**Closing**

We have come to the end of the interview. Before we finish, is there anything else that you would like to add about what we have discussed?

***Tumefika tamati ya mahojiano yetu. Kabla tutamatishe, kunalo jambo lolote ambalo ungependa kuongezea kwa hayo tumejadiliana?***

Do you have any other feedback about the program? What other information would you like to know about the program?

***Kuna jambo lolote lingine unaweza taka kutuambia kuhusu huu mradi wa DREAMS?***

***Mambo mengine gani ungependa kujua kuhusu mradi wa DREAMS?***
