# Supplementary material for: Translating DREAMS into practice: Early lessons from implementation in six settings
Source: PLoS One. 2018 Dec 13;13(12):e0208243. doi: 10.1371/journal.pone.0208243 (PMC6292585; doi:10.1371/journal.pone.0208243)
Supplement: S3 File — (DOC) [file pone.0208243.s003.doc]

**S3 File. DREAMS Impact Evaluation, Focus Group Discussion Guide, South Africa (English)**

**FOCUS GROUP DISCUSSION GUIDE – COMMUNITY AND FAMILY MEMBERS**

# DISCUSSION GUIDE:

To understand how DREAMS is experienced by those it targets, those who deliver it and wider community members?

# COMMUNITY AGE AND GENDER GROUPS

Community and AGYW’s family members

#### **Time:**

#### Each activity may take about an hour. It is up to the group whether they want to take part in more than one activity.

**Objectives:**

- To investigate understanding and experience of DREAMS and its components and whether social norms and attitudes are being influenced, e.g., in what ways is DREAMS influencing social norms and attitudes within families and in the community, including among men, and barriers and facilitators to its operation
- To explore the experiences of AGYW’s families, parents, partners, and broader communities with DREAMS
- To explore what is the coverage and reach of components of DREAMS at different sites
- To explore how DREAMS is experienced and perceived by those it targets, those who deliver it and wider community members
- To find out what helps and hinders successful implementation of DREAMS
- To understand how implementation and experience of DREAMS is influenced by the differing contexts in which it is delivered, including the influence of other non-DREAMS activities in the target sites

#### **PARTICIPANTS:**

#### Conduct this at as many times as needed to cover all the exercises with 20 community and family members selected to represent AGYW’s age and families, partners and from different geographical areas in the community.

**MATERIALS:**

- Flipchart
- Marker Pens
- Scissors
- Masking Tape
- Sticky stuff
- Facilitator Notebook
- Pens,
- Pencils, crayons,
- Two copies of this guide
- Sheets of coloured A4 paper
- Information sheets
- Consent forms
- Digital Recorder
- AAA size batteries
- Digital camera
- A4 envelopes

Venue:

Community venue that offers privacy (free of distractions). These activities should NOT be conducted in the health centre.

**Seating arrangements:**

If possible arrange chairs in a semi-circle, *without* tables. Place recorder in centre of group.

Roles

Ideally 3 RAs (2 facilitating the exercises, 1 recording). RAs can alternate roles. Avoid one person facilitating everything e.g. if there is a discussion- one person should be writing on a flipchart, while the other facilitates the discussion

PREPARATIONS:

General

- Drinks and snacks for the participants and facilitators.
- Table of participant details (age and sex and participant number) pre-drawn on A4 paper to save time.
- All materials laid out and ready to use
- If a recorder is to be used this is checked, new batteries, spare batteries, ready to use

**What kind of place is this for young girls and boys?**

- Stick together 4 sheets of flipchart to make a large sheet

**Institutional mapping**

- Stick together 2 sheets of flipchart to make a large sheet

**Wealth, poverty and risk taking**

- Card/sheets of paper on which different characteristics can be written

**PROCEDURES:**

**PARTICIPANT CHARACTERISTICS**

As you wait for participants to arrive, one facilitator should **go** around the circle, and, for each participant, record the following: **Participant** **number,** **Age, Sex, and Marital Status, what they do for a living and length of time resident in site.**

Please note that this; should be done by the facilitator, not filled in by participants themselves. This should be done before the introduction, with the exception of latecomers. Names are not required.

**INTRODUCTION** [15 minutes]

We have selected all of you to represent your community here as we really value what you as members of this community can share with us about your experiences of DREAMS and its components in this community. Although we will not be providing money, we will be providing a drink and a snack during the discussion.

**ADMINISTER INFORMATION SHEET AND INFORMED CONSENT FORMS**

[EXPLAIN THE FOLLOWING]

Ask: Which language are you most comfortable using?

- This will be a one-three hour activity.
- We want it to be as participatory as possible. We want to hear your views. There are no right or wrong answers.
- Please feel free to give your ideas and also give a chance to everyone to speak.
- You may have different ideas from others – that’s okay – we want to hear them.
- Any points you feel uncomfortable about, please feel free not to respond.
- Your names will be used on the consent forms will be kept confidential – on the notes from this discussion we will use a number and when we write up the discussion, we never use people’s real names.
- We will be happy to answer any questions you have at the end of the discussion
- Please put your cell phones on silent if possible
- Is it ok that we record the discussion just when we are talking as one group?
- Is it ok if we start the discussion?

**RECORD THE TIME ACTIVITY STARTS, START THE RECORDER WHEN APPROPRIATE – I.E. WHEN GROUP IS DISCUSSING TOGETHER**

1. **What kind of place is this for young people? (15 minutes)**
2. Tell the group you want to find out more about the community
3. Give each participant a sheet of paper and ask them to draw a picture, or a symbol, or write some words – that shows something about their community. Allow 5 minutes
4. Ask everyone to stick their pictures on the big sheet and look at the pictures together.
5. Ask participants to explain their pictures if needed.
6. After discussing each of the pictures/phrases in turn, ask the participants for five key themes that summarise the community.

**Record the discussion**

1. **What is DREAMS and your experience with DREAMS?**

Introduce the exercise – ‘*We have heard some things about the community, now we want to think about different types of activities taking place in the community particularly the DREAMS interventions and how they are being experienced in the community and how they might affect social norms and attitudes within the families and in the community.*

**Theme 1: Perceptions of HIV risk in the community**

1. Ask participants to discuss what kind of risks young girls and women in their community are vulnerable to when it comes to HIV infection?

2. Which groups of girls are vulnerable to which risks? [Probe for age, type, socio-economic status, schooling levels, etc.].

**Theme 2: Knowledge and exposure to HIV prevention interventions including DREAMS (and DREAMS-like) interventions**

1. What kinds of interventions are already ongoing in their community to help young girls and women from contracting HIV? (Please probe for the organisations and the type of interventions being mentioned.
2. Ask about new interventions they know about [probe for approximate time when they started, if they are still ongoing and who is running them].
3. Now ask the participants about DREAMS - we want to focus on the DREAMS intervention from now on.
4. Ask do you know/ aware of any DREAMS interventions program? [Interviewer should have a list of interventions activities and probe using this only when the participants are not forthcoming].
5. Have you ever used (participated in/ been invited to participate in) or supported the intervention(s)?

**Theme 3: Experiences with DREAMS interventions/activities**

1. Ask each participant to describe their experience with the activities? Probe if they think it’s good or bad?
2. Ask those who never used some/parts of some activities, why not?
3. Have you ever used (participated in/ been invited to participate in) or supported the intervention(s).
4. If ever used or participated- probe- where, which organization and location?

**Theme 4: Perceptions of DREAMS interventions/activities**

1. Ask if they think the community is changing and if so, how?
2. Ask what they think the community feels about DREAMS? What is good and what is not so good?
3. **Pile - Sorting: Community Participatory Discussions (1 hour)**
4. Facilitate a discussion on `Experiences of interventions to reduce HIV infection in young people- DREAMS’ to generate ideas on what people think.
5. Write those ideas on sheets of paper
6. Lay all the pieces of paper out randomly on the floor or table so that each can be seen.
7. Give each participant 3 beans/ stone/ counters
8. Say: *Looking at all of these pieces of paper with your ideas which would you say are the 3 that are having the most impact on HIV prevention in this community? Place your beans on the one you think are working well. You can only put one bean per card.*
9. Allow enough time for everyone to place his or her beans. (Record where the beans are placed)
10. Give each participant another 3 beans/stones/counters of a different colour/type to the first 3.
11. Now say: *Which of these things do you think are not really working or having an impact on HIV prevention in this community? Again allow enough time for everyone to place their beans.*
12. Depending on where participants have put the beans - ask questions to understand the ranking. For example:
    - *I can see there is a lot of beans on this card- tell me why you think this is really working here?*
    - *Two of you have put your beans here- tell me more about this intervention?*
    - *What about this card with 4 beans on?*
    - *Etc*
13. At the end, ask participants*: What is the connection between targeted HIV interventions and the types of risks young girls and women are involved in?*

**Record the discussion**

1. **Institutional Mapping**
2. Ask participants to discuss about all the different services providers who offer DREAMS or health services for young people in the community. Free list all the providers on a flipchart.

[One facilitator to write the names of service providers on cards- so that there is one set of cards]

1. Ask the group to arrange all the cards in a way that shows the relationships between the institutions. For example - they could put organisations that work closely together in one group. Or an organisation that is an expert provider of a particular service on its own. [Don’t say too much- let the group decide how to arrange them]
2. Hear back from the whole group looking at the arranged cards. Ask questions to understand why the cards have been placed as they have.

Record the discussions.

**Probing more deeply:** now ask the following questions to try to find out more about community perceptions of service providers of DREAMS interventions. (if the topic has been covered in the group work, do not repeat the question):

- - - *Do you think there are enough HIV prevention service providers (probe for DREAMS) in this community? Are some offering all the same services?*
    - *Do you think AGYW health needs (in general) are covered by these institutions?*
    - *Do your local services give out the same messages about HIV prevention as you hear on the radio or TV? If not- what is different?*
    - *Have there ever been any Health campaigns and DREAMS mobilization in your community - for example staff from one of these institutions coming to talk to you in your home? If yes what was your experience of this*
    - *Who are the most important staff in these institutions (probe for different types of service providers)? What do you think about volunteer staff in the clinics - for example lay counsellors, treatment support workers, home-based carers?*

1. Now ask the whole group to choose the 3 strongest institutions. Move these cards to the centre of the card selection. Ask why they have chosen them.

**Record the discussion**

**CLOSING EACH SESSION**

**Comments/concerns of participants**

We have come to end of our discussion. Are there any questions you would like to ask us? [Make note of questions, answer what you can, refer others to appropriate people/organizations]

**Thanks**

Thank the participants: *Thank you all very much for your time. We have learnt much by listening to you.*
