# Supplementary material for: Translating DREAMS into practice: Early lessons from implementation in six settings
Source: PLoS One. 2018 Dec 13;13(12):e0208243. doi: 10.1371/journal.pone.0208243 (PMC6292585; doi:10.1371/journal.pone.0208243)
Supplement: S4 File — (DOC) [file pone.0208243.s004.doc]

**S4 File. DREAMS Impact Evaluation, Focus Group Discussion Guide, South Africa (Zulu)**

**UCWANINGO LOKUHLOLA UMTHELELA WOCWANINGO LWE-DREAMS**

**UMHLAHLANDLELA WEZINGXOXO ZEQOQO EZIGXILILE – AMALUNGA OMPHAKATHI NAWOMNDENI**

# UMHLAHLANDLELA WEZINGXOXO:

Ukuqonda ukuthi uhlelo lwe-DREAMS oluqondiswe kubo balubona lunjani, mayelana nalabo abalwenzayo kanye namalunga omphakathi wonkana.

# IMINYAKA YOBUDALA YAMALUNGA OMPHAKATHI KANYE NAMAQOQO OBULILI

Umphakathi kanye namantombazane amancane nabesifazane abasebancane-(AGYW) kanye namalunga emindeni yabo.

#### **Isikhathi:**

#### Umsebenzi ngamunye uyothatha cishe ihora. Kulele eqoqweni ekutheni lifuna ukuzibandakanya ezinhlelweni ezingaphezulu kolulodwa.

**Izinhloso:**

- Ukuphenya mayelana nokuqonda nalokho okuhlangabezwana nakho mayelana nohlelo lwe-DREAMS nezingxenye zalo nokuthi ingabe lunomthelela yini kwimigomo yenhlalakahle kanye nakwindlela abantu ababona ngayo izinto, isib. uhlelo lwe-DREAMS lunomthelela ngandlelani kwimigomo yenhlalakahle nakwindlela abantu ababona ngayo izinto emindenini nasemphakathini, kubandakanya abesilisa, nezithiyo kanye nalokho okwenza ukusebenza kwalo kube yimpumelelo.
- Ukuphenya ngalokho imindeni ye-AGYW, abazali, abalingani kanye nomphakathi wonkana abahlangabezana nakho mayelana nohlelo lwe-DREAMS.
- Ukuhlola ukuthi izingxenye zohlelo lwe-DREAMS sezihambe zaze zafinyelela kuphi kuzizinda ezihlukahlukene.
- Ukuhlola ukuthi uhlelo lwe-DREAMS oluqondiswe kubo balubona lunjani, mayelana nalabo abalulethayo kanye namalunga omphakathi wonkana.
- Ukuthola ukuthi yikuphi okwenza uhlelo lwe-DREAMS lusetshenziswe ngempumelelo kanye nalokho okwenza ukuthi lungasetshenziswa ngempumelelo.
- Ukuqonda ukuthi izindikimba ezihlukene zikulawula kanjani lokho okuhlangabezwane nakho kanye nokwethulwa kwezinhlelo zohlelo lwe-DREAMS kulezo zindawo lapho zethulwa khona, kubandakanya ukulawulwa ngezinye izinto ezenziwayo ezingaqondene nohlelo lwe-DREAMS kulezo zindawo olwethulwa kuzo.

#### **ABANGENELE UCWANINGO:**

#### Yenza lokhu izikhathi eziningi ngokudingekayo ukuze wenze yonke imisebenzi namalunga angama-20 omphakathi nemindeni aqokwe ukumela iminyaka yobudala ye-AGYW nemindeni, abalingani abavela ezindaweni ezihlukahlukene emphakathini.

**IZINTO EZIZODINGEKA:**

- I-flipchart
- Ama-Marker Pens
- Izikele
- I-Masking Tape
- Izinto zokunamathisela (sticky)
- Incwajana yomgqugquzeli yokuthatha amanothi
- Amapeni,
- Amapensela, amakhrayoni,
- Amakhophi amabili alo mhlahlandlela
- Amaphepha amibalabala ayi-A4
- Amaphepha olwazi
- Amaphephamvume
- Isiqophamazwi
- Amabhethri awubukhulu buka-AAA
- Ikhamera
- Izimvilophu ezibukhulu buka-A4

Indawo:

Indawo yomphakathi enendawo engasese (lapho ukuphazamiseka kungekho khona). Lezi zinhlelo AKUMELE ukuba zenzelwe esikhungweni sezempilo.

**Ukuhlala okuhleliwe:**

Uma kukwazeka, hlela izitulo zimise okwesigamu sesiyingi, *ngaphandle* kwamatafula. Beka isiqophamazwi phakathi nendawo eqoqweni.

Izindima

Ama-RA ama-3 alingene (ama-2 ahambisa imisebenzi, eyo-1 eqophayo). Ama-RA angashintshana ngezindima zawo. Gwema ukuthi umuntu oyedwa enze yonke into, isib. uma kukhona ingxoxo – umuntu oyedwa kumele kube nguye obhala kwi-flichart, ngesikhathi omunye ehambisa ingxoxo

AMALUNGISELELO:

Okujwayelekile

- Iziphuzo namasnekhi (ukudla okulula) kuhlinzekwe abangenele ucwaningo nabagqugquzeli.
- Ithebula lemininingwane yabangenele ucwaningo (iminyaka yobudala, ubulili kanye nenombolo yokuzibandakanya) lidwetshwe ephepheni le-A4 ngaphambi kokuthi kuqalwe ukuze kungachitheki isikhathi.
- Zonke izinto sezibekiwe sezilungele ukusetshenziswa
- Uma kuzosetshenziswa isiqophamazwi, kumele sihlolwe, amabhethri amasha, amabhethri ayisipele, alungele ukusebenza

**Yindawo enjani le mayelana namantombazane nabafana abasebancane?**

- Hlanganisa ndawonye amakhasi ama-4 e-flipchart ukuze wenze ikhasi elikhulu

**Ukwethulwa kwebalazwe lesikhungo**

- Hlanganisa amaphepha ama-2 e-flichart ndawonye ukuze wenze iphepha elikhulu

**Ubucebi, inhlupheko kanye nokuzibeka engozini**

- Amakhadi/amaphepha wekhasi okubhalwa kulo izimpawu ezihlukahlukene

**IZINQUBO:**

**IZIMPAWU ZABANGENELE UCWANINGO**

Ngesikhathi usulinde ukufika kwabangenele ucwaningo, umgqugquzeli oyedwa kumele **azungeze** isiyingi, bese kuthi mayelana nongenele ucwaningo ngamunye, kubhalwe lokhu okulandelayo: **Inombolo yongenele ucwaningo, Iminyaka Yobudala, Ubulili, Nesimo Somshado, lokho abaziphilisa ngakho kanye nobude besikhathi besesikhungwini.**

Sicela wazi lokhu ukuthi lokhu kumele kwenziwe ngumgqugquzeli, kungagcwaliswa ngabangenele ucwaningo. Lokhu kumele kwenziwe ngaphambi kwesingeniso, ngaphandle kwalabo abazofika emva kwesikhathi. Amagama awadingeki.

**ISINGENISO** [imizuzu engu-15]

Siqoke nina nonke ukuthi nizomela umphakathi wenu lapha njengoba sikubona kubalulekile lokho enizositshela khona mayelana nalokho enihlangabezane nakho mayelana nohlelo lwe-DREAMS nezingxenye zalo kulomphakathi njengamalunga awo. Nakuba singeke sikunike imali, sizokunika isiphuzo nesinekhi/ukudla okulula ngesikhathi kuqhubeka ingxoxo.

**LAWULA IKHASI LOLWAZI LONGENELE UCWANINGO KANYE NEPHEPHAMVUME ANOLWAZI**

[CHAZA OKULANDELAYO]

Buza: Yiluphi ulimi enikhululeke kakhulu ukulisebenzisa?

- Lolu hlelo luzothatha isikhathi kusukela ehoreni elilodwa kuya kwamathathu.
- Sifuna ukuthi nidlale izindima zenu ngokusemandleni. Sifuna ukuzwa ngemibono yenu. Azikho izimpendulo ezishaya khona noma ezingashayi khona.
- Sicela nikhululeke ekusinikeni imibono yenu futhi ninikezane nethuba lokuthi wonke umuntu akwazi ukukhuluma.
- Imibono yenu ingehluka kweyabanye – lokho kulungile – sifuna ukuyizwa.
- Uma kukhona iphuzu ongakhululeki ngalo, sicela ukhululeke ungaphenduli.
- Amagama enu azosetshenziswa kwiphepha mvume futhi azogcinwa eyimfihlo – emanothini ale ngxoxo kuzosetshenziswa inombolo nangesikhathi sibhala phansi le ngxoxo, ngeke sisebenzise amagama angempela abantu.
- Sizokuthokozela ukuphendula noma iyiphi imibuzo enizoba nayo ekupheleni kwale ngxoxo
- Sicela nenze omakhalekhukhwini benu bangakhali baphimisele uma kwenzeka
- Ingabe kulungile yini ukuthi sirekhode ingxoxo ngesikhathi sikhuluma siyiqoqo elilodwa?
- Ingabe sekulungile yini ukuthi siqale ngengxoxo?

**BHALA ISIKHATHI SOKUQALA KOHLELO, VULA ISIQOPHAMAZWI UMA SEKUFANELE – NJENGOKUTHI UMA IQEMBU LIXOXISANA LODWA**

1. **Hlobo luni lwendawo lolumayelana nabantu abasebancane? (imizuzu engu-15)**
2. Tshela iqoqo ukuthi ufuna ukuthola kabanzi ngomphakathi
3. Nikeza ongenele ucwaningo ngamunye ilekhasi phepha bese ubacela ukuba badwebe isithombe, noma uphawu, noma babhale amagama athile – okukhombisa okuthile mayelana nomphakathi wabo. Banikeze imizuzu engu--5
4. Cela ukuthi wonke umuntu anamathisele isithombe sakhe ephepheni elikhulu bese bonke bebheka ezithombeni.
5. Cela abangenele ucwaningo ukuba bachaze ngezithombe zabo uma kudingeka.
6. Emva kokuxoxisana ngezithombe ngazinye/ngamagama ngokushintshana, cela abangenele ucwaningo ukuba bakunikeze izindikimba ezinhlanu ezibalulekile ezifingqa isimo somphakathi.

**Qopha ingxoxo**

1. **Luyini uhlelo lwe-DREAMS kanye nalokho ohlangabezane nakho ohlelweni lwe-DREAMS?**

*Ngenisa umsebenzi – ‘sizwe ngezinto ezithile mayelana nomphakathi, manje sifuna ukucabanga mayelana nezinhlobo ezihlukahlukene zezinhlelo ezenziwa emphakathini, ikakhulukazi izinhlelo zokungenelela zohlelo lwe-DREAMS futhi zenziwa emphakathini futhi kungenzeka zibe nemithelela kwimigomo yenhlalakahle nakwindlela abantu ababona ngayo izinto emindenini nasemphakathini.*

**Ingqikithithi yokuqala: Imicabango ngokuba sengcupheni yegciwane lengculaza emphakathini.**

1. Cela abangenele ucwaningo ukuthi baxoxisane ngezinhlobo ngezingozi ezibeka amantombazane nabesifazane engcupheni yokutheleleka ngesandulela ngculazi-(HIV)?
2. Yimaphi amaqoqo amantombazane asengcupheni futhi kuziphi izingozi? [buzisisa ngeminyaka yobudala, uhlobo, izimo zomnotho womphakathi, amazinga emfundo, njl.]

**Ingqikithi yesibili: Ulwazi ngemingenelelo yokuvikela yakwa DREAMS**

1. Yiziphi izinhlobo zezinhlelo zokungenelela eziqhubekayo emphakathini wabo ngenhloso yokusiza amantombazane nabesifazane abasebancane ukuba bangatheleleki ngesandulela ngculazi (-HIV)? [Sicela ubuzisise mayelana nezinhlangano kanye nezinhlobo zezinhlelo ezishiwoyo]
2. Buza mayelana nezinhlelo zokungenelela ezintsha abazaziyo [buzisisa ngezikhathi eziqale ngazo, uma zisaqhubeka, zenziwa ngubani]
3. Manje buza abangenele ucwaningo ngohlelo lwe-DREAMS – ukusukela manje sifuna ukugxila ezinhlelweni zohlelo lwe-DREAMS.
4. Buza ukuthi ingabe bayazi yini mayelana nanoma iluphi uhlelo lokungenelela lwe-DREAMS? [Lowo obuzayo imibuzo kumele abe nohlu lwezinhlelo zokungenelela futhi buza usebenzisa loluhlu kuphela uma abangenele ucwaningo bengavuleleki]
5. Ingabe uke wazisebenzisa noma weseka izinhlelo zokungenelela (wazibandakanya kuzo/wamenywa ukuba uzobandakanye kuzo)?

**Ingqikithi yesithathu: Isipiliyoni mayelana nezinhlelo ze DREAMS**

1. Cela ongenele ucwaningo ngamunye ukuba achaze ngalokho ahlangabezane nakho mayelana nezinhlelo?
2. Buza labo abangakaze bazisebenzise izingxenye zezinhlelo ze DREAMS ukuthi kungani?
3. Wake wazisebenzisa yini (wazimbandakanya/ wamenywa ukubamba iqhaza) noma weseka u/imingenelelo ye DREAMS.
4. Uma bake bazisebenzisa noma babandakanyeka kuzo – buzisisa mayelana nokuthi – kuphi, iyiphi inhlangano kanye nendawo?

**Ingqikithi yesine: Imicabango yemingenelelo ye DREAMS**

1. Buza ukuthi ingabe bacabanga ukuthi lukhona yini ushintsho emphakathini, uma lukhona lwenzeke kanjani?
2. Buza ukuthi bacabanga ukuthi umphakathi uzizwa kanjani ngohlelo lwe-DREAMS? Yikuphi okuhle nalokho okubi?
3. **Qoqela ndawonye – Uhlele ngononina: Izingxoxo Lapho Umphakathi Uzibandakanya (ihora elilo-1)**
4. Qala ingxoxo mayelana ‘Nezinhlelo zokungenelela zokwehlisa izinga lokutheleleka ngesandulela Ngculazi-HIV kubantu abasebancane – i-DREAMS’ ukuze uthole imibono mayelana nokuthi abantu bacabangani.
5. Bhala leyo mibono emaphepheni ekhasi
6. Beka wonke amaphepha ngendlela engahlelekile phansi noma etafuleni ukuze abonakale.
7. Nikeza ongenele ucwaningo ngamunye ubhontshisi/itshe/amakhawunta oku-3
8. Yithi: *Uma nibheka kuwo wonke amaphepha anemibono yenu, ningasho yini ukuthi yikuphi oku-3 okunemithelela emikhulu ekuvimbeleni i-isandulela ngculazi HIV kulo mphakathi? Bekani ubhontshisi wenu kulokho enicabanga ukuthi kusebenza kahle. Uvumeleke ukuthi ubeke ubhontshisi owodwa kuphela ekhadini ngalinye.*
9. Banikeze isikhathi esanele sokuthi wonke umuntu abeke ubhontshisi wakhe (Bhala ukuthi ubhontshisi ubekwa kuphi)
10. Phinda unikeze ongenele ucwaningo ngamunye omunye ubhontshisi/amatshe/okokubala oku-3 okunemibala ehlukile/uhlobo oluhlukile kulokhuya oku-3 kokuqala
11. Manje yithi: *Yikuphi kulokho ocabanga ukuthi akusebenzi noma akunawo umthelela ekuvimbeleni i-sandulela ngculazi HIV kulo mphakathi? Phinda unikeze wonke umuntu isikhathi esenele sokubeka obhontshisi babo.*
12. Ngokuya ngendawo lapho abangenele ucwaningo bebeke khona obhontshisi babo – buza imibuzo ukuze uqonde ngamazinga noma ngokuklelisa. Isibonelo:
    - *Ngiyabona ukuthi kukhona obhontshisi abaningi kuleli khadi – ngitsheleni-ke ukuthi yingani nicabanga ukuthi lokhu kuyasebenza ngempela lapha?*
    - *Ababili benu babeke obhontshisi babo lapha – ngitsheleni ngokuthe xaxa mayelana naloluhlelo lokungenelela?*
    - *Kwenzekani ngaleli khadi okubekwe obhontshisi aba-4 kulo?*
    - *Njl.*
13. Ekugcineni, buza abangenele ucwaningo lokhu: *Yikuphi ukuxhumana okukhona phakathi kwezinhlelo zokungenelela zeSandulela Ngculazi(-HIV) kanye nezinhlobo zezingozi amantombazane nabesifazane abasebancane ababandakanyeka kukho.*

**Bhala ingxoxo**

1. **Ukwethula Ibalazwe Lesikhungo**
2. Cela abangenele ucwaningo ukuba baxoxisane ngabo bonke abanikezeli bezinsiza abanikezela ngohlelo lwe-DREAMS noma ngezinsiza zezempilo mayelana nabantu abasebancane emphakathini. Yenza uhlu lwabo bonke abahlinzeki kwi-flipchart.

[Umgqugquzeli oyedwa uzobhala amagama abanikezeli bezinsiza emakhadini – ukuze kube khona isethi elilodwa lamakhadi]

1. Cela iqoqo ukuthi lihlele wonke amakhadi ngendlela yokuthi akhombise ubudlelwane phakathi kwezikhungo. Isibonelo – bangabeka izinhlangano esisebenzisanayo eqoqweni elilodwa. Noma babeke inhlangano engumhlinzeki ongungoti kwizinsiza ethile yodwa. [Ungakhulumi kakhulu – yekela iqoqo lizithathele isinqumo ukuthi lifuna ukuwahlela kanjani]
2. Lalela iqoqo lonke elibheke emakhadini ahleliwe. Buza imibuzo ukuze uqonde ukuthi yingani amakhadi ebekwe ngale ndlela abekwe ngayo.

Bhala izingxoxo.

**Buzisisa ngokuthe xaxa**: manje buza le mibuzo elandelayo ngenhloso yokuthola ngokuthe xaxa mayelana nendlela umphakathi obona ngayo abanikezeli bezinsiza zezinhlelo zokungenelela zohlelo lwe-DREAMS. (uma sekukhulunyiwe ngesihloko emsebenzini weqembu, ungawuphindi umbuzo):

- - - *Ingabe ucabanga ukuthi abanikezeli bezinsiza ezimayelana nokuvimbela i-Sandulela Ngculazi(HIV )benele yini (buzisia ngalawo ohlelo lwe-DREAMS) kulomphakathi? Ingabe ucabanga ukuthi baningi kakhulu? Ingabe abanye abanikeza ngazo zonke izinsiza ezifanayo?*
    - *Ingabe izidingo (jikelele) zezempilo ze-AGYW zibhekelelwe kulezizikhungo?*
    - *Ingabe izinsiza zendawo yakini zinikeza umyalezo ofanayo yini nalowo owuzwa emsakazweni noma ku-TV mayelana nokuvinjelwa kwe-Sandulela Ngculazi (HIV)? Uma kungenjalo, kuhluka kuphi?*
    - *Ingabe ibe yaba khona yini imikhankaso Yezempilo kanye nokukwaziswa ngohlelo lwe-DREAMS emphakathini wakho – isibonelo, ukuza kwabasebenzi ekhaya lakho abavela kwesinye salezi zikhungo ngenhloso yokuzoxoxisana nawe? Uma kunjalo, wakubona kunjani lokhu ?*
    - *Ngobani abasebenzi ababaluleke kakhulu kulezizikhungo (buzisisa mayelana nezinhlobo ezahlukene zabanikezeli bezinsiza)? Ucabanga kanjani mayelana namavolontiya emitholampilo – isibonelo abeluleki abangakufundele lokho, indlela abaphathana ngayo, abasebenzi abesekayo, abanakekeli abaya emakhaya?*

1. Manje cela lonke iqoqo ukuba likhethe izikhungo ezinamandla ezi-3 Beka lawa makhadi phakathi nendawo lapho kukhethwa khona amakhadi. Buza ukuthi yingani bekhethe wona.

**Bhala ingxoxo**

**UKUVALA LOWO NALOWO MHLANGANO**

**Ukuphawula/okukhathaza abangenele ucwaningo**

Sesifike ekugcineni kwengxoxo yethu. Ingabe kunanoma yimiphi imibuzo ongathanda ukusibuza yona? [Khumbula imibuzo, phendula leyo okwaziyo ukuyiphendula, dlulisela eminye kubantu/kuzinhlangano ezifanele]

**Siyabonga**

Bonga ongenele ucwaningo: *Siyanibonga nonke ngesikhathi senu. Sifunde okuningi ngokunilalela.*
