# Supplementary material for: Translating DREAMS into practice: Early lessons from implementation in six settings
Source: PLoS One. 2018 Dec 13;13(12):e0208243. doi: 10.1371/journal.pone.0208243 (PMC6292585; doi:10.1371/journal.pone.0208243)
Supplement: S5 File — (DOCX) [file pone.0208243.s005.docx]

**S5 File. DREAMS Impact Evaluation, Key Informant Interview Guide, South Africa**

**Introduction**

1. **How long have you lived in this community?**  *Were you born here? How did you come to stay here?*
2. **Can you tell me about your work here?** [*If applicable] How long have you been doing this job? Do you enjoy your work?*

**SECTION 1: WITH DREAMS Implementers**

**THEME 1: Experiences implementing DREAMS (and DREAMS-like activities)**

1. **Can you please tell me what DREAMS related activities your organisation is involved in?** *Probe- how you got involved in DREAMS? Ask about targets and target groups , Ask how frequent/often the activities are done. Ask if any of their work deals with PrEP specifically if they have heard of PrEP/pills that people who are not infected with HIV can take to prevent HIV infection.*
2. **Can you please tell me how you recruit the target groups?** *Probe – how they identify them, approach them and invite them to participate [Only if they have heard of PrEP above]: Probe as to their opinions as to who should use PrEP, whether they would be willing to prescribe/refer AGYW & ABYM to get PrEP should it be approved for adolescents, how useful they think PrEP is for AGYW, etc.*
3. **When did the roll-out of the DREAMS activities start?**
4. **What preparation work was done before the roll-out?** *Probe - who are the* *staff working on the different activities, their qualification and training?*
5. **What is the coverage and reach of components of DREAMS at different sites?** *Probe- in terms of geographical area covered, wards, isigodi or neighbourhoods – whatever boundary demarcations they are using. Ask how the coverage is/was determined was it by need or other criteria used.*
6. **Can you tell me how you retain participants, how they exit the programs and what happens to them afterwards?** *Probe about drop-outs, those who are not within target group but willing to participate in DREAMS activities etc*
7. **Can you please tell me about the reporting processes?** *Probe financial, monitoring and evaluation etc*

**THEME 2: Perceptions with the DREAMS program**

1. **How do you think DREAMS is experienced by those it targets e.g the adolescent girls and young women, their families and communities?** *Probe whether they think it is a good or bad program - for them and or for those benefiting from it?*
2. **Do you have a sense of whether things have changed since you started DREAMS? If so - how and why?** *Probe – is there any noticed improvement or lack of from those receiving DREAMS, from the community at large or any other changes they may have noticed?*

**THEME 3: Barriers and facilitators of implementing DREAMS**

1. **What are the factors that have been helping and or hindering the successful implementation of DREAMS in your organisation, community and or facility?** *Only probe if the respondent doesn’t respond - for political factors - different government departments, financial factors, lack of/support from families and the young girls, schools, etc*
