# Supplementary material for: Translating DREAMS into practice: Early lessons from implementation in six settings
Source: PLoS One. 2018 Dec 13;13(12):e0208243. doi: 10.1371/journal.pone.0208243 (PMC6292585; doi:10.1371/journal.pone.0208243)
Supplement: S6 File — (DOC) [file pone.0208243.s006.doc]

**S6 File. DREAMS Impact Evaluation, In-depth Interview guide for qualitative cohort, South Africa (English)**

**Qualitative longitudinal study with young people - in-depth interview guide for young men and women**

**Participants**

**Time:** 1 -1.5 hours

We will trace young women’s experiences of and ‘journeys’ through DREAMS, including barriers and facilitators to what works in practice, starting with an in-depth interview followed by up to 10 brief and informal chats to gain an in-depth understanding of the DREAMS program experiences of a small cohort of 20 AGYW and 20 ABYM aged 15-24.

The in-depth interview will explore their experience to date and their expectations of sexual and reproductive health, and HIV treatment and prevention services and other DREAMS type intervention

**Objectives:**

- To trace young women’s and men’s experiences of and ‘journeys’ through DREAMS, including barriers and facilitators to what works in practice,
- To explore their experience and expectations of sexual and reproductive health, and HIV treatment and prevention services and other DREAMS type intervention

**Participants:**

Identify a small cohort of 20 AGYW and 20 ABYM aged 15-24 receiving any DREAMS activities in the Hlabisa sub-district. Provide them with a choice of regular informal, interviewer initiated face-to-face or telephone interviews to describe their experience of the DREAMS roll out and factors that helped and hindered their use of services.

**Venue:** Private spaces; allow participant to identify a safe space.

**Preparations:**

In-depth interview guide, notebook, pencil/pen, recorder, information sheets and consent forms. Interview should be carried out by one researcher.

**Introduction**

Explanation of research: Thank you for agreeing to talk to us.

Administer information sheet

- **Collect participant details**
- **Administer informed consent**
- **Explain that this interview will take no longer than an hour and will be recorded for research use only**

Information to collect to go at the top of the transcript:

**Participant details:**

Name of Interviewer(s): ____________________________

Date: ______________

Location [District, Province]: ­­­­­­­­­­______________________

Interviewee’s Name: _____________________

Position in Community______________________________

Age [in years]: _____________

Sex [circle one]: FEMALE MALE

Tribe: ________________ Nationality: ________________

This is an in-depth interview and below is the topic guide with some open-ended questions that you can use. But the key is to probe and gain depth of information around the young persons experiences and expectations.

**Questions/Themes**

**Personal**

1. **How long have you lived in this community?**  *Were you born here? How did you come to stay here?*
2. **Can you tell me about yourself*?*** *[as a way to establish rapport and get to know the participant] Let the participant tell you anything about themselves – probe for age, educational status whether in/out of school, whether they are working, about their family and any other general information.*

**Socio-demographic details**

**If not already mentioned, ask:**

1. **How old are you? When were you born?**
2. **Gender**
3. **Which religion do you belong to?** Probe which church do you go to?
4. **If below 18 years, ask if their parents are alive?** Probe who they live with and who is taking care of them. Also ask how many children are in their household and who is providing for the household financially?
5. **What is your level of education?** Probe - How far did you reach in completing your studies if you are no longer in school? What are the reasons for not being in school if you are supposed to be in school? If in school, ask what grade they are in.
6. **Do you have a DREAMS identifier? It could be a reference number, ID or code. [South Africa], can you share it?**

**Theme 1: Experience and expectation of healthcare**

Have you used any healthcare facility over the past year? What has been you experience? What were the good things? What were the bad things? What could be better?

**Theme 2: Experience and expectation of HIV testing**

Have you ever tested for HIV? If they say yes then probe around in which setting and what was their experience? What could be done better? Would they recommend that setting and if not why not? Do they know where to test? Would they feel comfortable to test there and if not why not? If they haven’t tested explore the reasons not and what sorts of things would make it easier to test? Do not probe the results, but if they volunteer that they are positive, probe their experience of HIV care?

**Theme 3: Experience and expectation of reproductive health and sexual health care**

Start with an open-ended question around their fertility desires? Do they ever want children? What do they understand about fertility and what impacts on it? What do they envisage as safe pregnancy? Ask where they received their information around fertility and sex and what do they feel about the way in which they were taught about it?

With women go onto probe around their understanding and experience of contraception? Probe if they are using any contraception and what their experiences of the healthcare was? If not explore whether they know where to go and if they would feel comfortable asking for contraception?

For men ask around their awareness of voluntary male medical circumcision and again would they consider it and if so why and if not why not? If they have experienced it then what was their experience?

**Theme 4: Experiences with the wider DREAMS and DREAMS type interventions– (these will be themes that will also be explored in the follow-up informal conversations)**

Introducing DREAMS and exploring what they have already heard about DREAMS. This will a probe to repeated over the two years during the informal discussions.

**Have you heard of a program called DREAMS?** (probe using the names of the organisations that are working in the area? How did you hear of the program called ‘DREAMS’? Explore how and why they became involved? What do they think about it? What is good? What is bad? What they would change? how you got to be involved? Who approached you? How did they know about you? Why were you chosen and what steps did they take?

**Probe around which DREAMS or DREAMS type intervention are you participating in?** If they are not sure –ask which organisation they are receiving the intervention from and what activities they are participating in. Over time probe around which programmes they started participating and which ones they stopped? Why they stopped? What things that could be done better? Things they would like but are not available?

Here are a list of some of the things that may be probed directly- perhaps using the organisation logo or visual as a cue:

1. *Safe Spaces for girls/young women*

*[Local name for the Safe Spaces program]*

1. *Mentor programme*

*[Local name for the Mentorship program]*

1. *Social Assets programs*
2. *School-based Activities- Vhutsilo and TALC*
3. HIV testing and counselling services
4. *Condom provision - at health facilities, in the community*
5. *Counselling on contraception / family planning*
6. *Provision of contraceptive methods / supplies*
7. *Services after an experience of violence (including sexual, physical or emotional)*
8. *Adolescent-friendly services like happy hour, priority queues for school children in uniform, etc)*
9. *HIV/STI Prevention*
10. *Pre-exposure prophylaxis (PrEP) (Tablets that women who do not have HIV can take to prevent becoming infected)*
11. *Post-exposure prophylaxis (PEP) (Drugs that can be taken to prevent HIV infection after possible exposure to the virus, including post-violence)*
12. *STI screening and treatment*
13. *For males - Male circumcision (voluntary medical circumcision)*

**Are your parents/guardians/caregiver involved/support you in receiving these sorts of intervention?** *Probe for reasons why/why not – is it because of the sensitivity eg condom use or contraception? Explain if this is affecting/will affect continued access/utilisation?*

**Other probes you can use are do you think this intervention/being on the DREAMS programme are beneficial?** *[Let the participant talk freely and uninterrupted and probe them to explain more if they mention any of the aspects regarding education, health and HIV/AIDS]*

**Closing**

Thank the participant for their time and sharing their information. Summarise the work that has been done by the research team so far and explain what will follow in terms of up to 10 future contacts over the next two years. Make a plan to arrange the next follow-up conversation, either by telephone or face to face. And take a variety of contact details

Ask if they have any questions

Thank them for their time and offer refreshments and transport reimbursement if they travelled
