# Supplementary material for: Translating DREAMS into practice: Early lessons from implementation in six settings
Source: PLoS One. 2018 Dec 13;13(12):e0208243. doi: 10.1371/journal.pone.0208243 (PMC6292585; doi:10.1371/journal.pone.0208243)
Supplement: S7 File — (DOC) [file pone.0208243.s007.doc]

**S7 File. DREAMS Impact Evaluation, In-depth Interview guide for qualitative cohort, South Africa (Zulu)**

**UCWANINGO LOKUHLOLA UMTHELELA WOHLELO LWE-DREAMS**

**Ucwaningo Olumayelana Nezinganhle nabantu abasebancane – umhlahlandlela wesigcawu semibuzo esijulile mayelana nabesilisa nabesifazane abasebancane**

**Abazibandakanyayo**

**Isikhathi:** Amahora angu-1 -1.5

Sizophenya ngalokho abesifazane abasebancane abahlangabezane nakho kanye ‘nezinkambo zabo’ mayelana nohlelo lwe-DREAMS, kubandakanya izithiyo kanye nalokho okusebenzayo ekwenzeni kube lula, sizoqala ngesigcawu esijulile semibuzo bese kulandela izingxoxo ezimfushane ezingahlelekile ezingu-10 okwenziwa ngenhloso yokuthola ukuqonda kabanzi ngalokho iqembu elincane le-AGYW elinamalungu angu--20 kanye nele-ABYM elinamalunga angu--20 aneminyaka yobudala ephakathi kwengu-15 kuya ku 24.

Isigcawu semibuzo esijulile sizophenya lokho abahlangabezane nakho kuze kube manje kanye nalokho abakulindele mayelana nezempilo zezocansi nokuzala, kanye nezinkonzo zokwelaphelwa i-Sandulela ngculazi kanye nawokuyivimbela kanye nolunye uhlobo lohlelo lwe-DREAMS

**Izinhloso:**

- Ukuphenya ngalokho abesifazane abasebancane abahlangabezane nakho kanye ‘nezinkambo zabo’ mayelana nohlelo lwe-DREAMS, kubandakanya izithiyo kanye nalokho okusebenzayo ekwenzeni kube lula,
- Ukuphenya ngalokho abahlangabezane nakho kanye nalokho abakulindele mayelana nezempilo zezocansi nokuzala, kanye namasevisi okwelaphelwa i-Sandulela ngculazi kanye nawokuyivimvela kanye nolunye uhlobo lohlelo lwe-DREAMS

**Abangenele ucwaningo:**

Khomba iqembu elincane le-AGYW elinamalunga angu--20 kanye nele-ABYM elinamalunga angu--20 aseminyakeni ephakathi kwengu-15 kuya kwangu--20 athola noma yiziphi izinhlelo zohlelo lwe-DREAMS esifundeni esincane sakwaHlabisa. Bazise ngendlela abangayikhetha phakathi kwesigcawu semibuzo esingahlelekile esenziwa ubuso nobuso esiqalwa yliowo obuza imibuzo kanye nesigcawu semibuzo esenziwa ngocingo ngenhloso yokuchaza lokho abahlangabezane nakho ngokwenziwa kwalolucwaningo lwe- DREAMS kanye nezimo ezisizile neziphazamise ukusebenzisa kwabo izinkonzo.

**Indawo:** Izindawo ezingasese; vumela ukuthi ongenele ucwaningo ekhombe indawo ephephile.

**Amalungiselelo:**

Umhlahlandlela ojulile wesigcawu semibuzo, i-notebook, ipensela/umsizi, isiqophamazwi, amakhasi olwazi kanye namafomu olwazi. Isigcawu semibuzo kumele senziwe ngumcwaningo oyedwa.

**Isingeniso**

Incazelo yocwaningo: Siyabonga ngokuvuma ukukhuluma nathi.

Lawula ikhasi lolwazi

- **Qoqa imininingwane yongenele ucwaningo**
- **Lawula imvume enikezwa emva kokuthola kolwazi**
- **Chaza ukuthi lesi sigcawu semibuzo angeke sithathe isikhathi esingaphezu kwehora futhi sizo qoshwa mayelana nocwaningo kuphela**

Ulwazi okumele luqoqwe kumele lube ngenhla kombhalo othathelwe kokukhulunyiwe (transcript):

**Imininingwane yozibandakanya ocwaningweni:**

Igama lalowo obuzayo: ____________________________

Usuku: ______________

Indawo [Isifunda, Isifundazwe]: ______________________

Igama lalowo ophendula imibuzo: _____________________

Isikhundla Emphakathini______________________________

Ubudala [ngeminyaka]: _____________

Ubulili [kokelezela eyodwa]: OWESIFAZANE OWESILISA

Isizwe: ________________ Ubuzwe: ________________

Lesi yisigcawu esijulile semibuzo kanti lapha ngezansi kukhona umhlahlandlela wezihloko ezinemibuzo evulelekile ongayisebenzisa. Kodwa okusemqoka ukuphenya nokuthola ulwazi olunzulu mayelana nalokho abantu abasebancane abahlangabezana nakho kanye nalokho abakulindele.

**Imibuzo/Izindikimba**

**Okuqondene nawe siqu**

1. **Unesikhathi esingakanani uhlala kulomphakathi?** *Ingabe wazalelwa lapha? Uze kanjani lapha?*
2. **Ungangitshela ngawe?** *[njengendlela yokusungula ubudlelwane neyokwazana nalowo ongenele ucwaningo] Yithi ongenele ucwaningo akutshele noma yini emayelana naye – phenya ngeminyaka yakhe yobudala, isimo sakhe semfundo, ukuthi ingabe uyafunda noma akafundi, ingabe uyasebenza, mayelana nomndeni wakhe kanye nanoma yiluphi olunye ulwazi oluvamile.*

**Imininingwane mayelana nezimo zenhlalo yabantu**

**Uma kungakashiwo lokhu, buza:**

1. **Uneminyaka yobudala emingaki? Wazalelwa kuphi?**
2. **Ubulili**
3. **Ungowayiphi inkolo?** Thola ukuthi usontaphi?
4. **Uma eneminyaka engaphansi kwengu-18, buza ukuthi ingabe abazali bakhe basaphila yini?** Thola ukuthi uhlala nobani futhi ngubani omnakekelayo. Buza nokuthi zingaki izingane emndenini wakubo nokuthi ngubani ongenisa imali ekhaya?
5. **Yiliphi izinga lakho lokufunda?** Thola - Ufunde wagcinaphi uma ungasafundi? Yiziphi izizathu ezenza ukuthi ungafundi kodwa ube ufanele ukuba sesikoleni? Uma efunda, mbuze ukuthi ukweliphi ibanga.
6. **Ingabe unakho ohlonzwa ngakho ohlelweni lwe-DREAMS? Kungaba inombolo yeriferensi, umazisi (ID) noma ikhodi. [INingizimu Afrika], ungasitshela ngalokhu?**

**Indikimba 1: Lokho ohlangabezane nakho noma okulindele mayelana nokunakekelwa ngokwezempilo**

Ingabe uke wasebenzisa noma yiluphi uhlelo lokunakekelwa ngokwezempilo esikhathi esingunyaka esedlule? Uhlangabezane nani? Yikuphi okwakukuhle? Yikuphi okwakukubi? Yikuphi okungenziwa ngcono?

**Indikimba 2: Ohlangabezane nakho nalokho okulindele mayelana nokuhlolelwa i-Sandulela ngculazi**

Ingabe uke wahlolelwa i-Sandulela ngculazi? Uma impendulo yakhe ithi yebo, thola ukuthi wahlolelwa kuphi noma ngaphansi kwasiphi isimo futhi yikuphi ahlangabezana nakho? Yikuphi okungenziwa ngcono? Angasincomela omunye umuntu yini leso simo yena ahlolwa ngaphansi kwaso, uma impendulo ithi cha, yingani engeke akwenze lokho? Ingabe uyazi nje ukuthi kumele ayohlolelwa kuphi? Angazizwa ekhululekile yini uma ehlolelwa lapho, uma kungenjalo, yingani? Uma ubengakahlolwa, thola izizathu zalokho nokuthi yiziphi izinto ezingenza ukuthi kube lula kuye ukuyohlolwa? Ungabuzi ngemiphumela, kodwa uma ezisholo yena ukuthi une-Sandulela ngculazi, thola mayelana nokunakekelwa kwakhe maqondana ne-Sandulela ngculazi?

**Indikimba 3: Okuhlangabezwana nakho nalokho okulindelwe mayelana nonakekelo lwezempilo maqondana nokuzala nezocansi**

Qala ngokubuza umbuzo ovulelekile mayelana nezifiso zakhe ezimaqondana nesimo senzalo? Ingabe bayazifuna izingane? Yikuphi abakuqonda mayelana nesimo senzalo nokuthi yini enomthelela kuso? Yikuphi akubona njengokukhulelwa okuphephile? Mbuze ukuthi baluthola kuphi ulwazi mayelana nesimo senzalo nezocansi kanye nokuthi bazizwa kanjani ngendlela abafundiswa ngayo?

Mayelana nabesifazane, thola mayelana nokuqonda kwabo kanye nalokho abahlangabezane nakho mayelana nezindlela ezivimbela inzalo? Thola ukuthi ingabe bayayisebenzisa yini indlela yokuvimbela inzalo nokuthi yikuphi abahlangabezana nakho mayelana nokunakekelwa ngokwezempilo? Uma kungenjalo, thola ukuthi ingabe bayazi yini ukuthi kumele baye kuphi lapho bengazizwa bekhululekile ukuba bacele mayelana nezindlela zokuvimbela inzalo?

Mayelana nabesilisa, buza ukuthi ingabe bayazi yini mayelana nokusokwa kwisizinda sezempilo nokuthi bangaya yini nokuthi zizathu zini ezingabenza ukuthi baye noma bangayi? Uma sebeke baya, yikuphi abahlangabezana nakho?

**Indikimba 4: Lokho okuhlangabezwana nakho mayelana nohlelo lwe-DREAMS kanye nezinhlelo nohlobo lokungenelela kohlelo lwe-DREAMS ngobubanzi – (lezi yizindikimba ezizobhekwa kabanzi ezingxoxweni zokulandelela ezingahlelekile)**

Ukuqalisa ngohlelo lwe-DREAMS kanye nokuthola lokho asebevele bekuzwile mayelana nohlelo lwe-DREAMS. Lokho yikho esizophenya ngakho ngokuphindaphinda esikhathini esiyiminyaka emibili ngesikhathi sezingxoxo ezingahlelekile.

**Ingabe uke wezwa ngohlelo olubizwa ngokuthi yi-DREAMS?** (phenya ngokusebenzisa amagama ezinhlangano ezisebenzela kuleyo ndawo? Uzwe ngandlelani mayelana nohlelo olubizwa ‘i-DREAMS’? Thola ukuthi babandakanyeka kanjani nokuthi yingani bebandakanyeka? Balubona kanjani lolu hlelo? Yini enhle? Yini embi? Yini abangayenza ngcono? Wabandakanyeka kanjani? Ngubani oweza kuwe? Bazi kanjani ngawe? Bakuqoka ngasizathu sini futhi yiziphi izinyathelo abazithatha?

**Phenya ukuthi yiluphi uhlelo lwe-DREAMS noma uhlobo lokungenelela lohlelo lwe-DREAMS ababandakanyeke kulo?** Uma bengenaso isiqinisekiso – buza ukuthi yiyiphi inhlangano ebanikezela ngohlelo lokungenelela nokuthi yiziphi izinto ababandakanyeke kuzo. Ekuhambeni kwesikhathi, thola ukuthi yiziphi izinhlelo abaqale ukuzibandakanya kuzo nokuthi yiziphi asebeziyekile? Baziyekelani? Yiziphi izinto ezingenziwa kangcono? Izinto abangazithanda kodwa ezingekho?

Nalu uhla lwezinye izinto okungaphenywa ngazo ngqo – mhlawumbe ngokusebenzisa ilogo yenhlangano noma into ebonakalayo yokukhumbuza:

1. *Izindawo Eziphephile maqondana namantombazane/nabesifazane abasebancane*

*Igama lendawo mayelana nohlelo Lwendawo Ephephile]*

1. *Uhlelo lomeluleki osenkantshubomvu (mentor)*

*[Igama lendawo mayelana nohlelo Lokwelulekwa ngumeluleki osenkantshubomvu]*

1. *Izinhlelo Zokuthuthukisa Inhlalakahle*
2. *Okwenziwa Esikoleni - Vhutsilo ne-TALC*
3. Amasevisi okuhlolelwa nokwelulekwa nge-Sandulela ngculazi
4. *Ukunikezwa kwamakhondomu – kwizizinda zezempilo, emphakathini*
5. *Ukwelulekwa ngokokuvimbela inzalo/ukuhlela umndeni*
6. *Ukuhlinzekwa kwezindlela zokuvimbela inzalo/nokuhlinzekwa kwalokho okudingekayo*
7. *Amasevisi emva kokuhlangabezana nodlame (kubandakanya udlame olumaqondana nezocansi, emzimbeni noma emoyeni)*
8. *Izinkozo ezihambelana nezingane ezisencane, njengehora lenjabulo, ukubamba imigqa ebalulekile mayelana nezingane ezigqoke umfaniswano wesikole, njl. njl.)*
9. *Ukuvimbela i-Sandulela ngculazi/i-STI*
10. *Ama-pre-exposure prophylaxis (PrEP) (Okungamaphilisi athathwa ngabesifazane abangenayo i-Sandulela ngculazi ukuze bavimbele ukutheleleka ngayo)*
11. *Ama-Post-exposure prophylaxis (PEP) (Imithi engathathwa ngenhloso yokuvimbela ukutheleleka nge-Sandulela ngculazi emva kokuba sengozini yokutheleleka ngegciwane, kubandakanya isikhathi ngemuva kodlame)*
12. *Ukuhlungelwa (screening) i-STI nokwelashwa*
13. *Mayelana nabesilisa - Ukusokwa kwabesilisa (ukusokwa kwizizinda zezempilo ngokuzithandela)*

**Ingabe abazali /ababheki/abanakekeli benu bayabandakanyeka yini/bayaneseka yini ekuhlinzekweni ngalezi zinhlelo zokungenelela?** *Thola izizathu zokuthi yingani bebandakanyeka noma bekweseka/yingani bengabandakanyeki noma bengakweseki – yingenxa yokuzwela, isib. ukusetshenziswa kwamakhondomu noma kokokuvimbela inzalo? Chaza ukuthi ingabe lokhu kunomthelela yini/noma kuzoba nomthelela ekuqhubekeni kwakho mayelana nokukuthola/nokukusebenzisa lokhu?*

**Enye indlela yokuthola lokhu ongayisebenzisa, ucabanga ukuthi lolu hlelo lokungenelela/ukuba kwakho kulolu hlelo lwe-DREAMS kuyakusiza?** *[Myeke ongenele ucwaningo azikhulumele ngokukhululeka engaphazanyiswa futhi wenze ukuthi akutshele kabanzi uma kwenzeka ekutshela ngezinye izinto ezimayelana nemfundo, ezempilo kanye ne-Sandulela ngculazi/AIDS]*

**Ukuvala**

Bonga ongenele ucwaningo ngesikhathi sabo kanye nangokushiyelana ngolwazi labo. Fingqa umsebenzi osuwenziwe yiqembu locwaningo ukufikela manje bese uchaza ukuthi yini esizolandela mayelana nokuxhumana esikhathini esizayo izikhathi ezingafika kwezingu-10 esikhathini esiyiminyaka emibili ezayo. Yenza isu lokuhlelela ingxoxo yokulandelela elandelayo, engenziwa ngocingo noma ubuso nobuso. Futhi uthathe imininingwane yezindlela ezihlukahlukene zokuxhumana

Buza ukuthi banayo yini imibuzo

Babonge ngesikhathi sabo futhi ubanikeze nokuphuzwayo ubanikeze nemali yokugibela uma begibela.
