# Supplementary material for: Translating DREAMS into practice: Early lessons from implementation in six settings
Source: PLoS One. 2018 Dec 13;13(12):e0208243. doi: 10.1371/journal.pone.0208243 (PMC6292585; doi:10.1371/journal.pone.0208243)
Supplement: S8 File — (DOCX) [file pone.0208243.s008.docx]

**S8 File. DREAMS Impact Evaluation, Semi-structured Key In-depth Interview Guide, South Africa (English)**

**Semi-structured key in-depth interview guide**

**with those delivering services**

**Time:** 1 hour

**Objectives:**

- To assess the extent to which the intervention was delivered as intended, and its immediate effects
- To explore views and experience, barriers and facilitators to implementation with those involved in delivering the interventions
- To understand how implementation and experience of DREAMS is influenced by the differing contexts in which it is delivered, including the influence of other non-DREAMS activities in the target sites

**Participants:**

Identify up to 20 individuals responsible for implementation of DREAMS activities (a mix of men and women and ages if possible) in the area. In addition, include local healthcare workers (n=10-12) and community and youth leaders (approx. 10).

**Venue:** Private spaces; allow participant to identify a safe space.

**Preparations:**

In-depth interview guide, notebook, pencil/pen, recorder, information sheets and consent forms. Interview should be carried out by one researcher.

**Introduction**

Explanation of research: Thank you for agreeing to talk to us.

Administer information sheet

- **Collect participant details**
- **Administer informed consent**
- **Explain that this interview will take no longer than an hour and will be recorded for research use only**

Information to collect to go at the top of the transcript:

**Participant details:**

Name of Interviewer(s): ____________________________

Date: ______________

Location [District, Province]: ­­­­­­­­­­______________________

Interviewee’s Name: _____________________

Position in Community______________________________

Age [in years]: _____________

Sex [circle one]: FEMALE MALE

Tribe: ________________ Nationality: ________________

Use these questions below as a guide but also use the interview as an opportunity to explore any particular issues or events that have happened or that you have heard about in this community.

**Introduction**

1. **How long have you lived in this community?**  *Were you born here? How did you come to stay here?*
2. **Can you tell me about your work here?** [*If applicable] How long have you been doing this job? Do you enjoy your work?*

**SECTION 1: WITH DREAMS Implementers**

**THEME 1: Experiences implementing DREAMS (and DREAMS-like activities)**

1. **Can you please tell me what DREAMS related activities your organisation is involved in?** *Probe- how you got involved in DREAMS? Ask about targets and target groups , Ask how frequent/often the activities are done. Ask if any of their work deals with PrEP specifically if they have heard of PrEP/pills that people who are not infected with HIV can take to prevent HIV infection.*
2. **Can you please tell me how you recruit the target groups?** *Probe – how they identify them, approach them and invite them to participate [Only if they have heard of PrEP above]: Probe as to their opinions as to who should use PrEP, whether they would be willing to prescribe/refer AGYW & ABYM to get PrEP should it be approved for adolescents, how useful they think PrEP is for AGYW, etc.*
3. **When did the roll-out of the DREAMS activities start?**
4. **What preparation work was done before the roll-out?** *Probe - who are the* *staff working on the different activities, their qualification and training?*
5. **What is the coverage and reach of components of DREAMS at different sites?** *Probe- in terms of geographical area covered, wards, isigodi or neighbourhoods – whatever boundary demarcations they are using. Ask how the coverage is/was determined was it by need or other criteria used.*
6. **Can you tell me how you retain participants, how they exit the programs and what happens to them afterwards?** *Probe about drop-outs, those who are not within target group but willing to participate in DREAMS activities etc*
7. **Can you please tell me about the reporting processes?** *Probe financial, monitoring and evaluation etc*

**THEME 2: Perceptions with the DREAMS program**

1. **How do you think DREAMS is experienced by those it targets e.g the adolescent girls and young women, their families and communities?** *Probe whether they think it is a good or bad program - for them and or for those benefiting from it?*
2. **Do you have a sense of whether things have changed since you started DREAMS? If so - how and why?** *Probe – is there any noticed improvement or lack of from those receiving DREAMS, from the community at large or any other changes they may have noticed?*

**THEME 3: Barriers and facilitators of implementing DREAMS**

1. **What are the factors that have been helping and or hindering the successful implementation of DREAMS in your organisation, community and or facility?** *Only probe if the respondent doesn’t respond - for political factors - different government departments, financial factors, lack of/support from families and the young girls, schools, etc*

**SECTION 2: With Community and youth leaders and local healthcare workers**

**THEME 1: Perceptions of HIV prevention, treatment and care services**

1. **Who do you think is most at risk of HIV in this community? Why?** *Probe - Are there certain areas where people take more risks? Use character cards if necessary.*
2. **Do you think many people here know their HIV status?** *Probe -* *What do people say about testing for HIV? Why do you think people would not wish to test for HIV now? Are there certain groups who find it harder to test for HIV? Who are they and why do they find it harder to test? Where do people normally go for HIV testing? Do people ever get tested outside of this community? Has ART changed people’s attitude to HIV testing? Are there certain groups of people that do not wish to test for HIV?*
3. **Do you think that sometimes people test HIV positive but do not get in touch with any HIV services?** *Probe - If this happens, why? What could help such people get in touch with HIV services? Ask how those negative remain negative*
4. **We have been talking to people about what they understand by HIV prevention. For you, what is HIV prevention?** *Probe about the different modes of HIV prevention and popular amongst who?*
5. **Thinking about both established and new HIV prevention strategies, what types of services or interventions do you think are making an impact on HIV prevention in this community? Why?** *First list what they consider older, established strategies and then list newer strategies. Probe about which ones are popular amongst who and why? Probe about medical and traditional male circumcision (including preference, popularity), concept of ‘universal testing and treatment’, treatment of STIs and PMTCT. Probe specifically for DREAMS activities/organisations if it does not come out of the discussion e.g. What are young women using to prevent HIV now? This can lead into 6 and 7*
6. **If it doesn’t come out spontaneously from 5 ask: One of the new forms of HIV prevention is PrEP. Have you ever heard of PrEP? Have you ever heard of pills people who are not infected with HIV can take to prevent themselves from being infected with HIV? What have you heard about it/them? -** Probe for any specific details if the respondent has heard of PrEP- how often people need to take it, how effective it is against HIV/other STIs, side effects, if anyone in the community is using PrEP, how and where can they get it? etc.
   [If a participant has not heard of PrEP, explain that they are pills that people who do not have HIV can take to prevent becoming infected and then proceed with questions 7 and 8]
7. **What groups do you think would benefit from PrEP?** *Probe as to their opinions as to who should use PrEP? what groups they think need PrEP most, what groups should not be given PrEP, probe specifically around the use of PrEP and PrEP like HIV prevention in older adolescents e.g. young women aged 15-19… etc. How would PrEP compare to what young women are using now?*
8. **What are the challenges to introducing new HIV preventions ?** *If it does not come out spontaneously, probe as to what could help or hinder a rollout of PrEP to older adolescents (specifically young women 15-19).*
9. **Who are the most important HIV service providers here? What do they do and why are they important?**

**THEME 2: Knowledge of or exposure to DREAMS (and DREAMS-like) activities**

1. **Have you ever heard about the DREAMS programme?** *Probe - let them tell you what they have heard and or what they understand it is?*
2. **Do you know of any DREAMS intervention organisations in your community***? If they say no or don’t know probe using the organisations eg have you heard about NACOSA/MATCH/AFSA etc in the area or probe using previously mentioned organisations*
3. **Do you know of any activities they do?** *Probe* **-** *How often do they do them, where and with who?*
4. **When did the roll-out of the DREAMS activities start?**
5. **What is the coverage and reach of components of DREAMS at different sites?** *Probe- in terms of geographical area covered, wards, isigodi or neighbourhoods – whatever boundary demarcations they are using. Ask how the coverage is/was determined was it by need or other criteria used if they know.*

**THEME 3: Perceptions of and experiences with DREAMS/organisations providing DREAMS**

1. **How is DREAMS experienced by those it targets, those who deliver it and wider community members? -** *How do people respond towards DREAMS and or DREAMS like services? Probe whether they think it is a good or bad program - for them and or for those benefiting from it?*
2. **Do you have a sense of whether things have changed since you started DREAMS? If so- how and why?** *Probe – is there any noticed improvement or lack of from those receiving DREAMS, from the community at large or any other changes you may have noticed?*
3. **Do you have any DREAMS service providers you work with or support?** *Ask also if they have participated in any DREAMS activities as a recipient/beneficiary? Ask about their experience/attitudes/perceptions.*
4. **Do you think they have an impact on the community? If so, how?**

**THEME 4: Facilitators and Barriers.**

1. **What helps and hinders successful implementation of DREAMS?-** *Only probe if the respondent doesn’t respond - for political factors - different government departments, lack of/support from families and the young girls, schools.*
