# Supplementary material for: Translating DREAMS into practice: Early lessons from implementation in six settings
Source: PLoS One. 2018 Dec 13;13(12):e0208243. doi: 10.1371/journal.pone.0208243 (PMC6292585; doi:10.1371/journal.pone.0208243)
Supplement: S9 File — (DOC) [file pone.0208243.s009.doc]

**S9 File. DREAMS Impact Evaluation, Semi-structured Key In-depth Interview Guide, South Africa (Zulu)**

**UCWANINGO LOKUHLOLA UMTHELELA WOHLELO LWE-DREAMS**

**Uhlaka olungahlelekile lomhlahlandlela osemqoka wesigcawu esijulile semibuzo – nalabo abethula izinkonzo**

**Abangenele ucwaningo**

**Isikhathi:** Ihora eli-1

**Izinhloso:**

- Ukuhlola izinga uhlelo lokungenelela olwethulwe ngayo njengoba bekuhlosiwe, kanye nokushesha kwemithelela lwalo.
- Ukuthola imibono kanye nokuhlangabezwane nakho, izithiyo nalokho okwenze kwaba lula ekuthulweni kohlelo lokungenelela nalabo okuyibo abethula izinhlelo zokungenelela
- Ukuqonda ukuthi izindikimba ezihlukene zikulawula kanjani lokho okuhlangabezwane nakho kanye nokwethulwa kwezinhlelo zohlelo lwe-DREAMS kulezo zindawo lapho zethulwa khona, kubandakanya ukulawulwa ngezinye izinto ezenziwayo ezingaqondene nohlelo lwe-DREAMS kulezo zindawo olwethulwa kuzo.

**Abangenele ucwaningo:**

Khomba abantu abafikela kuma-20 abethula izinhlelo zohlelo lwe-DREAMS (uhlanganise abesifazane nabesilisa neminyaka yobudala uma kwenzeka) kwaHlabisa. Phezu kwalokho, bandakanya abanakekeli bezempilo kuleyo ndawo (abaphakathi kwe-10 kuya kweyi-12) kanye nomphakathi kanye nabaholi bentsha (abalinganiselwa ku-10).

**Indawo:** Izindawo ezingasese; vumela ukuthi ongenele ucwaningo ekhombe indawo ephephile.

**Amalungiselelo:**

Umhlahlandlela ojulile wesigcawu semibuzo, i-notebook, ipensela/umsizi, isiqophamazwi, amakhasi olwazi kanye namafomu olwazi. Isigcawu semibuzo kumele senziwe ngumcwaningo oyedwa.

**Isingeniso**

Incazelo yocwaningo: Siyabonga ngokuvuma ukukhuluma nathi.

Lawula ikhasi lolwazi

- **Qoqa imininingwane yongenele ucwaningo**
- **Lawula imvume enikezwa emva kokuthola kolwazi**
- **Chaza ukuthi lesi sigcawu semibuzo angeke sithathe isikhathi esingaphezu kwehora futhi sizorekhodwa mayelana nocwaningo kuphela**

Ulwazi okumele luqoqwe kumele lube ngenhla kombhalo othathelwe kokukhulunyiwe (transcript):

**Imininingwane yongenele ucwaningo:**

Igama lalowo obuzayo: ____________________________

Usuku: ______________ Isikhathi:_________

Indawo [Isifunda, Isifundazwe]: ______________________

Igama lalowo ophendula imibuzo: _____________________

Isikhundla Emphakathini______________________________

Ubudala [ngeminyaka]: _____________

Ubulili [kokelezela eyodwa]: OWESIFAZANE OWESILISA

Ubuhlanga: ________________ Ubuzwe: ________________

Sebenzisa le mibuzo elapha ngezansi njengomhlahlandlela kodwa usebenzise nezigcawu zemibuzo njengethuba lokuthola okuthile noma izigigaba ezenzekile noma ozwe ngazo kulo mphakathi.

**Isingeniso**

1. **Ungangitshela ngomsebenzi wakho lapha?** [*Uma kufaneleka] Unesikhathi esingakanani wenza lomsebenzi? Ingabe uyawujabulela umsebenzi wakho?*
2. **Unesikhathi esingakanani uhlala kulomphakathi?**  *Ingabe wazalelwa lapha? Uze kanjani lapha?Uma ungahlali kulomphakathi ukuthola kunjani ukusebenza lapha?*

**INGXENYE YOKUQALA: Nalabo abethula izinhlelo zeDREAMS.**

**INGQIKITHI YOKUQALA: Isipiliyoni salabo abethula izinhlelo ze DREAMS nalezo ezifana nazo.**

1. **Ngicela ungitshele ukuthi inhlangano yakho izibandakanya kuziphi izinhlelo ezihlobene nohlelo lwe-DREAMS?** *Buza- wazimbadakanya kanjani nezinhlelo zeDREAMS? Buza ngokuhlosiwe namaqembu ahlosiwe, buza ukuthi zenziwa kangaki izinhlelo zeDREAMS. Buza ukuthi ngabe bayasebenza yini ngoPrEP noma sebeke bezwa yini ngamaphilisi umuntu ongathelelekile ngegciwane lengculaza, angawathatha ukuvikela ukutheleleka ngegciwane lengculaza.*
2. **Ngicela ungitshela niwanxenxa kanjani lawomaqembu ahlosiwe?** *Buza- Babahlonza kanjani futhi nibamema kanjani ukuthi bazimbandakanye? [kuphela uma beke bezwa ngoPrEP ngenhla]: Buzisisa ngokwemibono yabo ukuthi ubani okumele asebenzise uPrEP, noma bengafisa yini ukunikezela ngoPrEP/ukudlulisela amantombazane nabesifazane abasebancane kanye nabafana nabesilisa abasebancane ukuthi bethole uPrEP, kungabe kumele kuvunyelwe yini ukuthi abantu abasebancane banikezwe uPrEP, ingabe bacabanga ukuthi uyasebenza yini kumantombazane nabesifazane abasebancane, njalo njalo.*
3. **Ingabe izinhlelo zohlelo lwe-DREAMS zaqalwa nini ukwethulwa?**
4. **Imaphi amalungiselelo enziwa ngaphambi kokwethula izinhlelo zeDREAMS?** *Buza- Ngobani abasebenzi abenzisa izinhlelo ezahlukahlukene zeDREAMS, iziqu zezemfundo nokuqeqeshwa abanakho?*
5. **Izingxenye zohlelo lwe-DREAMS sezihambe zaze zafinyelela kuphi kuzizinda ezihlukahlukene?**  *Buza uqondise kuleyo ndawo ezifinyelele kuyo, izigceme, isigodi noma ezindaweni ezingomakhelwane – noma yikuphi ukuklanywa kwemingcele okusetshenziswayo. Buza ukuthi indlela esetshenziswayo noma eyasetshenziswa ekutholeni leyo ndawo ezifinyelele kuzo, ingabe kwasetshenziswa indlela yokubheka isidingo noma yokubheka okunye. Yini esetshenziswa abasifazane abasebancane ukuvikela igciwane lesandulela ngculaza manje? Lokhu kungaholela ku 6 no 7.*
6. **Uma kungaziveleli ku 5 buza:** *Enye yezindlela ezintsha zokuvikela igciwane lesandulela ngculaza uPrEP, wake wezwa yini ngo PrEP? Wake wezwa yini ngamaphilisi athathwa abantu abangathelelekile ngegciwane lesandulela ngculaza ukuzivikela ukutheleleka ngegciwane lesandulela ngculaza? Wezwa ini ngakho? Buzisisa noma ngabe iyiphi imininingwane uma ngabe ophendulayo wake wezwa ngoPrEP-kudingeka abantu bawuthathe kangaki, ngabe uyasebenza yini ukuvikela igciwane lesandulela ngculaza nezinye izifo zocansi, imiphumela engemihle kaPrEP, uma ngabe omunye emphakathini esebenzisa uPrEP bawuthola kanjani futhi bawuthola kuphi? Njl*

[Uma ngabe ozimbandakanyile engakaze ezwe ngoPrEP, chaza ukuthi amaphilisi athathwa ngabantu abangathelelekile ngegciwane lesandulela ngculaza ukuvikela ukutheleleka bese uyaqhubeka nomubuzo 7 no 8].

1. **Imaphi amaqoqo ocabanga ukuthi azozuza kuPrEP?** Buzisisa ngokwemibono yabo ukuthi ubani okumele asebenzise uPrEP? Imaphi amaqoqo abacabanga ukuthi adinga uPrEP kakhulu, imaphi amaqoqo okufanele enganikwa uPrEP, buzisisa ikakhulukazi ngokusebenzisa uPrEP kanye nezivimbeli ezifana noPrEP kwasebekhulile isib; abesifazane abasebancane abaphakathi kweminyaka engu 15-19…njl. Ungawuqhathanisa kanjani uPrEP nalokhu abantu besifazane abasebancane abakusebenzisayo njengamanje?
2. **Ngicela ungitshele nibagcina kanjani ababambi qhaza, baphuma kanjani ohlelweni futhi kwenzakalani kubo uma sebephumile?** *Buza- ngalaba abaphuma uhlelo lungakapheli, ngalabo abangekho emaqenjini ahlosiwe kodwa abafisa ukubamba iqhaza ezinhlelweni ze DREAMS nokunye?*
3. **Iziphi izingqinamba ekwethulweni kwezindlela ezintsha zokuvikela igciwane lengculaza?** Umakungaziphumeli, buzisisa ukuthi yikuphi okungasiza ukwethula uPrEP kwasebekhulakhulile (ikakhulukazi abesifazane abasebancane 15-19).
4. **Ngicela ungitshela ngezindlela zokwethula imibiko?** *Buza- ngezezimali, ukulandelela nokuhlola kanye nokunye?**(e.g. Imigudu elandelelwa kusuka efilidini kuya ehhovisi)*

**INGQIKITHI YESIBILI: Imicabango ngohlelo lweDREAMS.**

1. **Uma ucabanga, laba abahlosiwe balwamukela kanjani uhlelo lweDREAMS, isibonelo: amantombazana asemancane nabesifazane abasebencane, imindeni nemiphakathi yabo***? Buza- ngabe bacabanga ukuthi uhlelo oluhle noma olubi kubo nakulaba abahlomulayo kuhlelo lweDREAMS?*
2. **Ngabe ucabanga sengathi lukhona yini uguquko olubonalayo selokhu kuqale izinhlelo zeDREAMS? Uma kunjalo ingani futhi kanjani?** *Buza- ngabe lukhona ushintsho olubonakalayo, ukwentuleka koshintsho kulabo abathola uhlelo lweDREAMS, emphakathini wonkana noma ezinye izinguquko okungenzeka bazibonile?*

**INGQIKITHI YESITHATHU: Izithiyo nokulekelela ukwenziwa kohlelo lwe DREAMS.**

1. Ngabe zikhona izinkinga ozitholayo ngaphakathi kwindlela yokwethula umbiko? *Buza, ngabe usuzitholile izindlela zokubhekana nalobubunzima*, *ngabe washintsha indlela owenza ngayo ngenxa yalezizingqinamba, ngabe unayo indlela yokubhekana nalezingqinamba ngomuso?*
2. **Yikuphi okwenza uhlelo lwe-DREAMS lusetshenziswe ngempumelelo kanye nalokho okwenza ukuthi lungasetshenziswa ngempumelelo enhlanganweni yakho, emphakathini noma esikhungweni sakho?** *Buza kuphela uma umbambi qhaza engaphawuli lutho mayelana nezepolitiki, iminyango ehlukene kaHulumeni, ezezimali, ukwentulelwa kokwesekwa noma ukwesekwa imindeni kanye namantombazane esemancane nezikole,ingqikithi kanye nokunye?*

**INGQIKITHI YESINE: Imicabango nezipiliyoni ngohlelo lwe DREAMS/nezinhlangano ezethula uhlelo lwe DREAMS**

1. **Ingabe bakhona yini abanikezeli benkonzo yohlelo lwe-DREAMS osebenza nabo noma obesekayo?** *Buza futhi ukuthi ingabe bake bazibandakanya yini kunoma yiziphi izinhlelo zohlelo lwe-DREAMS njengabathola/njengabasizakala ngalezi zinhlelo?* *Buza ngalokho abahlangabezana nakho, indlela abazemukela ngayo kanye nemibono yabo.*
2. **Ngabe uyazazi yini ezinye izinhlangano eziletha izinhlelo ezifanayo nezenhlangano yakho (ezixhasiwe u Pepfar/ ezingaxhasiwe u Pepfar)?**
3. **Uma ucabanga izinhlelo zeDREAMS zinawo yini umthelela emphakathini? Uma zinawo, kanjani?**
4. **Yisiphi isipiliyoni sakho mayelana nokuhlanganyela nokunikezela ngezinhlelo ezahlukene kumbambiqhaza oyedwa?**
5. **Ngabe kukhona ukwesekwa ocabanga ukuthi inhlangano yakho ingakunikwa u AHRI?** *Buza- uma kunjalo ikuphi?*
6. **Ngabe inhlangano yakho yanikezela ngamaphasiphothi kumantombazanyana asemancane nabesifazane abasebasha?** *Buza-kube yini isipiliyoni sakho,wanikezela ngamaphasiphothi amangaki?*

**Ukuvala:**

1. **Ngabe unakho ongathanda ukukusho mayelana nengxoxo yethu?**
2. **Ngabe unayo imibuzo ongangibuza yona mayelana nengxoxo yethu?**

**ISIPHETHO SENGXOXO.**
