# Supplementary material for: Translating DREAMS into practice: Early lessons from implementation in six settings
Source: PLoS One. 2018 Dec 13;13(12):e0208243. doi: 10.1371/journal.pone.0208243 (PMC6292585; doi:10.1371/journal.pone.0208243)
Supplement: S10 File — (DOCX) [file pone.0208243.s010.docx]

**S10 File. DREAMS Impact Evaluation, Structured Observation Guide, South Africa (English)**

**Structured Observation guide of interventions**

Observations will be made of 40 randomly selected DREAMS interventions in a range of settings, such as schools, safe spaces, and health facilities. We will focus on how participants interact with the service/material, and note what works well and what less well.

**Objectives**

- Observe a sample of the interventions delivered in context
- Observe the ways in which DREAMS is delivered and received, and with what quality and intensity, via structured observations (using observation check lists).

**A: Health facility/Organization/Safe spaces and schools observation form**

| Date |  |
| --- | --- |
| Time started |  |
| Time ended |  |
| Observer |  |
| Results of observation |  |
| Name & address of health facility/Organization/School |  |
| Name of Venue |  |
| Services offered and activities conducted |  |
|  | |
| Targets:  DREAMS Geographical coverage/ areas/ wards/locations covered:  **Resources available:**  Tools used:  Human resources:  Financial resources/budget:  Other resources:  Infrastructure for office and working space:  Computers and internet:  Telephones:  Administration support:  DREAMS Timeline - Financial Year:  Year 1 DREAMS  Year 2 DREAMS  Year 3 DREAMS | |

**B. Physical environment**

| **Where are the DREAMS activities delivered?**  At home  School  Community hall  Clinic facility  Mobile unit  Specify……………………………………………………………………. |  |
| --- | --- |
| **Which activities are being delivered? List them** |  |
| Is there a signboard that mentions the facilities operating hours? | Yes No N/A |
| Does it mention hours for DREAMS interventions/services? | Yes No N/A |
| **Does the waiting/receiving area:** Have adequate and comfortable seating? | Yes No N/A |
| Describe the set-up of the room where the DREAMS activities are taking place?  Look out for:  Have drinking water?  Does it look overall friendly and welcoming? Clean?  How is the sitting arrangement like?  How is the atmosphere like – tense, exciting or boring? |  |
| **Describe the interaction between the facilitators and the participants?** |  |
| Does the facilitator look confident and knowledgeable in his/her subject area? |  |
| How is the intervention delivered?  Is it participatory?  One on one  Group sessions  Community  Road shows |  |
| How many participants are there? |  |
| What kind of materials are they using for interaction? e.g  Papers  Writing board  Markers  Colours  Booklets  Audio visuals – radio, TV etc  *If in a clinic facility; describe the set-up only as you cannot be part of the consultation* |  |

**C. Privacy and confidentiality of venue/setting DREAMS interventions are delivered**

| **Check for visual and auditory privacy features** |  |
| --- | --- |
| Communication between reception staff and visitors is private and cannot be overheard, including from the waiting room. | Yes No NA |
| In the offices/examining rooms, there is a screen to separate the examination area from the consultation area. | Yes No NA |
| No one can see or hear the client from the outside during the consultation or counselling/mentorship – unless delivered in a group. | Yes No NA |
| If activity not clinical or in a clinic setting: Does the meeting place for activity private and quiet to facilitate smooth delivery of intervention and activities? | Yes No NA |
| Does the venue look conveniently situated for the recipients? | Yes No NA |
| **Check for confidentiality procedures and their application in practice.** |  |
| Information on the identity of the adolescent and young women and the presenting issue are gathered in confidence during registration. | Yes No NA |
| Adolescent and young women are offered anonymous registration if they wish. | Yes No NA |
| Records/information is kept in a secure place, accessible only to authorized personnel. | Yes No NA |
| The registers are kept under lock and key outside operating hours. | Yes No NA |
| For electronically stored information, measures are applied to prevent unauthorized access. | Yes No NA |

**D. Registers, tools, records, guidelines, SOPs, training records etc.**

| **Check to see the following registers, tools and records.** | Yes No NA |
| --- | --- |
|  |  |
| The register on the number of DREAMS beneficiaries by activities | Yes No NA |
| Register with M&E indicators |  |
| The reporting forms have a format that allows the presentation of data disaggregated by age and sex. | Yes No NA |
| Referral register | Yes No NA |
| Check and record the data elements recorded in each register and list eg  Sex  Age  Date of Birth  ID |  |
| Check frequency with which the different activities/sessions/interventions are delivered |  |
| Check the materials that are being used for delivery of the DREAMS interventions and list the main topic areas/themes covered by each |  |
| Check the Standard Operating Procedures available and list them here |  |
| Check the guidelines they are currently using and the dates and list here |  |
| Check for training register of facilitators and list the trainings offered and the ones planned for |  |

Observer notes

………………………………………………………………………………………………………………………………………………………………………………………………………………………………………………………………………………………………………………………………………………………………………………………………………………………………………………………………………………………………………………………………………………………………………………………………………………………………………………………………………………………………………………………………………………………………………………………………………………………………………………………………………………………………………………………………………………………………………………………………………………………
