# Supplementary material for: Translating DREAMS into practice: Early lessons from implementation in six settings
Source: PLoS One. 2018 Dec 13;13(12):e0208243. doi: 10.1371/journal.pone.0208243 (PMC6292585; doi:10.1371/journal.pone.0208243)
Supplement: S11 File — (DOC) [file pone.0208243.s011.doc]

**S11 File. DREAMS Impact Evaluation, Structured Observation Guide, South Africa (Zulu)**

**Ucwaningo Lokuhlola Umthelela Wohlelo Lwe-DREAMS**

**Umhlahlandlela Wokuqapha Ohlelekile wezinhlelo zokungenelela**

Ukuqapha kuzokwenziwa ezinhlelweni zokungenelela zohlelo lwe-DREAMS ezingu-40 ezikhethwe ngokungahlelekile ezindaweni, ezinjengezikole, izindawo eziphephile kanye nakuzizinda zezempilo. Sizobhekisisa indlela ababambiqhaza abasebenzisa ngayo izinsiza , futhi sibheke lokho okusebenza kahle nalokho okungasebenzi kahle .

**Izinhloso**

- Sizoqapha isampula lezinhlelo zokungenelela elethulwa kwindikimba
- Siqaphe indlela uhlelo lwe-DREAMS olwethulwa ngayo nolwamukelwa ngayo, nokuthi lwethulwa ngezinga elihle nangamandla anjani, ngokusebenzisa ukuqapha okuhlelekile (ngokusebenzisa uhlu lokuhlola ukuqapha).

**A: Isizinda sezempilo/Inhlangano/Izindawo eziphephile kanye nefomu yokuqapha yezikole**

| Usuku |  |
| --- | --- |
| Isikhathi ekuqalwe ngaso |  |
| Isikhathi ekuqedwe ngaso |  |
| Umqaphi |  |
| Imiphumela yokuqapha | 1 Kuphothuliwe  2 Kusaphothulwe ingxenye  Wenqabile |
| Igama nekheli lesizinda sezempilo/Inhlangano/Isikole |  |
| Igama leNdawo: |  |
| Izinsiza ezikezwayo kanye nezinhlelo ezethulwayo |  |
|  | |
| Okuqondiswe kukho:  Indawo lapho okwenziwa uhlelo lwe-DREAMS/izindawo/izigceme/amalokishi:  **Ukubakhona kwezinsiza:**  Amathuluzi asetshenzisiwe:  Okuphathelene nabantu:  Izinsiza zezezimali/isabelomali:  Ezinye izinsiza:  Ingqalasizinda sehhovisi nesendawo yokusebenzela:  Amakhompyutha ne-inthanethi:  Izingcingo:  Ukwesekwa kwezokuphatha:  Isikhathi sohlelo lwe-DREAMS – Unyaka Wezimali:  Unyaka woku-1 wohlelo lwe-DREAMS  Unyaka wesi-2 wohlelo lwe-DREAMS  Unyaka wesi-3 wohlelo lwe-DREAMS | |

**B. Isimo sendawo**

| **Izinhlelo zohlelo lwe-DREAMS zethulwa kuphi?**  Ekhaya  Esikoleni  Ehholo lomphakathi  Esizindeni somtholampilo  Kumahamba nendlwana  Chaza……………………………………………………………………. |  |
| --- | --- |
| **Yiziphi izinhlelo ezethulwayo? Yenza uhlu lwazo** |  |
| Ingabe likhona yini ibhodi lophawu elikhombisa ukuthi izizinda zisebenza ngamaphi amahora? | Yebo Cha Akukho okukhethwayo |
| Ingabe liyasho yini ngamahora okwenziwa kwezinhlelo zokungenelela/amasevisi ohlelo lwe-DREAMS? | Yebo Cha Akukho okukhethwayo |
| **Ingabe indawo okulindwa kuyo/yokwemukela abantu:** Inendawo yokuhlala eyenele nohlala kuyo ngokukhululeka? | Yebo Cha |
| Chaza mayelana nokuhleleka kwegumbi lapho kwethulwa khona izinhlelo zohlelo lwe-DREAMS?  Bheka lokhu okulandelayo:  Inawo yini amanzi okuphuza?  Ingabe isimo sinobungane yini futhi siyakwemukela? Ihlanzekile?  Ukuhleleka kwendawo yokuhlala kubukeka kanjani?  Sinjani isimo - sidangele, sinesasasa noma sinesithukuthezi? |  |
| **Chaza ngokusebenzisana phakathi kwabagqugquzeli nabangenele ucwaningo?** |  |
| Ingabe umgqugquzeli ubukeka ezethemba futhi enolwazi emkhakheni wakhe? |  |
| Izinhlelo zokungenelela zethulwa kanjani?  Ingabe abangenele ucwaningo bayabandakanyeka yini?  Zethulelwa umuntu ngamunye  Imihlangano yeqoqo  Umphakathi  Imikhankaso |  |
| Bangaki abazibandakanyayo abakhona? |  |
| Basebenzisa izinto ezinjani mayelana nokuxhumana? isib.  Amaphepha  Ibhodi lokubhalela  Ama-marker  Imibala  Izincwajana  Imithombo ebukelwayo nelalelwayo – umsakazo, i-TV, njl.  *Uma kungumtholampilo, chaza indlela okuhleleke ngayo kuphela njengoba ungeke ukwazi ukuba yingxenye yokweluleka* |  |

**C. Ubumfihlo nengasese lendawo lapho izinhlelo zokungenelela zohlelo lwe-DREAMS zethulwa kuyo**

| **Hlola izimo zobumfihlo bokubonakala nokuzwakala** |  |
| --- | --- |
| Ukuxhumana phakathi kwabasebenzi abamukela abantu kanye nabahambeli kwenzeka endaweni engasese futhi abalalelwa ngabanye abantu uma bekhuluma, kubandakanya ukusuka egunjini lokulindela. | Yebo Cha Akukho okukhethwayo |
| Emahhovisi/emagunjini okuhlola, kukhona iskhrini esihlukanisa indawo okuhlolelwa kuyo nendawo okwelulekwa kuyo. | Yebo Cha Akukho okukhethwayo e |
| Akekho obona ikhasimende nolizwayo engaphandle ngesikhathi sokweluleka - ngaphandle uma ukwelulekwa kwethulelwa iqembu. | Yebo Cha Akukho okukhethwayo |
| Uma uhlelo lungekho mayelana nezokwelapha noma endaweni yezokwelapha: Ingabe indawo okuhlanganelwa kuyo mayelana nokwethulwa kohlelo ingasese yini futhi ayinamsindo yini ekwethuleni ukungenelela nezinhlelo ngaphandle kokuphazamiseka? | Yebo Cha Akufanelekile |
| Inagbe indawo ibukeka ibafanele labo abemukela izethulo? | Yebo Cha Akukho okukhethwayo |
| **Hlola izinqubo zezemfihlo kanye nokusetshenziswa kwazo.** |  |
| Imininingwane mayelana nomazisi wamantombazane nabesifazane abasebancane namayelana nokwethulwayo kuqoqwa ngendlela eyimfihlo ngesikhathi sokubhalisa. | Yebo Cha Akukho okukhethwayo e |
| Amantombazane asemancane nabsesifazane abasebancane ukubhaliswa kwabo kwenziwa ngendlela engeke ibadalule uma befisa kanjalo | Yebo Cha Akukho okukhethwayo |
| Ukuqopha /ulwazi lugcinwa endaweni evikelekile, efinyelelwa ngumsebenzi onegunya kuphela. | Yebo Cha Akukho okukhethwayo |
| Amarejista ayakhiyelwa uma sekudlule isikhathi sokusebenza. | Yebo Cha Akufanelekile |
| Mayelana nolwazi olukukhompyutha, kusetshenziswa izindlela zokuvimbela ukuba kufinyelelwe kulo ngabantu abangagunyaziwe. | Yebo Cha Akukho okukhethwayo |

**D. Amarejista, amathuluzi, imihlahlandlela, ama-SOP, amarekhodi oqeqesho, njl.**

| **Hlola ukuze ubone lawa marejista, amathuluzi kanye namarekhodi okulandelayo.** | Yebo Cha Akukho okukhethwayo e |
| --- | --- |
|  |  |
| Irejista mayelana nenani labantu abathola uhlelo lwe-DREAMS ngezihlelo | Yebo Cha Akukho okukhethwayo |
| Irejista elinezinkomba ze-M&E. |  |
| Amafomu okubika anesimo esivumela ukuthi ukwethulwa kolwazi kwehlukaniswe ngezingxenye ngeminyaka yobudala nangobulili | Yebo Cha Akukho okukhethwayo |
| Irejista lokudlulisa | Yebo Cha Akukho okukhethwayo |
| Hlola bese urekhoda izingxenye zolwazo olubhalwe kuleyo naleyo rejista nasohlwini, isib.  Ubulili  Iminyaka  Usuku lokuzalwa:  Umasizi (ID) |  |
| Hlola izinga izinhlelo ezihlukahlukene/imihlangano/ukungenelela okwethulwa ngalo |  |
| Hlola izinto ezisetshenziswa ekwethulweni kwezinhlelo zokungenelela zohlelo lwe-DREAMS bese wenza uhlu lwezingxenye zezihloko/zezindikimba ezibalulekile okubhekelelwe yilolo nalolo hlelo |  |
| Hlola Izinqubo Zamazinga Okusebenza ezikhona bese uzibhala khona lapha |  |
| Hlola ukuthi ingabe imihlahlandlela abayisebenzisayo njengamanje bese uhlola nezinsuku bese ukubhala lapha |  |
| Hlola irejista loqeqesho labagqugquzeli bese wenza uhlu loqeqesho olunikezwayo kanye nalolo qeqesho oluhlelelwe |  |

Amanothi womqaphi ……………………………………………………………………………………………………………………………………………………………………………………………………………………………………………………………………………………………………………………………………………………………………………………………………………………………………………………………………………………………………………………………………………………………………………………………………………………………………………………………………………………………………………………………………………………………………………………………
