# Supplementary material for: Translating DREAMS into practice: Early lessons from implementation in six settings
Source: PLoS One. 2018 Dec 13;13(12):e0208243. doi: 10.1371/journal.pone.0208243 (PMC6292585; doi:10.1371/journal.pone.0208243)
Supplement: S12 File — (DOC) [file pone.0208243.s012.doc]

**S12 File. DREAMS Impact Evaluation, Guide for Transect Spiral Walk, South Africa**

**Rapid, participatory, community mapping**

Rapid, participatory, community mapping - this method will be used in the DREAMS areas (12-15 days per area) to quickly gain a broad and comparable understanding of the social context for adolescents and young people and the reach and coverage of the AGYW services at baseline and after two years of DREAMS intervention. This will rely on use of rapid appraisal methods with participant observation and short interviews.

**Objectives:**

- Observe activities and movement of different age and gender groups.
- Gain a rapid understanding of the social context for adolescents and young people and the reach and coverage of the AGYW services at baseline and after two years of DREAMS intervention
- Identify places for observations during fieldwork.

**RA Roles:** One RA to take nearest BSID whilst other writes a description of the place. Both RAs to chat to local people during the transect spiral.

**Materials:** Transect Spiral Guide, study site map, Data Capture Sheet, Transect Walk Observation Guide, few sheets of A4 paper, notebook, pens.

**Time of Activity:** Afternoon and Morning

**Length of Activity:**  One afternoon and one morning.

**Venue:** No venue; rather going in concentric circles from the ‘centre’ of community

**Flow:**

***At the start:***

- Set off from a central place and move from there from place to place.

***During the walk:***

- Observe the places passed, noting conditions in different sub-areas and schools and health care centres, activities and movements of people and livelihood options.
- Ask others who they pass probing questions about the different important places and activities/organisations doing HIV prevention and transmission interventions in the community. Where do young people gather? What do young people do? Make rough notes or sketches in the notebook.
- Look out for the types of places suggested by the Transect Walk List of Places i.e. health facilities, commercial premises, places of worship, recreational spaces, boundary landmarks, graveyard, etc (see TRANSECT WALK OBSERVATION CHECKLIST) where possible DREAMS intervention activities are being done. Stop at each such place.
- For each place:
- Record on the DATA CAPTURE SHEET.
  - The type of place (selected from the TRANSECT WALK OBSERVATION CHECKLIST) and the name of this particular place (if applicable).
  - A description is given of: time, type of gathering place; size of building; what people are doing; the approximate number of people; the age mix of people there.
  - Assess whether the place is a possible observation point to return to on the following days.
  - If people ask, explain that the team are doing this for the purposes of understanding the community before a new projects begin. Engage in brief informal conversation, making field notes afterwards.

**Data Collected and Stored:**

- Make a rough sketch of the transect walk on blank A4 paper, indicating all the places plotted and observed during the walk.
- Complete the Transect Walk Activity Report Form on the same days as carrying out the walks, describing the process and the findings (from notes made in the note book) as they relate to activities and mobility of people and perceptions of HIV transmission and prevention.
- Record all the words for HIV, risky behaviours, adolescents, young people, DREAMS activities, organisations (vernacular, English, slang, street language) that you have heard during this observation.
- Complete the data capture sheet (tidy up, fill out).
- Type field notes and activity report via shared folder.
- Submit hard copies to team leader.
