# Supplementary material for: Translating DREAMS into practice: Early lessons from implementation in six settings
Source: PLoS One. 2018 Dec 13;13(12):e0208243. doi: 10.1371/journal.pone.0208243 (PMC6292585; doi:10.1371/journal.pone.0208243)
Supplement: S13 File — (DOC) [file pone.0208243.s013.doc]

**S13 File. Community Mapping Guide for Formative Research in Zimbabwe**

The main purpose of community mapping is to identify different *geographical* locations where sex is sold in the area and their *typologies* (ie street based, venue based, social stratification of sex workers). Further aims are to describe different kinds of sex work available, learn how the social networks of sex workers are organised, and assess the feasibility of conducting an RDS survey among this population. It would also be useful to get some detailed information regarding willingness to participate in the survey and to recruit peers, and the suitability of the survey site.

The community mapping will be conducted as a rapid assessment, and will use the following methods: (1) Group discussions with trained peer educators, and other “natural groups” of sex workers located during fieldwork; (2) Community informant interviews with individuals likely to be able to identify sex work locations and typologies; and (3) direct observation of venues across Area. All discussions will be informal, are unlikely to last more than 15 minutes, and will not be audio-recorded, although notes will be taken.

Prior to travelling to the field, it would be useful to obtain the following:

- Map of the district, including the entire “catchment area” for the project
- List of NGO or other health projects operating in the area, esp those related to HIV prevention and treatment or that work with sex workers
- List of stand-along HIV testing sites or other specialist clinics that sex workers might use

Discussion groups

It would be worth bringing the trained peer educators together in 1-2 groups to introduce the mapping exercise and elicit their perspectives first. The focus for these discussions would be to get general information on the areas where sex workers can be found in the city (including both where they work and where they live), organisation of the sex industry, and a list of different types of sex workers and the main differences between them and whether or not they mix (socially or professionally). Peer educators might also be able to refer us to other community informants and may be able to help recruit sex workers found at various venues for impromptu discussions.

Community Informants:

- Sex workers in different locations
- Bar/shebeen owners/workers
- Vendors near bars
- Other large entertainment venues (night clubs)
- Hotel owners
- Taxi drivers
- Health service staff/NGO workers who provide services to SW

Locations in the area will be identified through a mix of initial information provided by peer educators, snowball sampling through community informants, and observation while driving through the urban sector of the district. A list will be made of areas where a significant amount of sex work takes place, and locations marked on a map. Community informants will be identified on site and approached for a brief conversation. In addition to the question topics listed below, informants will be asked to suggest other locations in the area that would be investigating, or individuals who might have useful knowledge on the local organisation of sex work.

Issues to discuss:

1. Where in the area do men go to purchase sex/ meet sex workers? What *kinds* of places, and in which exact *locations*?
2. What kinds of sex workers are there *in this venue/location*?
3. What is sex work like here? (ie how do clients make arrangements with sex workers? Where do they go to have sex?)
4. When is it busiest in this location?
5. Are there different kinds of sex workers in the area? How are they referred to and what are the differences between them? (ie price, location, services offered)
6. Do different kinds of women engage in different forms of sex work?
7. Do sex workers stay in the same place or move around, e.g. work in different establishments at different times?
8. Would sex workers know each other or keep separate? Why?
9. What other places should we visit if we want to know about sex work in the area? Whom should we speak to?

Where sex workers are in evidence in a venue, it would be worth approaching 2-3 at a time for an informal chat. Peer educators may be able to accompany field workers during the mapping to help arrange discussions. In addition to the questions listed above, sex workers should provide additional information on:

1. Types of sex workers that they personally know, and where they work
2. Roughly how many sex workers they know fairly well, and see each month
3. Types of *clients* that they see (to get their terms for “regular” “new” “casual” etc.)
4. Areas that they travel to, e.g. for work, to live in, or to go shopping in
5. When they are working and busiest, when they have more free time
6. Willingness to participate in a survey for compensation
7. Willingness to recruit other sex workers into a survey
8. Willingness to travel to the clinic to fill out a questionnaire
9. Willingness to provide a dry blood sample for anonymous testing
10. What times of day would be most convenient to participate in a survey

Mapping will be the first stage of formative research, and will be followed by longer individual interviews with sex workers from across the geographical areas and types of sex work identified. These interviews will last 30-40 minutes and explore topics relevant to the RDS survey, including social support and networking, experience of sex work, and access to and use of health services. This information will help refine the RDS instrument and recruitment protocol.
