# Supplementary material for: Translating DREAMS into practice: Early lessons from implementation in six settings
Source: PLoS One. 2018 Dec 13;13(12):e0208243. doi: 10.1371/journal.pone.0208243 (PMC6292585; doi:10.1371/journal.pone.0208243)
Supplement: S14 File — (DOCX) [file pone.0208243.s014.docx]

**
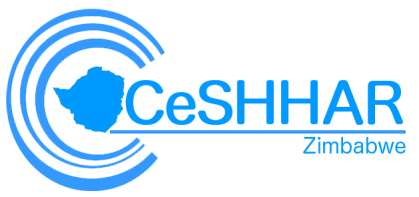
**

**The Centre for Sexual Health and HIV AIDS Research Zimbabwe (CeSHHAR Zimbabwe)**

**9 Monmouth Road, Avondale West**

**Harare, Zimbabwe**

**Phone +263 4 333393; 308042**

21 Rowland Square, Milton Park,

Harare, Zimbabwe

**________________________________________________________________________**

**S14 File. DREAMS Mapping Topic Guide, Zimbabwe**

| **Areas of Inquiry** | **Specific Topics** | **Suggested Probes** |
| --- | --- | --- |
| **Background** | Where is she from? How old she is?  How long has she been in the site? | - Tell me about your life here? - Where do you live what do you do in life, who do you live with? |
| **Participation in sex work** | How long has she been a sex worker  How she got to start selling sex  Has she always worked in this place; does she work anywhere else?  What is the work like? | - Tell me about your work here - How do you meet your clients and where do you go? - When is it busy/ quiet? - What kinds of partners or clients do you have? - What are your charges? - Whom do you work with? (other sex workers, managers, bar workers) |
| **Sex work in the different sites.** | Where else do adolescent girls and young women sell sex in this place?  What are the differences between types of sex workers? | - What kinds of places do other young women sell sex? - What is the busiest place in this location? - Explain to me what makes the place busy? - Do you stay at one place at a time or move around and work in different places at different times? - Do women engage in different forms of sex work? (Explain) - How are they referred to and what are the differences between them? (ie price, location, services offered) |
| **Places where sex work is done** | Ask places where sex work is done | - Where do you have sex with your clients? - Tell me about the different type of clients you see? - Where do other young women who sell sex have sex with their clients? - What other places should we visit if we want to know about sex work? |
| **Social relationships** | Do they socialize with other sex workers? | - Tell me about the different types of sex workers that you personally know? - Do you work together or keep separate? |
| **Willingness to participate in research** | *Discussion about the survey and whether she would be willing to go to fill out a questionnaire, give a blood sample,whether she would be comfortable to have the interview at home or at a nearby site for a cash incentive.* | - What time of day would be convenient for an interview with you? - Are you willing to give an anonymous blood sample? - Who might you ask to participate? - Would your friends agree to go? Why or why not? - Would you refer us to other young people who sell sex in your area? |
