# Supplementary material for: Translating DREAMS into practice: Early lessons from implementation in six settings
Source: PLoS One. 2018 Dec 13;13(12):e0208243. doi: 10.1371/journal.pone.0208243 (PMC6292585; doi:10.1371/journal.pone.0208243)
Supplement: S3 Table — (DOCX) [file pone.0208243.s018.docx]

**S3 Table. Characteristics of the implementation process across DREAMS evaluation sites**

| **Approaches to implementation** | **South Africa**  **(KwaZulu-Natal)** | **Kenya – urban** | | **Kenya – rural sub-county** | **Zimbabwe** | |
| --- | --- | --- | --- | --- | --- | --- |
|  |  | **Informal Settlement A** | **Informal Settlement B** |  |  |  |
|  |  |  |  |  | **District A** | **District B** |
| **Number of Implementing Partners (IP) in this DREAMS site** | 6 (with some IPs further sub-contracting community-based organisations) | 1 | 1 | 2, each with distinct remits: 1 IP focused on 10-14 yr olds and the other on 15-24 yr olds | 6 | 6 |
| **National level coordination** | South African National AIDS Council, National Department of Health , Department of Social Development, Department of Basic Education, PEPFAR Coordination office, USAID, CDC, Peace Corps, Civil Society | CDC coordinating CDC partners, USAID coordinating USAID partners, DREAMS co-chairs, Activity manager for each IP from CDC | | Kenya National AIDS Control Council, USAID, CDC, Department of Defence, Peace Corps | Zimbabwe National AIDS Council, Ministry of Health, USAID | |
| **Body responsible for coordination of IPs in this setting** | SANAC DREAMS Provincial coordinator, and District Support Partner – district coordinator | n/a (only 1 IP) | n/a | US Govt agencies, e.g., CDC | Provincial AIDS Coordinator - District Secretariat  *(different levels of organisation & initiative & leadership in both places has made big difference in implementation of DREAMS)* | |
| **Is there one lead IP?** | n/a  There is a District Support Partner | Yes | Yes | Yes: 1 per age group | Yes | Yes |
| **Package of interventions** | All interventions in DREAMS core package, aligning with ‘She Conquers’ packages by age and need | All interventions provided by IP or referred to Govt services.  Minimum packages by age and need specified in July 2017 | | All interventions provided by 2 IPs or referred to Govt services. Some interventions sub-contracted to CBOs, e.g., for  educational support services.  Minimum packages by age and need defined in July 2017 | All interventions in DREAMS core package  ‘Primary and Secondary Interventions’ by sub-populations specified in July 2017 | |
| **Earliest interventions / components available** | -Condom promotion for AGYW and males  -HIV testing and counselling (HTC)  -STI screening & treatment  -ART  -ASPIRES  -School-based | -Condom promotion for AGYW and males  -Education subsidies  (because already available; required transitioning from another USG programme) | -Condom promotion  -HTC  -STI screening & treatment  -Safe spaces and mentoring  -HIV education in safe spaces & schools  -Targeting male sexual partners for HTC, condom promotion & ART | -Condom promotion  -HTC  -STI screening & treatment  -Safe spaces and mentoring  -HIV education in safe spaces & schools  -Targeting male sexual partners for HTC, condom promotion & ART | HIV testing  ART  School-based  Educational subsidies | |
| **Components that took longest to implement** | -PREP (launched in Nov)  -Targeting male sexual partners for HTC, condom promotion & ART  -Psychosocial support for GBV | -Cash transfers  -Financial literacy  -Savings groups  -VMMC  -Targeted HTC for males  -PrEP (national policy introduced in second year) | -Cash transfers  -Violence prevention & gender norms (SASA! *–* training & ethics approval was required first)  -Post-violence legal care  -Emergency contraception  -PrEP (national policy introduced in second year) | -PrEP  -Cash Transfers  -Educational subsidies  -combined socio-economic programs | PrEP | |
| **Services not yet available or no uptake by Aug 2017** | None | -Microfinance lending  -UNESCO’s comprehensive sexuality education curriculum in schools  -No referrals to date for:  --Emergency contraception (as a stand-alone service none so far but some uptake as a component of PEP)  --Post-Exposure Prophylaxis for post-violence care | -Microfinance lending  -UNESCO’s comprehensive sexuality education curriculum in schools  -No referrals to date for:  --Emergency contraception  --PEP | -UNESCO’s comprehensive sexuality education curriculum in schools  -Media & policy component, e.g., MTV Shuga | Low uptake of PEP (showing too late, >72 hours) |  |
| **Process for recruitment of AGYW into DREAMS** | Geographical and hot spot mapping of vulnerable areas | Enrolled using the Girl Roster method, a household census to enumerate the universe of high-risk AGYW in the geographical area, supplemented with referrals from community-based organisations and implementing partners | | | -Registry for cash transfers (prioritised)  -IP Screening  -Stop the Bus campaign | -Registry for cash transfers (prioritised)  - IP screening |
| **Layering of services** | Layering guidance provided to IPs in Sept 2017  DREAMS Passport not adopted | Minimum package of services introduced July 2017, by age and need  Mentors responsible for group of DREAMS clients and refer based on needs  (In some places, DREAMS Passport considered too expensive to adopt, and coming mid-way through, IPs were not convinced of usability. Used an internal tool with the mentors instead) | | | -DREAMS Passport  -Transport vouchers with referrals  -Screening & Referral Guidelines; --Community facilitators  -IPs overseeing sub-grants to CBOs, good coordination of multiple partners | |
| **Tracking referrals** | n/a | Each client has a DREAMS badge with her DREAMS ID; service providers note ID when making & accepting referrals (for services not provided by the sole IP) | | -DREAMS ID; safe spaces within school so easy to monitor; mentor roll-call and know ID | MOHCC referral sheets  Unique ID Code – generated for DREAMS | |
| **Targets** | - AGYW HIV Testing servies (HTC): 11,784  - Post-violence Care: 286  - SRH Services: 11,430  - PrEP: 67  - Social Asset Building: 1,946  - Community Mobilisation & Norms Change: 4,168  - School-based HIV & Violence Prevention: 12,224  - Parenting & Caregiver: 1,478  - Material Support: 4,447  - Combination Socioeconomic Approaches: 1,108  - Male Sex Partner HIV Testing: 8,618 | Yr 1: targets set for enrolment and for each intervention/service provided  Yr 2: targets added for minimum packages by age | | - per intervention but layering introduced later - intervention targets vary with the layering targets | Yr 1: targets set for each IP – services provided & referrals made  Yr 2: targets added for primary and secondary intervention packages | |
| **Which targets were met soonest?** | HIV testing and counselling | HIV testing and counselling | | Education subsidies |  | |
| **Routine/programme monitoring** | IPs submit monthly reports to central database: ‘DIMES’ – an adaptation of DHIS for DREAMS | IPs submit monthly reports on enrolment and services to central database – created for DREAMS and managed by CDC/UCSF | | | IPs submit monthly reports (targets) to DHIS2 database adapted for DREAMS | |
| **Refinements or ‘course-corrections’/programme adjustment based on early learning** | Several IP meetings to discuss progress and encourage layering and referral | Introduction of the DREAMS badge & ID to track referrals  Clarification of minimum packages by age / need to ensure layering of services | | | Introduction in Year 2 of:  - Referral protocol  - Passport  - Screening and referral guidelines  - Primary and Secondary interventions for sub-populations | |
