# Supplementary material for: Translating DREAMS into practice: Early lessons from implementation in six settings
Source: PLoS One. 2018 Dec 13;13(12):e0208243. doi: 10.1371/journal.pone.0208243 (PMC6292585; doi:10.1371/journal.pone.0208243)
Supplement: S1 Fig — (PDF) [file pone.0208243.s019.pdf]

PEPFAR DREAMS : the commitment remains both in funding and in focus

DREAMS
WORKING TOGETHER FOR AN A.I.D.S-FREE FUTURE FOR GIRLS & WOMEN

	Year 1	Year 2	Year 3 (COP 17)	Year 4 (Planned COP 18--TBC)
DREAMS		FY17	FY18	FY19
Kenya	19,742,670	19,742,670	29,242,670	29,242,670
Lesotho	7,017,660	7,017,660	10,017,660	10,017,660
Malawi	7,017,790	7,017,790	7,017,740	7,017,740
Mozambique	10,195,770	10,195,770	10,195,770	10,195,770
South Africa	33,323,381	33,323,381	33,323,381	33,323,381
Swaziland	5,009,695	5,009,695	5,009,695	5,009,695
Tanzania	8,163,178	8,163,178	18,163,178	18,163,178
Uganda	15,717,403	15,717,403	15,717,403	15,717,403
Zambia	8,124,208	8,124,208	13,124,208	13,124,208
Zimbabwe	10,310,785	10,310,785	15,310,785	15,310,785
DREAMS Innovation Challenge		80,000,000		
DREAMS-like				
Botswana			4,792,016	4,792,016
Cote D'Ivoire			10,000,000	10,000,000
Haiti			2,000,000	2,000,000
Namibia			10,000,000	10,000,000
Rwanda			5,000,000	5,000,000
Total	124,622,540	204,622,540	188,914,506	188,914,506

Determined | Resilient | Empowered | AIDS-Free | Mentored | Safe

Presented by Ambassador Birx, United States Global AIDS Coordinator, 1 June 2018, at the Center for Global Development, Washington DC
